# Thalidomide-based regimen shows promising efficacy in large granular lymphocytic leukemia: a multicenter phase II study

**DOI:** 10.1038/s41392-025-02164-4

**Published:** 2025-03-12

**Authors:** Ying Yu, Yuxi Li, Rui Cui, Yuting Yan, Fei Li, Yan Chen, Tingyu Wang, Xiaoli Hu, Yaqing Feng, Tengteng Yu, Yanshan Huang, Jingwen Sun, Rui Lyu, Wenjie Xiong, Qi Wang, Wei Liu, Gang An, Weiwei Sui, Yan Xu, Wenyang Huang, Dehui Zou, Huijun Wang, Zhijian Xiao, Jianxiang Wang, Lugui Qiu, Shuhua Yi

**Affiliations:** 1 State Key Laboratory of Experimental Hematology, National Clinical Research Center for Blood Diseases, Haihe Laboratory of Cell Ecosystem, Institute of Hematology & Blood Diseases Hospital, Chinese Academy of Medical Sciences & Peking Union Medical College, Tianjin, China; 2 Tianjin Institutes of Health Science, Tianjin, China; 3 Department of Hematology, Tianjin First Center Hospital, Tianjin, China; 4 Department of Hematology, The First Affiliated Hospital of Nanchang University, Nanchang, China; 5 Hematology Department, The Education Affiliated Hospital, Sun Yat-Sen University, Guangdong, China; 6 People’s Hospital of Yongcheng City, Henan, China; 7 The Third People’s Hospital of Datong, Shanxi, China

**Keywords:** Clinical trials, Haematological cancer

## Abstract

Large granular lymphocytic leukemia (LGLL) is characterized by the clonal proliferation of cytotoxic T lymphocytes or NK cells. Standard first-line immunosuppressive treatments have limitations, achieving complete remission (CR) rates of up to 50%. Immune system dysregulation is implicated in LGLL. Promising results for thalidomide, an immunomodulatory drug, combined with prednisone and methotrexate (TPM), were observed in our pilot study. This multicenter study evaluated the efficacy and safety of a thalidomide, prednisone, and methotrexate (TPM) regimen in 52 symptomatic, methotrexate- and thalidomide-naive LGLL patients from June 2020 to August 2022. Thalidomide (100 mg daily for up to 24 months), prednisone (0.5–1.0 mg/kg every other day, tapered after 3 months), and methotrexate (10 mg/m^2^ weekly for up to 12 months) were administered. The primary objective was to determine the CR rate. The median follow-up duration was 29.0 months (range: 4.0–42.0). Forty-seven patients (90.4%) achieved hematological and symptomatic responses. Thirty-nine patients (75.0%) achieved CR. The median time to response was 3.0 months (range: 3.0–9.0). The median progression-free survival was 40.0 months (95% confidence interval (CI): 38.0–42.0), and the median duration of response was 39.0 months (95% CI: 36.1–41.9). The most common adverse event was peripheral neuropathy (24.1%), most of which (84.6%) were grades 1–2. Four patients experienced grade ≥3 adverse events. In conclusion, the TPM regimen was an effective and safe treatment for symptomatic LGLL patients, with a particularly high CR rate. This trial was registered at www.clinicaltrials.gov (#NCT04453345).

## Introduction

Large granular lymphocyte leukemia (LGLL) is a rare and heterogeneous group of chronic lymphoproliferative disorders characterized by the clonal expansion of cytotoxic lymphocytes, including T cells and natural killer (NK) cells.^1^ The prevalence of T-cell and NK-cell LGLL remains imprecisely defined, with estimates ranging from 2 to 5% of chronic lymphoproliferative diseases in North America and slightly higher rates of 5–6% in Asia. These lymphocytes, which normally play crucial roles in immune surveillance and defense against viral infections and malignancies, undergo dysregulation in LGLL, resulting in their pathological proliferation. The clinical spectrum of LGLL is remarkably diverse, ranging from asymptomatic cases identified incidentally to severe, symptomatic disease. Characteristic clinical manifestations include neutropenia, anemia, recurrent infections, transfusion dependence, and autoimmune phenomena such as rheumatoid arthritis (RA).^2^ These disease-associated complications frequently lead to significant impairment in both quality of life and overall survival, highlighting the critical need for effective therapeutic strategies in managing this complex disorder.

The management of symptomatic LGLL has historically centered on immunosuppressive therapy as the primary initial treatment approach. Current therapeutic approaches typically involve the use of methotrexate (MTX) at a dosage of 10 mg/m^2^ per week, cyclophosphamide (CTX) administered at 50–100 mg daily, or cyclosporine (CSA) given at 3–5 mg/kg per day. While these agents have established efficacy in symptom control and remain the foundation of first-line treatment, several critical limitations persist in the therapeutic landscape. Notably, the field lacks a standardized, evidence-based frontline treatment protocol supported by robust data from randomized controlled trials.^3^ The most recent prospective clinical trial specifically investigating LGLL was published in 2015,^4^ indicating a nearly decade-long gap in the development of treatment protocols grounded in high-quality clinical research. Meanwhile, these single-agents have only modest efficacy, with overall response rates (ORRs) ranging from 40 to 70% and complete remission (CR) rates up to 50%, in both retrospective reports and prospective clinical trials.^4–9^ A prospective clinical trial comparing the efficacy of MTX and CTX did not establish a preferred single-agent therapy as the frontline treatment of LGLL.^3^ The potential improvement in efficacy through the combination of immunosuppressive drugs remains uncertain. Prednisone (Pred) is frequently used as a combination immunosuppressive medication, particularly at the initiation treatment for patients with autoimmune disorders. Combined Pred and MTX is also often used for LGLL patients with severe neutropenia.^10^ Moreover, addition of Pred may not significantly increase treatment efficacy.^7^ These limitations underscore the critical need to reevaluate current treatment paradigms and develop novel strategies to improve the effectiveness of first-line therapies for LGLL.

LGLL is characterized by immune system dysregulation, with the JAK-STAT pathway playing a pivotal role in its pathogenesis by promoting leukemic cell survival, proliferation, and cytotoxic activity.^11^ A distinctive clinical feature of LGLL is its strong association with autoimmune disorders, as evidenced by concurrent RA diagnosis in approximately 15% of patients, along with frequent occurrences of Sjögren’s syndrome and autoimmune thyroid disorders.^12^ Meanwhile, dysregulated pro-inflammatory cytokines, particularly IL-15 and platelet-derived growth factor also suggests immune system dysfunction in LGLL.^13^ Additionally, B cell dysregulation manifests through hypergammaglobulinemia and atypical responses to anti-CD20 antibody therapy in LGLL patients with RA.^14^ Recent single-cell RNA sequencing has revealed that in T-LGLL, the non-leukemic T cell repertoire is more clonally restricted compared to other cancers and autoimmune disorders. These non-leukemic T cells establish connections with LGLL leukemia cells through costimulatory cell–cell interactions, pro-inflammatory cytokines secreted by monocytes, and IFNγ secreted by T-LGLL clones.^15^ Therefore, by delving into the immunological mechanisms of LGLL, we can design more effective and better-tolerated drug regimens and clinical treatment regimens. The immunomodulatory drug (IMiD) thalidomide (Thal) has demonstrated potential in the treatment of autoimmune disease^16^ and has also shown efficacy in peripheral T cell lymphomas and other hematologic tumors.^17,18^ MTX, a well-established anti-inflammatory and immunosuppressive agent, exerts its effects by suppressing the JAK/STAT pathway. Pred, as an adjunctive treatment, leads to lymphocyte downregulation and the rapid restoration of blood counts.^19^ The intriguing question of whether thalidomide, a IMiD combined with Pred and MTX (TPM), can effectively benefit LGLL treatment and potentially enhance the effectiveness of immunosuppressive drugs warrants further investigation.

Hence, we proactively developed a therapy for LGLL by introducing the triple combination of TPM. In our initial pilot study involving a cohort of 28 patients with a median follow-up of 26 months, we observed remarkably promising outcomes. The results were remarkably encouraging, demonstrating significant hematological improvements in 89% of patients (25/28), with particular emphasis on the normalization of peripheral blood counts. Notably, 75% of patients (21/28) achieved a CR.^20^ Importantly, the TPM regimen was well tolerated. Only one patient experienced grade 3 nausea. These compelling outcomes, coupled with the excellent tolerability profile, provided strong rationale for advancing this therapeutic strategy. Consequently, we have initiated a multicenter, randomized phase II clinical trial across 5 tertiary medical centers, aiming to rigorously evaluate the efficacy, safety, and long-term outcomes of TPM combination therapy.

## Results

### Patient characteristics

From June 2020 to August 2022, a total of 52 patients who were diagnosed with LGLL were enrolled in the trial (Supplementary Fig. 1). Among the 52 patients, 7 were diagnosed with NK-LGLL and 45 with T-LGLL, among which 37 cases were of the $\alpha \beta$ type and 8 cases were of the $\gamma \delta$ type. Among the 52 patients, 8 were diagnosed with severe anemia (<6 g/dL), 4 with granulocytopenia (<0.5 × 10^9^/L), and 2 with both severe anemia and granulocytopenia. Six patients in this study were diagnosed with concurrent autoimmune disorders. Two patients presented with RA, one with unclassified connective tissue disease, one with Hashimoto’s thyroiditis, and one with autoimmune hemolytic anemia. The baseline characteristics are summarized in Table 1. The severity of cytopenia at baseline for each patient, along with their transfusion dependency status, is presented in Table 1 and Supplementary Table 2.

**Table 1:** Demographic and baseline characteristics of enrolled patients

Characteristics	*N* = 52
Age (year)	
Median (range)	54 (39–72)
≥65, *n* (%)	5 (9.6)
Sex, male, *n* (%)	25 (48.1)
Classification, *n* (%)	
T-LGLL	45 (86.5)
αβ type	37 (71.2)
*γδ* type	8 (15.4)
NK-LGLL	7 (13.5)
Splenomegaly, *n* (%)	18 (38.3)
Hemoglobin level (g/L)	
Median (range)	73 (38–139)
≤110, *n* (%)	47 (90.4)
≤60, *n* (%)	10 (19.2)
Transfusion dependency, *n* (%)	12 (23.1%)
Platelet count (10^9^/L)	
Median (range)	227 (26–539)
≤100, *n* (%)	8 (15.4)
ANC (10^9^/L)	
Median (range)	1.4 (0.3–9.2)
<1.5, *n* (%)	26 (50.0)
<0.5, *n* (%)	6 (11.5)
LGL count (10^9^/L), median (range)	1.0 (0.1–12.3)
Bone marrow cellularity	
Hypercellular	35 (67.3)
Normocellular	15 (28.8)
Hypocellular	2 (3.8%)
PRCA, *n* (%)	12 (23.1)
STAT3 mutation, *n* (%)	22 (56.4)
Indications for treatment	
Anemia	24 (46.2)
Neutropenia	5 (9.6)
Thrombocytopenia	1 (1.9)
Cytopenia involving two or more cell lineages	22 (42.3)
Coexisting autoimmune disease	6 (11.5)
B symptoms	8 (15.4)

*LGLL* large granular lymphocytic leukemia, *NK-LGLL* NK-large granular lymphocytic leukemia, *ANC* absolute neutrophil count, *PRCA* pure red cell aplastic anemia

Among the 52 patients, 42 were newly diagnosed, whereas the remaining 10 had previously received at least one immunosuppressive therapy. The baseline characteristics of the two groups were comparable, as detailed in Supplementary Table 3. The front-line treatments for these 10 patients included CsA in six patients, CTX in one patient, and other cytotoxic drugs in three patients. The treatment outcomes varied, with three patients achieving CR, two achieving PR, and five showing stable disease (SD). PFS survival ranged from 4 to 100 months (Supplementary Table 4).

The most common reason for therapeutic intervention among the enrolled patients was anemia (46.2%), followed by a combination of anemia and neutropenia (42.3%). The primary hematological manifestation was anemia (90.4%), followed by neutropenia (50.0%) and thrombocytopenia (15.4%). Splenomegaly was observed in 18 patients (38.3%). Additionally, 23.1% of patients had pure red cell aplasia (PRCA), and 11.5% had autoimmune diseases. Among the patients, 39 underwent STAT3 mutation testing via next-generation sequencing (NGS), and the mutation rate was 56.4%.

### Efficacy

In total, 47 patients (90.4%, 95% confidence interval (CI), 82.4% to 98.4%) achieved hematological and symptomatic responses. Among them, 39 patients achieved CR (75.0%, 95% CI, 63.0% to 87.0%), and eight patients achieved PR (15.4%, 95% CI, 5.6% to 25.2%). Among the 12 patients who were transfusion-dependent at baseline, 10 patients (83.3%) achieved transfusion independence following treatment (Supplementary Table 2).

**Fig. 1:**
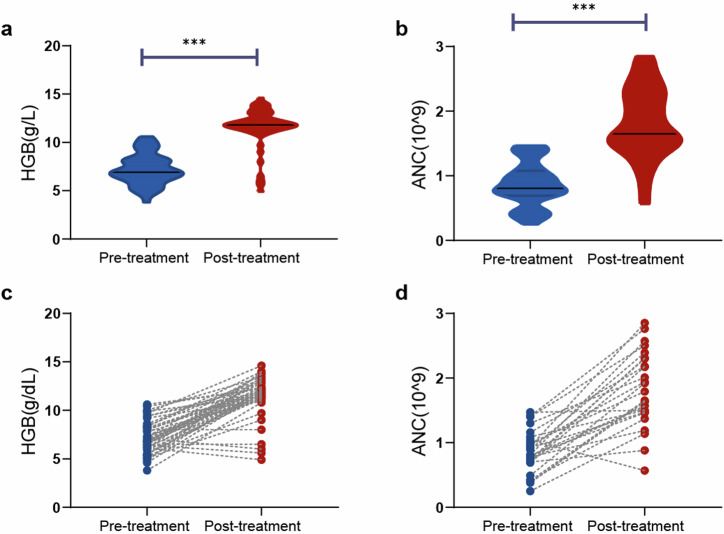
Changes of hemoglobin and absolute neutrophil counts before and after treatment. **a**, **b** Hemoglobin. **c**, **d** Absolute neutrophil counts; ***P < 0.001

When the 47 patients with anemia at baseline were evaluated, there was a significant increase in the median HGB level, from 6.9 g/dL (range: 3.8–10.6) to 11.8 g/dL (range: 4.9–14.6) (*P* < 0.001, Fig. 1a, b). Approximately 83.0% of patients achieved normal HGB levels (> 11 g/dL), with a median percentage increase from baseline of 71.0%. The median time to return to normal levels was 6.0 months (range: 3.0–36.0). Similarly, the median neutrophil count significantly increased, from 0.8 × 10^9^/L (range: 0.3–1.5) to 1.7 × 10^9^/L (range: 0.6–2.9) (*P* < 0.001, Fig. 1c, d). Approximately 73.1% (18/26) of patients achieved normal neutrophil counts ( > 1.5 × 10^9^/L), with a median time to return to the optimal or normal level of 6.0 months (range: 3.0–33.0). The results for newly diagnosed and previously treated patients are presented separately in Supplementary Fig. 2. Spleen size decreased or returned to normal in 70.0% of patients with splenomegaly. Importantly, this regimen demonstrated a good capacity to clear LGLL clones. Twenty-seven patients underwent posttreatment peripheral blood FCM and TCR gene rearrangement analysis, with three (3/27, 11.1%) patients achieving CMR.

The median follow-up time was 29.0 months (range: 4.0–42.0), and the median duration of the TPM regimen was 21.5 months (range: 3.0–42.0). Moreover, the median time to response was 3.0 months (range: 3.0–9.0), and the median time to CR was 6.0 months (range: 3.0–36.0).

**Fig. 2:**
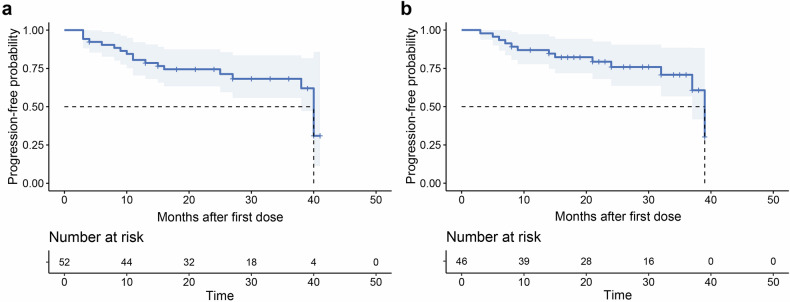
Survival curves of the enrolled patients. **a** Progression-free survival; **b** duration of response

No deaths were observed during the follow-up period. Eighteen patients (34.6%) experienced disease progression due to the relapse of cytopenias. The median PFS was 40.0 months (95% CI: 38.0–42.0), and the median DoR was 39.0 months (95% CI: 36.1–41.9) (Fig. 2a, b). The median OS was not reached. PFS and DoR data for newly diagnosed and previously treated patients are presented separately in Supplementary Fig. 3.

Moreover, the coronavirus disease 2019 (COVID-19) pandemic impacted the timely follow-up of some patients. Consequently, 17 patients continued using the Thal-based regimen for more than 2 years, exceeding the planned treatment duration. In order to evaluate the impact of the prolong using this regimen to our primary endpoint, we compared the CR rate between these 17 patients and the other 35 patients and found that there was no significant difference (88.2% vs. 68.6%, *P* = 0.232). The treatment response of these 14 patients with severe anemia and/or granulocytopenia was comparable to that of the other 38 patients (CR rate: 64.5% vs. 78.9%, *P* = 0.300; ORR: 100.0% vs. 86.8%, *P* = 0.307).

Among the 18 patients who experienced disease progression, 11 patients progressed during the treatment period, and seven patients progressed after discontinuation of treatment. Among the seven patients who discontinued treatment, five patients were retreated with the TPM regimen, and all of them achieved a treatment response, with three patients achieving CR and two patients achieving PR. Prior to retreatment, these patients discontinued treatment for varying durations, with a median duration of 5.0 months, ranging from 4.0 to 14.0 months. The subsequent treatment regimens and responses of the patients are summarized in Supplementary Table 5.

### Safety

Fifty-four patients who had received the TPM regimen for at least one month were included in the safety analysis set. During the study, a total of 35 AEs were reported for 27 patients (27/54, 50.0%), four of which (4/54, 7.4%) had serious adverse events (sAEs, grade ≥3) (Table 2). The most common AEs of any grade were peripheral neuropathy (13/54, 24.1%) and neutropenia (4/54, 7.4%). Among the 4 patients who developed neutropenia, 2 (3.7%) experienced associated infections, including tonsillitis and urinary tract infection, both of which were classified as grade 1–2 AEs. The most common grade 3–4 AE was peripheral neuropathy (2/54, 3.7%). Overall, the AEs occurred mainly during the first cycle (12/37, 32.4%). AEs led to the discontinuation of treatment in four patients (4/54, 7.4%).

**Table 2:** Summary of adverse events

Adverse events	Grade 1 or 2	Grade ≥ 3
	Number of patients (percent)	
Hematological toxicity		
Neutropenia	4 (7.4)	0
Nonhematological toxicity		
Neuropathy: sensory	9 (16.7)	2 (3.7)
Neuropathy: motor	2 (3.7)	0
Hyperglycemia	2 (3.7)	0
Obesity	1 (1.9)	0
Cushingoid	1 (1.9)	0
Constipation	3 (5.6)	0
Alanine/aspartate aminotransferase increased	2 (3.7)	0
Deep venous thromboembolism	1 (1.9)	0
Creatinine	0	1 (1.9)
Arthralgia	0	1 (1.9)
Osteoporosis	2 (3.7)	0
Photophobia	1 (1.9)	0
Fatigue	2 (3.7)	0
Erectile dysfunction	1 (1.9)	0

Among the 13 patients with peripheral neuropathy, symptoms completely resolved in 7 individuals following symptomatic treatment, dose reduction, or discontinuation of Thal. Additionally, symptoms were mitigated in 5 patients. Only one patient presented with grade 2 numbness in the extremities without improvement.

Thrombotic events were observed in one patient (1.9%, deep vein thrombosis of grade 2), a 68-year-old female with a Caprini score of 2, three months after TPM treatment. This thrombotic event was considered related to Thal. However, the patient was not concurrently taking aspirin for thrombosis prevention. After three months of anticoagulation therapy with rivaroxaban, the patient recovered without any sequelae. Additionally, we analyzed the cumulative dose of Pred and its related AEs. The median steroid dose was 1361 mg (505–2143 mg). Pred-related AEs were observed in 7 out of 52 (13.5%) patients, with none experiencing AEs of grade 3 or higher (Supplementary Table 7).

We focused on the AEs of those 17 patients who used the TPM regimen for more than 2 years. The incidence rates of both AEs and sAEs were similar between patients who used the regimen for more than 2 years and those who used it for less than 2 years (sAE: 0% vs. 5.7%, *P* = 1.000; AEs: 41.2% vs. 51.4%, *P* = 0.488).

### Exploratory translational analyses

In this study, 42 newly diagnosed patients and ten previously treated patients were enrolled. The ORR and CR rates were comparable between the newly diagnosed and previously treated patient groups (ORR: 90.5% vs. 90.0%, *P* = 1.000; CR rate: 73.8% vs. 80.0%, *P* = 1.000). The median follow-up times of the newly diagnosed and previously treated patient groups were 27 months and 30 months, respectively. Compared with previously treated patients, newly diagnosed patients exhibited similar PFS (median, 40.0 vs. 38.0 months; *P* = 0.116) and DoR (median, 39.0 vs. 37.0 months; *P* = 0.100; Supplementary Fig. 4a, b).

The eight patients with the γδ type exhibited comparable CR rate and ORR to the 37 patients with the αβ type (CR rate: 87.5% vs. 67.6%, *P* = 0.405; ORR: 87.5% vs. 88.9%, *P* = 1.000). Meanwhile, TPM regimen efficacy in the seven patients with NK-LGLL was similar to that in the 45 patients with T-LGLL (ORR: 100.0% vs. 88.9%, *P* = 1.000; CR rate: 85.7% vs. 73.3%, *P* = 0.815). The PFS and DoR rates were also comparable between the NK-LGLL and T-LGLL groups (PFS: median, not reached vs. 40.0 months, *P* = 0.489; DoR: median, not reached vs. 39.0 months, *P* = 0.607; Supplementary Fig. 4c, d).

We then analyzed the association between STAT3 mutation status and treatment efficacy. The mutational sites and variant allele frequencies of the STAT3 gene are shown in Supplementary Table 6. Patients with STAT3 mutations had CR rates and ORRs similar to those with wild-type STAT3 (CR rates: 63.6% vs. 88.2%, *P* = 0.169; ORR: 90.9% vs. 88.2%, *P* = 1.000). Compared with the wild-type group, the STAT3-mutated group had similar PFS and DoR (PFS: median, 38.0 months vs. not reached, *P* = 0.376; DoR: median, 37.0 months vs. not reached, *P* = 0.269; Supplementary Fig. 4e, f).

Moreover, the TPM regimen was also an effective option for patients with PRCA (*n* = 12). The CR rate (83.3% vs. 72.5%, *P* = 0.704) and ORR (91.7% vs. 90.0%, *P* = 1.000) were comparable between patients with and without PRCA. The PFS and DoR were also comparable between patients with and without PRCA (median PFS: 38.0 vs. 40.0 months, *P* = 0.293; median DoR: not 37.0 months vs. 39.0 months, *P* = 0.113; Supplementary Fig. 4g, h).

**Fig. 3:**
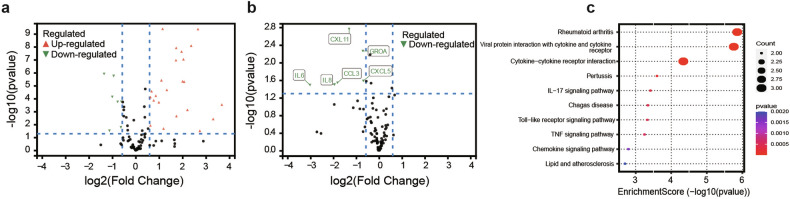
Serum cytokine profiling via the Olink multiplex proteomic platform. **a** Volcano plot of differentially expressed proteins between healthy donors and LGLL patients; **b** volcano plot of differentially expressed proteins of 17 paired patients before and after treatment; **c** Kyoto Encyclopedia of Genes and Genomes (KEGG) pathway enrichment analysis of significantly differentially expressed proteins

In this study, cytokine profiling, performed with the Olink® inflammation panel, revealed significant differences in the levels of inflammation-related cytokines between LGLL patients and healthy controls (HCs). Among the 92 cytokines tested, 30 were identified as differentially expressed proteins (DEPs) in LGLL patients, with 23 upregulated and 7 downregulated cytokines compared with HCs (Fig. 3a and Supplementary Table 8). The key cytokines with significantly elevated expression in LGLL patients included IL-8, CCL3, and CXCL5, which are known to play roles in immune and inflammatory processes. Paired samples were available for 17 patients before and after treatment. The expression of six cytokines (IL-6, IL-8, CCL3, CXCL5, CXCL11, and GROA) was notably downregulated after treatment (Fig. 3b and Supplementary Table 9). Pathway enrichment analysis revealed that the cytokine‒cytokine receptor interaction pathway was prominently enriched, suggesting a potential role of these pathways in disease pathophysiology and treatment response (Fig. 3c). We performed an additional analysis of cytokine changes before and after treatment in 10 patients with STAT3 mutations. Notably, we observed significant downregulation of CXCL11 and GROA2 expression following treatment (Supplementary Fig. 5 and Supplementary Table 10).

## Discussion

In recent decades, immunosuppressive drugs have been the main therapy for LGLL, but no new drugs or regimens have been developed. In this study, we first administered a Thal-based regimen for the treatment of LGLL, which was associated with excellent efficacy, with a CR rate of 75.0% and an ORR of 90.4%, the highest response rate reported to date. These findings provide us with a new strategy for treating LGLL, transitioning from immunosuppressive to immunomodulatory therapy.

The exact mechanism of action of Thal in the treatment of LGLL is not known. However, several properties of thalidomide could explain its effectiveness for LGLL. First, our analysis revealed differential expression of multiple cytokines compared with HCs, confirming the presence of immune alterations in LGLL patients (Fig. 3a). The cytokines with significant upregulation of expression, IL-8, CCL3, and CXL11, showed a substantial downregulation of expression after the TPM regimen (Fig. 3b). Second, Thal appears to reduce the activity of the pro-inflammatory cytokine TNF-α, which plays a key role in the pathogenesis of LGLL.^21^ However, TNF-α levels decreased after TPM treatment, although the difference was not statistically significant (Supplementary Table 8). Third, as an immunomodulator, Thal increases cell-mediated immunity by directly stimulating cytotoxic T cells and increasing the total number of lymphocytes, as well as the CD8+ and CD4 + T-cell counts.^22^ The above observations support the rationale and innovation behind Thal as a treatment for LGLL. The absence of myelosuppression and other significant adverse effects suggests that Thal could be an ideal agent for combination therapy with chemotherapy.

The potential mechanisms contributing to the additive therapeutic effect of Thal combined with MTX for the treatment of LGLL are also worth exploring. As a commonly used first-line therapy, MTX can inhibit key cytokines, such as TNF-α and IL-6, which are crucial to the activity of the JAK/STAT pathway, thereby suppressing its activation and exerting anti-inflammatory and immunosuppressive effects.^23^ Our analysis revealed a significant downregulation of IL-6 expression following TPM treatment (Fig. 3b), which suggests a possible synergistic benefit of Thal combined with MTX. On the other hand, MTX blocks the synthesis of purines and pyrimidines by inhibiting the activity of dihydrofolate reductase, which in turn inhibits DNA and RNA synthesis in cancer cells, preventing their proliferation.^24^ This mechanism might complement the antiproliferative effect of Thal.

Furthermore, high efficacy of the TPM regimen was observed in patients with PRCA, with an 83.3% CR rate and a 91.7% ORR. In contrast, a 30% ORR was reported for single-agent MTX in the treatment of LGLL and PRCA, which is lower than that for CTX (47%) and CsA (55%).^25^ The pathophysiology of PRCA in LGLL is thought to involve LGLs inhibiting erythropoiesis, chronic antigenic stimulation from infections or autoimmune diseases, and immune processes.^26^ Cytokines such as TNF released by LGLs may inhibit hematopoiesis and contribute to PRCA in LGLL. Additionally, PRCA in LGLL may result from autoantibody-dependent immune mechanisms involving immunoglobulins.^27^ The ability of MTX to reduce TNF-α levels and regulate the immune system may help explain the synergistic effect of the MTX-Thal combination in PRCA patients.

It is tempting to speculate that if most patients had mild to moderate cytopenia, this might have influenced the observed responses and reduced the comparability of the findings to those reported in the literature. However, in our study, the majority of patients (75.0%) achieved CR regardless of baseline cytopenia severity. Notably, 8 (Patients 1, 29, 33, 40, 41, 44, 45, and 52; Supplementary Table 2) of the 9 patients who achieved PR had moderate to severe anemia or severe neutropenia before treatment. Moreover, the treatment response of these 14 patients with severe anemia and/or granulocytopenia was similar to that of the other 38 patients. Future studies with larger cohorts, including those with more severe cytopenias, are needed to confirm these findings.

In general, the TPM regimen was well tolerated with manageable toxicity, although neuropathy was common. In a study by *Loughran*,^4^ 55 patients with LGLL were treated with MTX, and 13 patients (23.6%) experienced ≥grade 3 AEs, which was higher than the 8.9% rate of sAEs observed in this study. Although the two studies are not directly comparable due to differences in the monitoring and reporting of AEs, our findings suggest that TPM therapy may not significantly increase the risk of AEs, especially severe AEs. The potential risks related to long-term Thal therapy should be considered with caution. Maintenance therapy is a proven strategy for sustaining remission in indolent lymphomas and other hematological tumors, especially indolent lymphoma.^28,29^ Thal has also been used effectively in regimens for conditions such as primary amyloidosis,^30^ Langerhans cell histiocytosis,^31^ and Castleman disease,^32^ typically with a 2-year duration of maintenance therapy, similar to our approach. Despite known side effects, the safety profile of prolonged Thal use remained acceptable in our study, with no significant differences in AEs between patients treated for more than 2 years and those treated for less. These findings suggest that long-term Thal use is safe when monitored carefully, but further trials are needed to optimize the duration of maintenance therapy. In this trial, the use of prednisone was carefully optimized to balance therapeutic efficacy and minimize side effects. As an adjunct treatment, prednisone facilitated rapid lymphocyte downregulation and blood count recovery, though it did not sustain long-term responses or improve overall response rates.^19^ Administered at 0.5–1.0 mg/kg every other day with a gradual taper of 5–10 mg per week after three months, this approach minimized steroid-related adverse effects, which occurred in only 13.5% of patients, with no grade 3 or higher events.

Refractory LGLL can lead to transfusion-dependent anemia and severe neutropenia, but treatment options remain limited due to the rarity of the disease and lack of prospective data. In this study, 10 patients with relapsed or refractory LGLL were enrolled, with an ORR of 90.0% and a CR rate of 80.0%. These results offer a promising option for patients with relapsed or refractory LGLL and demonstrate the high efficacy of this regimen across various clinical contexts of LGLL. Moreover, we also observed that some patients experienced disease progression after discontinuation but the TPM regimen was still effective. Thus, the time-limited use of Thal-based regimens should be explored in the future, similar to the use of BTK inhibitors in the treatment of chronic lymphocytic leukemia.

Our study has several limitations. First, the median age of our cohort was relatively young, at 54 years. This demographic factor may have contributed to the low frequency of AEs observed. Future studies with more age-diverse populations are needed to confirm the efficacy and safety of the regimen for older individuals. Second, the short minimum follow-up period of 4 months and the median follow-up of 29.0 months were sufficient to assess the response of the regimen. However, longer follow-up durations and larger cohort studies are necessary to confirm the sustained efficacy of TPM and further optimize the treatment strategy. Third, the differences in PFS and DoR observed between previously treated and newly diagnosed patients did not reach statistical significance, likely due to the limited sample size of the previously treated group, which reduced the statistical power. Nonetheless, these findings underscore the robust effectiveness of the TPM regimen across diverse patient populations. Future studies with larger cohorts are essential to validate these promising results and to better elucidate the long-term outcomes of previously treated patients.

In summary, our study has established early evidence supporting a novel Thalidomide-based regimen for LGLL, with greater efficacy than current single immunosuppressive agents. Good tolerability of the TPM regimen was demonstrated. To rigorously validate the efficacy of the TPM regimen compared with other regimens, a randomized controlled phase III trial is necessary.

## Materials and methods

### Participants

We enrolled patients who met the diagnostic criteria of T-LGLL or NK-LGLL according to the World Health Organization (WHO) classification.^33^ Patients aged 18 years or older who were untreated or who exhibited refractory or relapsed disease after non-MTX and non-Thal-based therapy were included in this study. Moreover, all enrolled patients had indications for treatment to relieve symptoms or peripheral blood cytopenia, such as severe neutropenia, neutropenia with recurrent infections, symptomatic or transfusion-dependent anemia, severe thrombocytopenia, or associated autoimmune conditions requiring therapy. The complete eligibility criteria are detailed in the approved protocol, which is provided in the Supplementary Appendix.

The study protocol was approved by the institutional review boards or independent ethics committees at each participating site. The trial was conducted in accordance with the principles outlined in the Declaration of Helsinki, Good Clinical Practice guidelines, and applicable local laws. All enrolled patients provided written informed consent. This trial was registered at www.clinicaltrials.gov with the trial number #NCT04453345.

### Trial design and treatment

This was an investigator-initiated, prospective, open-label, multicenter, phase II study conducted at six sites in China. Continuous oral administration of the treatment was required, with each cycle lasting three months. Patients initiated treatment with Thal at a dosage of 50 mg per night during the first week, with a potential increase to 100 mg per night for those who tolerated the drug. Pred was prescribed at a dosage of 0.5–1.0 mg/kg every other day, and MTX was administered at a dosage of 10 mg/m^2^ per week.^15^ Aspirin (0.1 g per day) was administered along with Thal to prevent thrombosis. The Pred dose was gradually tapered after three months. For patients who achieved a CR after one or two cycles, MTX could be discontinued after one cycle of consolidation, with a maximum duration of four cycles. For patients who achieved partial remission (PR) or CR, Thal maintenance therapy was recommended for a maximum duration of 2 years in total, but the duration also depended on patients’ or investigators’ choices. Additional information about the study design is available in the Supplementary Appendix.

### Assessments and endpoints

Baseline assessments included physical examination, Eastern Cooperative Oncology Group (ECOG) performance status assessment, and laboratory and imaging tests necessary to diagnose LGLL (complete blood count, blood smear examination, bone marrow biopsy with immunohistochemistry, flow cytometry (FCM), TCR gene rearrangement analysis, STAT3 mutation testing, Vβ repertoire analysis, KIR phenotyping, and abdominal color ultrasound).

Treatment response was assessed by the investigators. The efficacy assessment criteria were based on those of the ECOG 5998 T-LGLL trial^3^: CR was defined as absolute neutrophil count (ANC) > 1.5 × 10^9^/L, hemoglobin (HGB) level >11 g/dL, and platelet count (PLT) > 100 × 10^9^/L; hematological PR was defined as an improvement in the blood ANC > 0.5 × 10^9^/L, an increase in the HGB level of >1 g/dL, a PLT > 50 × 10^9^/L and the absence of transfusion requirement. Progressive disease (PD) was defined as a worsening of hematological parameters (a decrease in the HGB level of 2 g/dL or a HGB level less than 10 g/dL, a decrease in the ANC of 0.5 × 10^9^/L or an ANC less than 1.0 × 10^9^/L, a decrease in the PLT of 20 × 10^9^/L in PLT and a PLT less than 100 × 10^9^/L, or transfusion requirement) or findings of organomegaly, such as hepatosplenomegaly, are detected in patients previously achieving PR/CR. PD was confirmed after at least two assessments, with an interval of more than 4 weeks. No response (NR) was defined as a lack of CR/PR or PD. A complete molecular response (CMR) was defined as the absence of clonal T-cell detection by TCR gene rearrangement or by FCM and meeting the criteria for hematological complete response. Adverse events (AEs) were graded by the investigator according to the Common Terminology Criteria for Adverse Events, version 5.0.

The primary endpoint of this trial was the CR rate. The secondary endpoints were safety, ORR, progression-free survival (PFS), duration of response (DoR), and overall survival (OS). Details regarding disease assessments, safety outcomes, management of AEs, and exploratory analysis methods are provided in the Supplementary Appendix.

### Cytokine profiling

The Olink® target 92 inflammation panel (Olink Proteomics, LC-Bio Technology Co., Ltd. BGI-Shenzhen, China) was used to quantify protein levels. Differential cytokine expression was calculated from the normalized protein expression units (NPX) with the Mann‒Whitney test and corrected with the Benjamini‒Hochberg procedure. The cytokines included in the panel are shown in Supplementary Table 1.

### Statistical analysis

The sample size for this study was determined on the basis of the primary endpoints with a two-sided type I error of 5% and a type II error of 0.2 for the hypothesis being tested. A retrospective study of 45 patients who received CTX monotherapy was used as a historical control,^5^ and an ORR of 71% and a CR rate of 47% were reported, which is the highest reported CR rate for immunosuppressive drug treatment in a large cohort.^4^ To calculate the sample size, the researchers considered a minimum clinical significance criterion of a CR rate of 47% as the historical control and 70% as the expected CR. On the basis of these criteria, a total of 35 patients in this study group would be sufficient to demonstrate a true CR rate that is better than that of the historical control. Additionally, a dropout rate of 20% was accounted for; therefore, a total enrollment of at least 42 patients was required for the study to achieve its objectives.

In this study, efficacy was assessed after one cycle of therapy, and patients who had received the regimen for more than three months were considered evaluable for efficacy. All patients who had received at least one month of the regimen were included in the safety analyses. The associations between categorical variables were assessed using Pearson’s chi-square test and Fisher’s exact test. Comparisons of continuous variables were evaluated using Student’s *t* test. OS and PFS were calculated from the date of treatment, and the DoR was calculated from the first documented response. For PFS and DoR analyses, death or disease progression were considered events.

Analyses were performed with SPSS 22 (SPSS, Inc., Chicago, IL). Survival curves were constructed using the Kaplan–Meier method, and differences were estimated through the log-rank test. A two-sided *P* value less than 0.05 was considered statistically significant.

## Supplementary information

Supplementary Table 11

Supplementary materials

Protocol

## Data Availability

The study protocol is available in the Supplementary Appendix. The normalized protein expression units (NPX) for the 92 inflammatory proteins in both patients and healthy donors are presented in Supplementary Table 11.
