# Supplementary material for: Thalidomide-based regimen shows promising efficacy in large granular lymphocytic leukemia: a multicenter phase II study
Source: Signal Transduct Target Ther. 2025 Mar 12;10:85. doi: 10.1038/s41392-025-02164-4 (PMC11897152; doi:10.1038/s41392-025-02164-4)
Supplement: Supplementary file 2 — Supplementary materials [file 41392_2025_2164_MOESM2_ESM.docx]

Supplementary Materials for

Thalidomide-Based Regimen Shows Promising Efficacy in Large Granular Lymphocytic Leukemia: A Multicenter Phase II Study

Ying Yu, Yuxi Li, Rui Cui, Yuting Yan, Fei Li, Yan Chen, Tingyu Wang, Xiaoli Hu, Yaqing Feng, Tengteng Yu, Yanshan Huang, Jingwen Sun, Rui Lyu, Wenjie Xiong, Qi Wang, Wei Liu, Gang An, Weiwei Sui, Yan Xu, Wenyang Huang, Dehui Zou, Huijun Wang, Zhijian Xiao, Jianxiang Wang, Lugui Qiu, and Shuhua Yi.

Correspondence to: yishuhua@ihcams.ac.cn

**This file includes:**

List of Principal Investigators

Study Inclusion and Exclusion Criteria

Statistical Considerations

Figures. S1 to S5

Tables S1 to S10

Appendix

References

List of Principal Investigators

| **Study Site, Country** | **Lead** |
| --- | --- |
| Institute of Hematology and Blood Diseases Hospital, Chinese Academy of Medical Sciences, China | Shuhua Yi/Lugui Qiu |
| The First Affiliated Hospital of Nanchang University, China | Fei Li |
| The Eighth Affiliated Hospital, Sun Yat-sen University, China | Yan Chen |
| The People's Hospital of Yongcheng, China | Xiaoli Hu |
| The Third People's Hospital of Datong, China | Yaqing Feng |

Study Inclusion and Exclusion Criteria

**Inclusion criteria**

1. Patients who fulfill the diagnostic criteria for T-LGLL or chronic lymphoproliferative disorders of nature killer cells (NK-LGLL) specified in the 2022 World Health Organization (WHO) Classification of Lymphoid Neoplasms ^[1]^

2. Patients are fully informed about the study and willing to participate in the study and provide written informed consent form (ICF)

3. Male or female, aged ≥18 years

4. Treatment-naive patients, or patients treated with or relapsed from a non-methotrexate/ thalidomide-based regimen

5. Patients with at least one of the following indications for LGLL treatment:

a. absolute neutrophil count (ANC) <0.5 × 109/L, or neutropenia with recurrent infections

b. Hemoglobin <100 g/L, or requiring infusion of red blood cells

c. Platelets level <50 × 109/L

d. Concomitant autoimmune diseases requiring treatment

e. Symptomatic splenomegaly

f. Severe B symptoms (fever of unknown cause with body temperature over 38 °C; night sweats; weight loss of ≥10% within half a year)

g. Pulmonary hypertension

6. Patients with Eastern Cooperative Oncology Group (ECOG) score of 0 to 2 (Appendix 1)

7. Patients with life expectancy ≥6 months.

**Exclusion criteria**

1. Patients not diagnosed with T-LGLL or NK-LGLL

2. Patients with no indication for LGLL treatment

3. Patients who are unable to understand or follow study procedures

4. Patients who have been diagnosed or treated for malignancies other than LGLL within the past five years

5. Patients with non-lymphoma-related hepatic and renal impairment: alanine aminotransferase (ALT) > 3 × upper limit of normal (ULN), aspartate aminotransferase (AST) > 3 × ULN, total bilirubin > 2 × ULN, and serum creatinine clearance (CrCl) <30 mL/min

6. Patients with other severe diseases that have an impact on this study (uncontrolled diabetes, stomach ulcers, other severe cardiopulmonary conditions, etc.), at the discretion of investigators.

7. High-risk patients based on the Caprini score for thromboembolism (Appendix 2)

8. Patients with a known history of HIV infection, or with active hepatitis B virus (HBV) infection, or any uncontrolled active systemic infection requiring intravenous antibiotics

Notes: HBV infection is considered active if all following criteria are met: a. HBV DNA quantification ≥2000 IU/mL; b. ALT ≥2 times × ULN; c. LGLL, medications, and other causes-induced hepatitis are excluded. Patients with active HBV infection at the time of initial diagnosis who are converted to inactive HBV infection after adequate anti-HBV treatment could be enrolled in this study.

9. Patients who have undergone major surgery (excluding lymph node biopsy) within the past 14 days or who are expected to undergo major surgery during study treatment

10. Pregnant or lactating women, and women of childbearing potential who have not taken contraceptive measures

**Statistical Considerations**

**Sample size:**

A retrospective study of 45 patients is used as the historical control, in which cyclophosphamide monotherapy was applied with an effective rate of 72% and CRR of 47%^1^. From the perspective of clinical expertise, taking the CRR of 70% as the minimum threshold for clinical significance, the sample size of 35 patients in this group is deemed sufficient to test the overall true CRR that is superior to that of the historical control at a false positive rate α = 0.05 (two sides) and false negative rate β = 0.2 (ie, power = 80%). With a 20% dropout rate, at least 42 patients would be required to enroll in this study.

**Populations for analyses:**

*Screened population*: All participants who signed the informed consent form (ICF).

*Safety Analysis*: Safety analyses included all patients who had received at least one month of the regimen.

*Efficacy Analysis*: Efficacy was assessed after one cycle of therapy, and patients who had received the regimen for more than 3 months were considered evaluable for efficacy.

**Primary Endpoint:**

• CRR of patients with symptomatic LGLL treated with thalidomide, prednisone, and methotrexate.

**Secondary Endpoints:**

• Response rates: ORR and PR rate (time frame: 24 months after the last treatment)

Point estimates of rates will be calculated for each protocol analysis set. An estimate of the 95% CI for the response rate will also be derived. Graphical and descriptive analyses will be used to explore associations between relevant markers and responses.

• PFS and OS (time frame: 24 months after the last treatment)

Time from the first day to PD or death from any reason. The median duration of the overall response will be assessed with a 95% CI.

• DoR (time frame: 24 months after the last treatment)

The time when CR or PR is met until the first date of recurrent or progressive disease. The median duration of the overall response will be assessed with a 95% CI.

• Effect of biomarkers on efficacy and adverse reactions

**Safety Analysis:**

The safety profile will be analyzed based on AEs, physical examination, vital sign measurements, laboratory measurements, and ECG findings. AEs will be graded as per NCI CTCAE (Version 5.0).

In general, the safety analysis will be descriptive and presented in tabular form with appropriate summary statistics. The number of patients and events of hematological and non-hematological toxicity (based on NCI CTCAE Version 5.0) will be listed, and the incidence will be calculated.

**
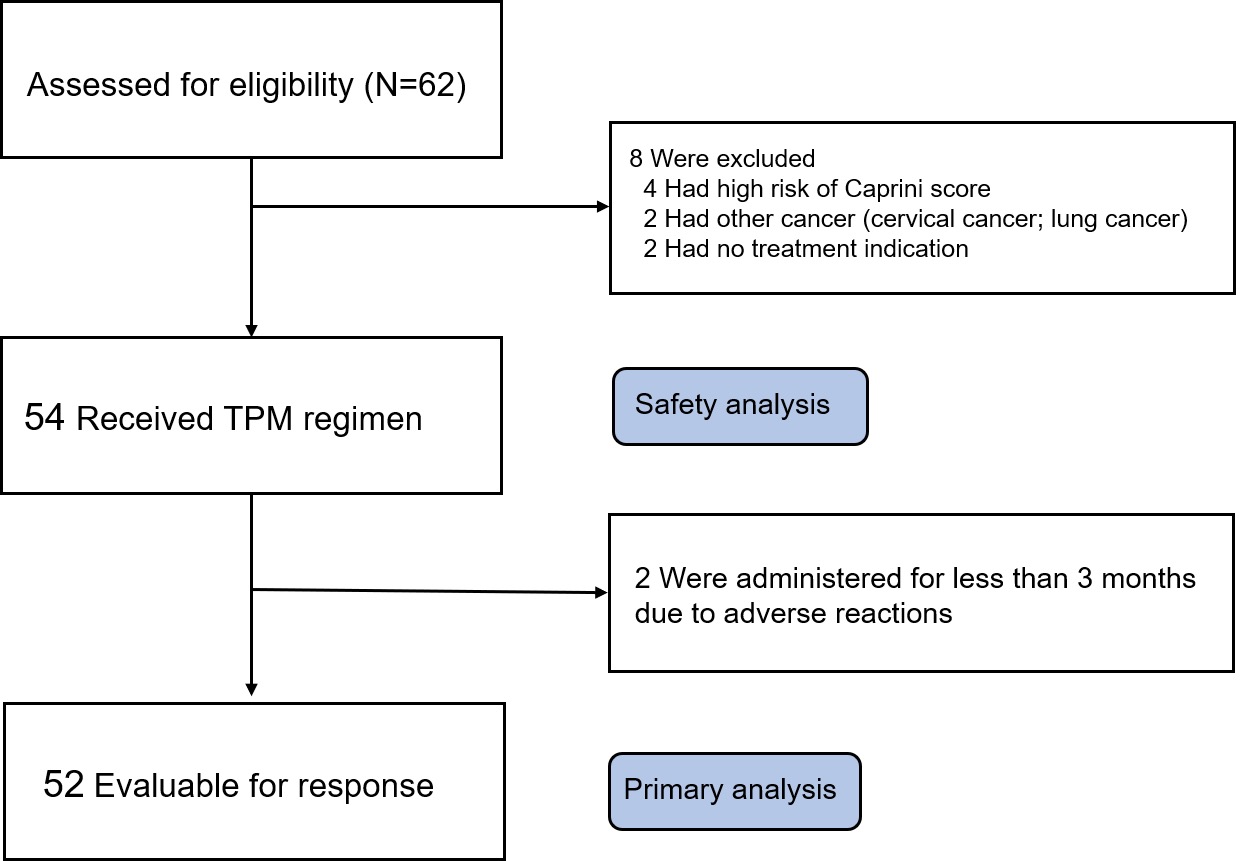
**

Figure. S1.

Flow chart of enrolled patient selection.


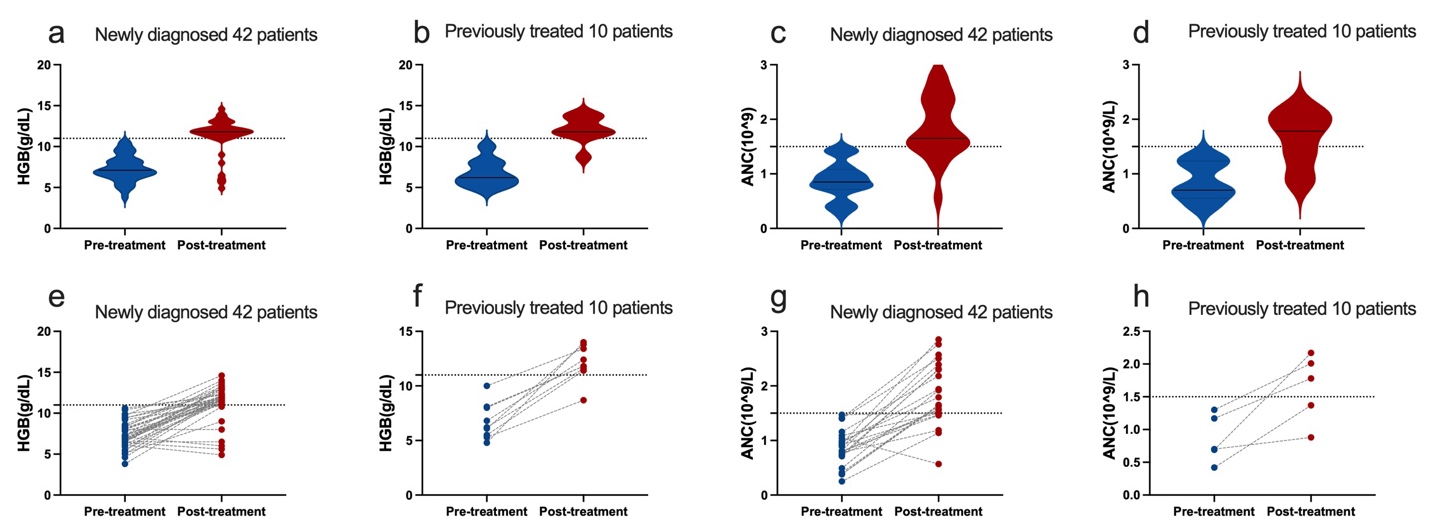


Figure. S2.

Changes in hemoglobin levels and absolute neutrophil counts after treatment. (a, e) Hemoglobin level for 42 newly diagnosed patients. (b, f) Hemoglobin level for 42 newly diagnosed patients. (c, g) Absolute neutrophil counts for 10 previously treated patients. (d, h) Absolute neutrophil counts for 10 previously treated patients.


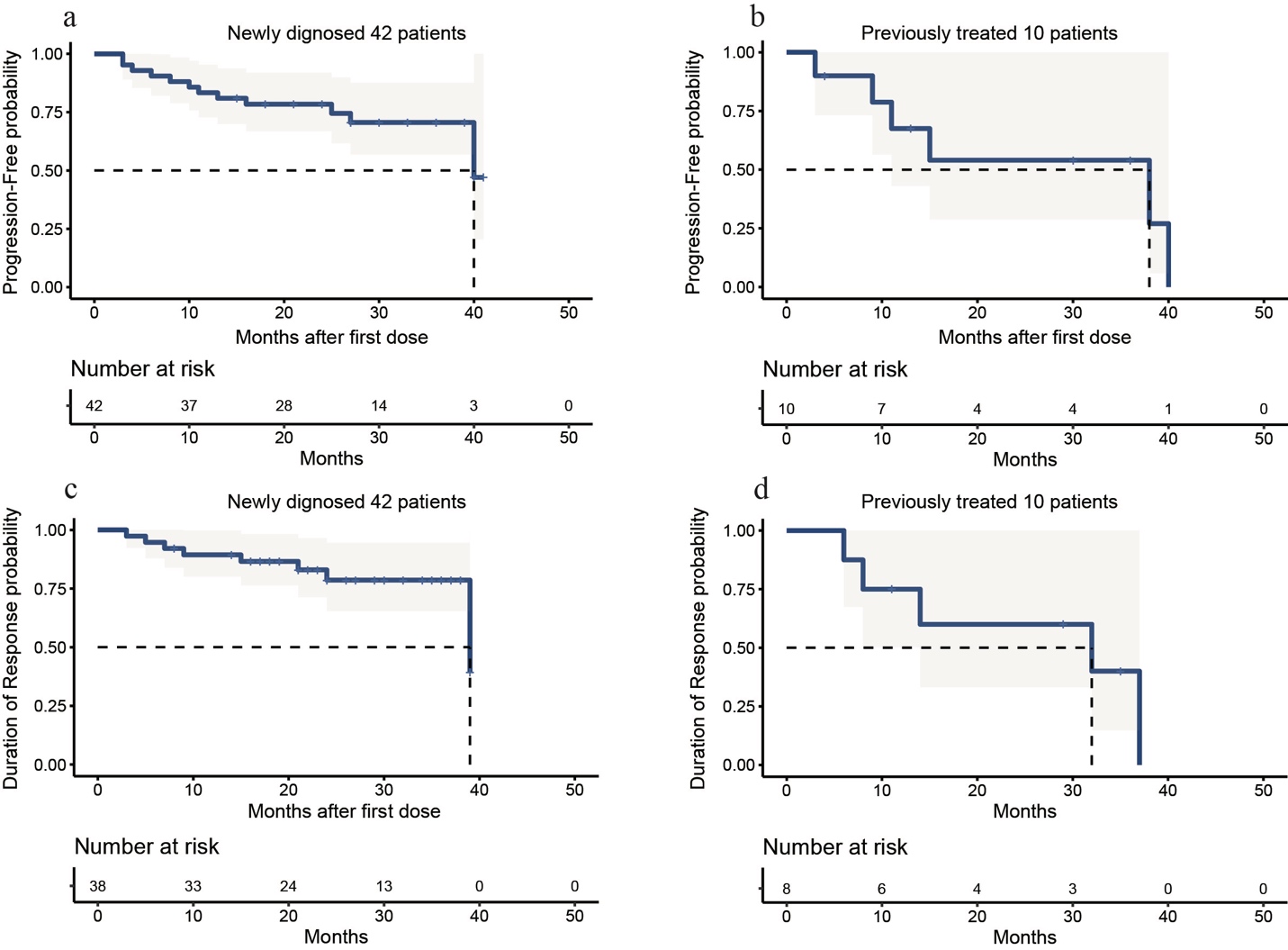


Figure. S3.

Survival curves of the enrolled patients. (a) Progression-free survival for 42 newly diagnosed patients; (b) Progression-free survival for 10 previously treated patients; (c) Duration of response for 42 newly diagnosed patients; (d) Duration of response for 10 previously treated patients.


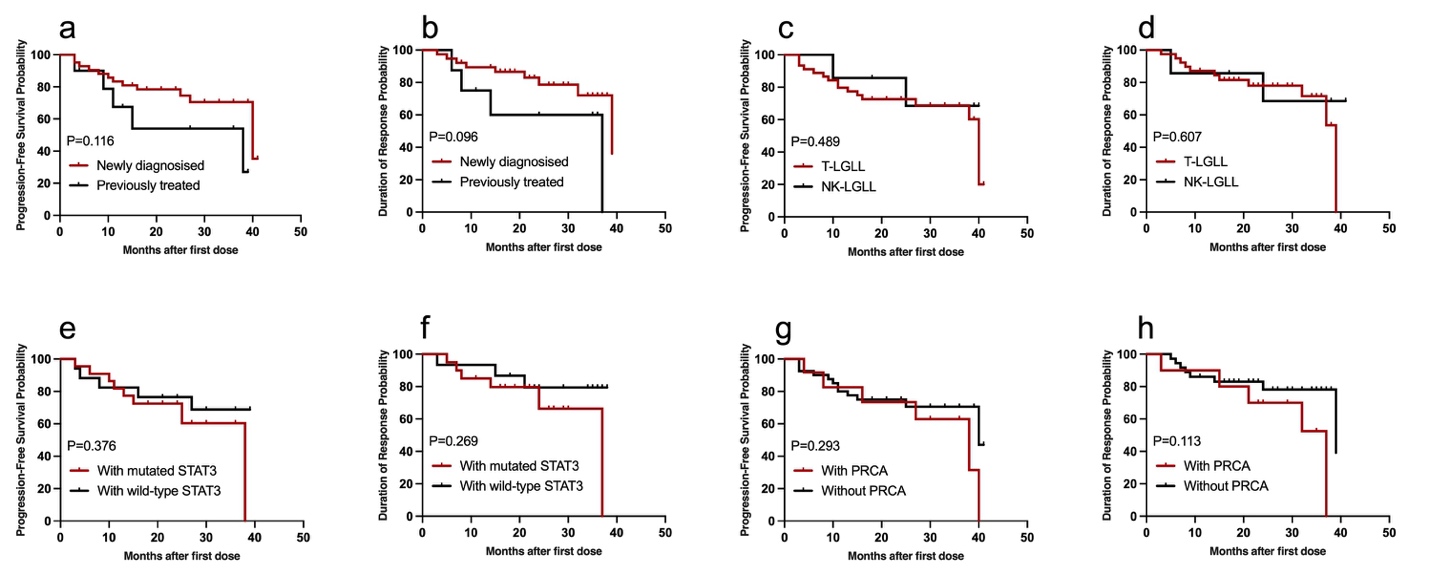


**Figure. S4.**

Survival curves of enrolled patients. (a-b) Progression-free survival and duration of response of newly diagnosed and ten previously treated patients; (c-d) Progression-free survival and duration of response of T-cell LGLL and NK cell LGLL; (e-f) Progression-free survival and duration of response of patients with mutated and wild-type STAT3; (g-h) Progression-free survival and duration of response of patients with and without pure red cell aplasia.


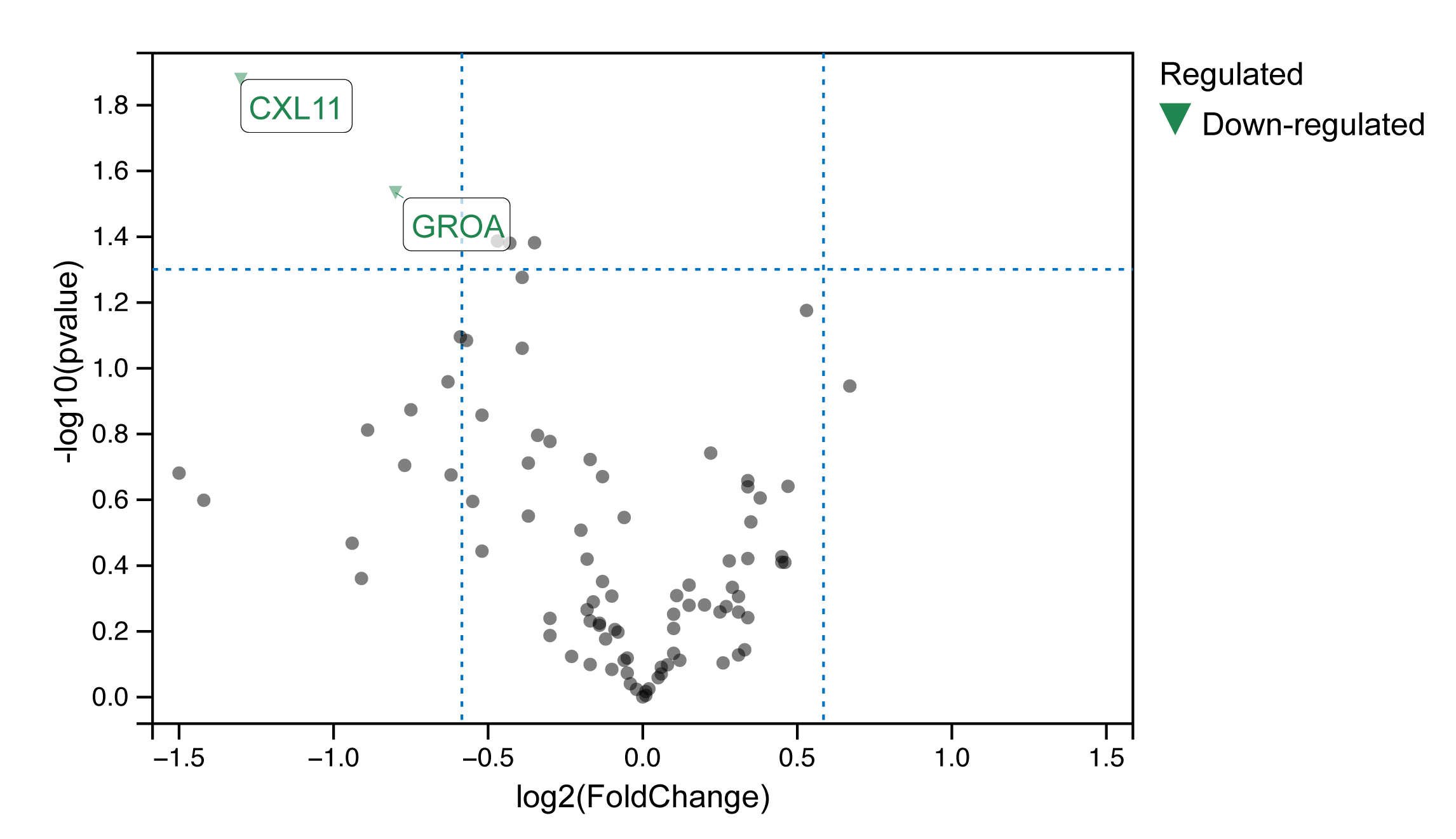


**Figure. S5.**

Volcano plot of differentially expressed proteins in patients with STAT3 mutations before and after treatment.

Table S1.

List of 92 inflammation cytokines panel

| Adenosine Deaminase (ADA) | P00813 |
| --- | --- |
| Artemin (ARTN) | Q5T4W7 |
| Axin-1 (AXIN1) | O15169 |
| Beta-nerve growth factor (Beta-NGF) | P01138 |
| Caspase-8 (CASP-8) | Q14790 |
| C-C motif chemokine 19 (CCL19) | Q99731 |
| C-C motif chemokine 20 (CCL20) | P78556 |
| C-C motif chemokine 23 (CCL23) | P55773 |
| C-C motif chemokine 25 (CCL25) | O15444 |
| C-C motif chemokine 28 (CCL28) | Q9NRJ3 |
| C-C motif chemokine 3 (CCL3) | P10147 |
| C-C motif chemokine 4 (CCL4) | P13236 |
| CD40L receptor (CD40) | P25942 |
| CUB domain-containing protein 1 (CDCP1) | Q9H5V8 |
| C-X-C motif chemokine 1 (CXCL1) | P09341 |
| C-X-C motif chemokine 10 (CXCL10 ) | P02778 |
| C-X-C motif chemokine 11 (CXCL11) | O14625 |
| C-X-C motif chemokine 5 (CXCL5 ) | P42830 |
| C-X-C motif chemokine 6 (CXCL6) | P80162 |
| C-X-C motif chemokine 9 (CXCL9 ) | Q07325 |
| Cystatin D (CST5) | P28325 |
| Delta and Notch-like epidermal growth factor-related receptor (DNER) | Q8NFT8 |
| Eotaxin (CCL11) | P51671 |
| Eukaryotic translation initiation factor 4E-binding protein 1 (4E-BP1) | Q13541 |
| Fibroblast growth factor 19 (FGF-19) | O95750 |
| Fibroblast growth factor 21 (FGF-21) | Q9NSA1 |
| Fibroblast growth factor 23 (FGF-23) | Q9GZV9 |
| Fibroblast growth factor 5 (FGF-5) | P12034 |
| Fms-related tyrosine kinase 3 ligand (Flt3L) | P49771 |
| Fractalkine (CX3CL1 ) | P78423 |
| Glial cell line-derive neurotrophic factor (GDNF) | P39905 |
| Hepatocyte growth factor (HGF) | P14210 |
| Interferon gamma (IFN-gamma) | P01579 |
| Interleukin-1 alpha (IL-1 alpha) | P01583 |
| Interleukin-10 (IL10) | P22301 |
| Interleukin-10 receptor subunit alpha (IL-10RA) | Q13651 |
| Interleukin-10 receptor subunit beta (IL-10RB) | Q08334 |
| Interleukin-12 subunit beta (IL-12B) | P29460 |
| Interleukin-13 (IL-13) | P35225 |
| Interleukin-15 receptor subunit alpha (IL-15RA) | Q13261 |
| Interleukin-17A (IL-17A) | Q16552 |
| Interleukin-17C (IL-17C) | Q9P0M4 |
| Interleukin-18 (IL-18) | Q14116 |
| Interleukin-18 receptor 1 (IL-18R1) | Q13478 |
| Interleukin-2 (IL-2) | P60568 |
| Interleukin-2 receptor subunit beta (IL-2RB) | P14784 |
| Interleukin-20 (IL-20) | Q9NYY1 |
| Interleukin-20 receptor subunit alpha (IL-20RA) | Q9UHF4 |
| Interleukin-22 receptor subunit alpha-1 (IL-22 RA1) | Q8N6P7 |
| Interleukin-24 (IL-24) | Q13007 |
| Interleukin-33 (IL-33) | O95760 |
| Interleukin-4 (IL-4) | P05112 |
| Interleukin-5 (IL5) | P05113 |
| Interleukin-6 (IL6) | P05231 |
| Interleukin-7 (IL-7) | P13232 |
| Interleukin-8 (IL-8) | P10145 |
| Latency-associated peptide transforming growth factor beta-1 (LAP TGF-beta-1) | P01137 |
| Leukemia inhibitory factor (LIF) | P15018 |
| Leukemia inhibitory factor receptor (LIF-R) | P42702 |
| Macrophage colony-stimulating factor 1 (CSF-1) | P09603 |
| Matrix metalloproteinase-1 (MMP-1) | P03956 |
| Matrix metalloproteinase-10 (MMP-10) | P09238 |
| Monocyte chemotactic protein 1 (MCP-1) | P13500 |
| Monocyte chemotactic protein 2 (MCP-2) | P80075 |
| Monocyte chemotactic protein 3 (MCP-3) | P80098 |
| Monocyte chemotactic protein 4 (MCP-4) | Q99616 |
| Natural killer cell receptor 2B4 (CD244) | Q9BZW8 |
| Neurotrophin-3 (NT-3) | P20783 |
| Neurturin (NRTN) | Q99748 |
| Oncostatin-M (OSM) | P13725 |
| Osteoprotegerin (OPG) | O00300 |
| Programmed cell death 1 ligand 1 (PD-L1) | Q9NZQ7 |
| Protein S100-A12 (EN-RAGE) | P80511 |
| Signaling lymphocytic activation molecule (SLAMF1) | Q13291 |
| SIR2-like protein 2 (SIRT2) | Q8IXJ6 |
| STAM-binding protein (STAMPB) | O95630 |
| Stem cell factor (SCF) | P21583 |
| Sulfotransferase 1A1 (ST1A1) | P50225 |
| T cell surface glycoprotein CD6 isoform (CD6) | P30203 |
| T-cell surface glycoprotein CD5 (CD5) | P06127 |
| T-cell surface glycoprotein CD8 alpha chain (CD8A) | P01732 |
| Thymic stromal lymphopoietin (TSLP) | Q969D9 |
| TNF-beta (TNFB) | P01374 |
| TNF-related activation-induced cytokine (TRANCE) | O14788 |
| TNF-related apoptosis-inducing ligand (TRAIL) | P50591 |
| Transforming growth factor alpha (TGF-alpha) | P01135 |
| Tumor necrosis factor (Ligand) superfamily, member 12 (TWEAK) | O43508 |
| Tumor necrosis factor (TNF) | P01375 |
| Tumor necrosis factor ligand superfamily member 14 (TNFSF14) | O43557 |
| Tumor necrosis factor receptor superfamily member 9 (TNFRSF9) | Q07011 |
| Urokinase-type plasminogen activator (uPA) | P00749 |
| Vascular endothelial growth factor A (VEGF-A) | P15692 |

Table S2.

Baseline cytopenia and transfusion dependency data

| Patients | Indications for TPM regimen | Hemoglobin level (g/dL) | Platelet Count (x10^9/L) | Neutrophil Count (x10^9/L) | Transfusion Dependency | Transfusion intensity | Transfusion Independence after treatment |
| --- | --- | --- | --- | --- | --- | --- | --- |
| Pt 1 | 4 | 5.3 | 254 | 0.7 | Yes | Twice a month | Yes |
| Pt 2 | 1 | 6.2 | 506 | 4.19 | No | - | - |
| Pt 3 | 4 | 6.7 | 226 | 0.74 | No | - | - |
| Pt 4 | 4 | 9.7 | 79 | 0.94 | No | - | - |
| Pt 5 | 1 | 8.3 | 265 | 1.67 | No | - | - |
| Pt 6 | 2 | 11.0 | 209 | 0.81 | No | - | - |
| Pt 7 | 1 | 8.1 | 358 | 9.15 | No | - | - |
| Pt 8 | 1 | 9.4 | 185 | 4.4 | No | - | - |
| Pt 9 | 1 | 8.0 | 281 | 3.11 | No | - |  |
| Pt 10 | 1 | 5.9 | 193 | 2.74 | No | - | - |
| Pt 11 | 1 | 8.2 | 306 | 3.63 | No | - | - |
| Pt 12 | 1 | 10.4 | 115 | 2.54 | No | - | - |
| Pt 13 | 1 | 13.1 | 178 | 1.54 | No | - | - |
| Pt 14 | 4 | 7.3 | 275 | 1.4 | No | - | - |
| Pt 15 | 4 | 8.6 | 88 | 0.8 | No | - | - |
| Pt 16 | 1 | 6.9 | 216 | 1.61 | No | - | - |
| Pt 17 | 1 | 4.6 | 193 | 1.5 | Yes | Three times a month | Yes |
| Pt 18 | 4 | 8.4 | 171 | 0.38 | No | - | - |
| Pt 19 | 4 | 6.2 | 255 | 0.74 | No | - | - |
| Pt 20 | 4 | 6.1 | 138 | 0.69 | Yes | Once a month | Yes |
| Pt 21 | 4 | 9.2 | 240 | 1.09 | No | - | - |
| Pt 22 | 1 | 4.8 | 298 | 4.78 | Yes | Three times a month | Yes |
| Pt 23 | 1 | 7.3 | 307 | 2.09 | No | - | - |
| Pt 24 | 1 | 6.8 | 539 | 2.68 | No | - | - |
| Pt 25 | 1 | 6.9 | 274 | 1.58 | No | - | - |
| Pt 26 | 2 | 10.0 | 56 | 1.3 | No | - | - |
| Pt 27 | 4 | 8.0 | 262 | 1.16 | No | - | - |
| Pt 28 | 4 | 8.1 | 88 | 2.58 | No | - | - |
| Pt 29 | 2 | 12.9 | 163 | 0.25 | No | - | - |
| Pt 30 | 1 | 5.0 | 620 | 1.97 | Yes | Twice a month | Yes |
| Pt 31 | 1 | 6.5 | 120 | 2.89 | No | - | - |
| Pt 32 | 1 | 3.8 | 279 | 7.68 | Yes | Four times a month | Yes |
| Pt 33 | 4 | 7.8 | 95 | 0.92 | No | - |  |
| Pt 34 | 4 | 7.1 | 113 | 0.78 | No | - |  |
| Pt 35 | 4 | 6.8 | 113 | 0.89 | No | - |  |
| Pt 36 | 1 | 7.1 | 227 | 1.51 | No | - |  |
| Pt 37 | 1 | 6.5 | 376 | 2.18 | Yes | Once a month |  |
| Pt 38 | 3 | 13.9 | 26 | 4.94 | No | - | No |
| Pt 39 | 2 | 10.6 | 266 | 0.41 | No | - | - |
| Pt 40 | 4 | 7.3 | 284 | 0.96 | No | - | - |
| Pt 41 | 4 | 6.8 | 385 | 1.41 | No | - | - |
| Pt 42 | 1 | 9.9 | 278 | 1.86 | No | - | - |
| Pt 43 | 4 | 6.8 | 58 | 0.8 | No | - | - |
| Pt 44 | 4 | 8.0 | 157 | 0.38 | No | - | - |
| Pt 45 | 4 | 5.1 | 406 | 1.04 | Yes | Twice a month | Yes |
| Pt 46 | 4 | 5.5 | 87 | 0.49 | Yes | Once a month | Yes |
| Pt 47 | 1 | 7.3 | 140 | 1.75 | No | - | - |
| Pt 48 | 1 | 5.4 | 259 | 1.84 | Yes | Twice a month | Yes |
| Pt 49 | 4 | 6.4 | 226 | 1.41 | Yes | Once a month | No |
| Pt 50 | 2 | 12.6 | 260 | 1.13 | No | - | - |
| Pt 51 | 1 | 6.1 | 286 | 1.52 | No | - | - |
| Pt 52 | 4 | 5.5 | 139 | 0.42 | No | - | - |

Table S3.

Demographic and baseline characteristics of newly diagnosed and previously treated patients

| Characteristics | Newly diagnosed patients (N=42) | Previously treated patients (N=10) | *P* |
| --- | --- | --- | --- |
| Age (year) |  |  |  |
| Median (range) | 53 (39-72) | 53 (38-63) | 0.124 |
| ≥65, n (%) | 5 (11.9) | 0 | 0.569 |
| Gender, male, n (%) | 19 (45.2) | 6 (60.0) | 0.626 |
| Classification, n (%) |  |  | 0.383 |
| T-LGL | 35 (83.5) | 10 (100.0) | - |
| NK-LGLL | 7 (16.7) | 0 | - |
| Splenomegaly, n (%) | 17 (45.9) | 1(10.0) | 0.088 |
| Hemoglobin level (g/L) |  |  |  |
| Median (range) | 73 (38-139) | 62 (48-100) | 0.145 |
| ≤110, n (%) | 37 (88.1) | 10 (100.0) | 0.569 |
| Platelet count (10^9^/L) |  |  |  |
| Median (range) | 226 (26-209) | 268 (56-539) | 0.740 |
| ≤100, n (%) | 8 (19.0) | 1 (10.0) | 0.830 |
| ANC (10^9^/L) |  |  |  |
| Median (range) | 1.4 (0.3-9.2) | 2.1 (0.4-4.8) | 0.485 |
| ＜1.5, n (%) | 23 (54.8) | 3 (30.0) | 0.159 |
| PRCA, n (%) | 9 (21.4) | 3 (30.0) | 0.872 |
| STAT3 mutation, n (%) | 17 (53.1) | 5 (71.4) | 0.643 |
| The regimens of prior therapies |  |  |  |
| CsA | - | 6 (60.0) | - |
| Cytotoxic drugs | - | 3 (30.0) | - |
| CTX | - | 1 (10.0) | - |
| Adverse events | 22 (52.4) | 4 (40.0) | 0.482 |
| Grade 1-2 | 20 (47.6) | 4 (40.0) | 0.935 |
| Grade≥3 | 2 (4.8) | 0 (10.0) | 1.000 |

CsA, cyclosporin A; Cytotoxic drugs including chlorambucil, fludarabine with cyclophosphamide, cyclophosphamide+ vincristine+ prednisone; CTX, cyclophosphamide.

Table S4.

The regimens and outcomes of patients previously treated with front-line therapy

| **Patients** | **Number of treatment lines before TPM** | **Front-line regimen** | | **Efficacy** | **Duration of the regimen (months)** | **PFS (months)** | **Indications for TPM regimen**^a^ |
| --- | --- | --- | --- | --- | --- | --- | --- |
| Pt 1 | 1 | CsA | | SD | 9 | 9 | 4 |
| Pt 2 | 1 | CsA | | SD | 10 | 10 | 1 |
| Pt 9 | 1 | Chlorambucil | | CR | 18 | 100 | 1 |
| Pt 20 | 1 | Vindesine+CTX+Pred | | SD | 4 | 4 | 4 |
| Pt 22 | 1 | Fludarabine + CTX | | CR | 3 | 70 | 1 |
| Pt 24 | 1 | CTX | | SD | 4 | 4 | 1 |
| Pt 26 | 1 | CsA | | PR | 24 | 42 | 2 |
| Pt 28 | 2 | CsA; CsA+Pred | CR; PR | | 6; 10 | 12; 10 | 4 |
| Pt 51 | 1 | CsA | PR | | 8 | 10 | 1 |
| Pt 52 | 1 | CsA | SD | | 4 | 4 | 4 |

PR, partial remission; CR, complete response; SD, stable disease; CTX, cyclophosphamide; CsA, cyclosporin A; Pred, prednisone

^a^Indications: 1-Anemia, 2- neutropenia, 3- thrombocytopenia, 4-cytopenia involving two or more cell lineages.

Table S5.

The subsequent treatment regimen and response of patients with poor response or disease progression

| **Patients** | **Duration of TPM regimen (months)** | **Treatment response of TPM regimen** | **Subsequent treatment regimen** | **Treatment response of Subsequent regimen** |
| --- | --- | --- | --- | --- |
| Pt 1 | 43 | PR | CTX | PR |
| Pt 2 | 40 | CR | TPM | PR |
| Pt 4 | 32 | CR | TPM | CR |
| Pt 7 | 22 | CR | TPM | CR |
| Pt 9 | 15 | CR | Danazol | NA |
| Pt 11 | 6 | CR | CsA | PR |
| Pt 15 | 27 | CR | CTX | PR |
| Pt 21 | 28 | PR | Thal | PR |
| Pt 26 | 6 | CR | TPM | CR |
| Pt 27 | 4 | SD | NA | NA |
| Pt 28 | 24 | CR | NA | NA |
| Pt 29 | 13 | PR | CsA | SD |
| Pt 33 | 5 | PR | TPM | PR |
| Pt 34 | 4 | SD | CsA | PR |
| Pt 37 | 3 | SD | CTX | CR |
| Pt 38 | 20 | CR | Thal | SD |
| Pt 39 | 3 | SD | CsA | PR |
| Pt 51 | 4 | SD | CsA | SD |

PR, partial remission; CR, complete responses; SD, stable disease; CTX, cyclophosphamide; CsA, cyclosporin A; TPM, thalidomide plus prednisone and methotrexate; NA, not available.

Table S6.

STAT3 gene mutation sites and variant allele frequencies in patients

| Patients | Mutation site | Variant allele frequencies |
| --- | --- | --- |
| Pt 2 | p.Y640F | 28.7% |
| Pt 9 | p.S614R | 3.0% |
| Pt 15 | p.S614R | 32.9% |
| Pt 18 | p.D661Y | 7.2% |
| Pt 19 | p.E166D | 32.0% |
| Pt 27 | p.P715L | 8.2% |
| Pt 28 | p.E625_D627delinsDVL | 2.8% |
| Pt 29 | p.D661Y | 9.2% |
| Pt 30 | p.S614R、p.D661Y | 2.5%、0.52% |
| Pt 33 | p.Y640F | 18.2% |
| Pt 36 | p.N647I | 5.3% |
| Pt 39 | p.Y640F | 19.5% |
| Pt 40 | p.S614R | 5.0% |
| Pt 41 | p.K658R、p.D661Y | 2.3%、1.8% |
| Pt 42 | p.Y640F | 1.0% |
| Pt 44 | p.N647I、p.D661Y | 0.7%、0.45% |
| Pt 45 | p.D661Y | 12.2% |
| Pt 46 | p.Y640F | 9.4% |
| Pt 47 | p.Y640F | 4.1% |
| Pt 48 | p.Y640F | 8.7% |
| Pt 51 | p.H410R | 1.1% |
| Pt 52 | p.G618R、p.E638Q | 1.1%、3.9% |

Table S7.

Summary of the administration of prednisone and relevant adverse events

| Patients | Treatment duration (weeks) | Cumulative dose (mg) | Adverse effects |
| --- | --- | --- | --- |
| Pt 1 | 19 | 1718 | - |
| Pt 2 | 17 | 1450 | - |
| Pt 3 | 17 | 1555 | - |
| Pt 4 | 8 | 505 | - |
| Pt 5 | 21 | 1059 | Osteoporosis  grade 2 |
| Pt 6 | 14 | 1178 | - |
| Pt 7 | 19 | 1615 | - |
| Pt 8 | 15 | 1328 | Photophobia  grade 2 |
| Pt 9 | 28 | 1910 | - |
| Pt 10 | 22 | 1875 | - |
| Pt 11 | 20 | 953 | - |
| Pt 12 | 20 | 1695 | - |
| Pt 13 | 14 | 803 | - |
| Pt 14 | 18 | 763 | - |
| Pt 15 | 20 | 1638 | - |
| Pt 16 | 21 | 1563 | - |
| Pt 17 | 19 | 1645 | - |
| Pt 18 | 18 | 1525 | - |
| Pt 19 | 23 | 1355 | - |
| Pt 20 | 15 | 1345 | Cushingoid  grade 2 |
| Pt 21 | 21 | 2143 | - |
| Pt 22 | 18 | 1278 | - |
| Pt 23 | 17 | 1158 | - |
| Pt 24 | 20 | 1223 | - |
| Pt 25 | 22 | 1698 | - |
| Pt 26 | 17 | 1003 | - |
| Pt 27 | 17 | 1376 | - |
| Pt 28 | 14 | 905 | - |
| Pt 29 | 24 | 1576 | - |
| Pt 30 | 15 | 1313 | - |
| Pt 31 | 10 | 523 | - |
| Pt 32 | 20 | 1695 | - |
| Pt 33 | 20 | 1725 | - |
| Pt 34 | 18 | 1293 | - |
| Pt 35 | 17 | 1043 | - |
| Pt 36 | 17 | 1548 | Hyperglycemia  grade 2 |
| Pt 37 | 13 | 1218 | - |
| Pt 38 | 13 | 623 | - |
| Pt 39 | 25 | 1968 | - |
| Pt 40 | 26 | 1471 | Hyperglycemia  grade 2 |
| Pt 41 | 13 | 1060 | - |
| Pt 42 | 18 | 1425 | - |
| Pt 43 | 16 | 1390 | - |
| Pt 44 | 17 | 1555 | - |
| Pt 45 | 16 | 973 | - |
| Pt 46 | 18 | 1008 | - |
| Pt 47 | 13 | 970 | - |
| Pt 48 | 18 | 1643 | Obesity  grade 1 |
| Pt 49 | 14 | 1299 | - |
| Pt 50 | 25 | 1166 | Osteoporosis  grade 2 |
| Pt 51 | 16 | 1473 | - |
| Pt 52 | 19 | 1368 | - |

Table S8.

List of differential cytokines between healthy donors and LGLL patients

| Entry Name | log2FC | adj_pvalue | Class |
| --- | --- | --- | --- |
| PD1L1 | 1.14 | 3.89E-10 | Up |
| CXL11 | 2.65 | 4.10E-10 | Up |
| CD6 | 2.36 | 7.82E-09 | Up |
| TNR9 | 1.72 | 1.16E-08 | Up |
| CXCL9 | 1.95 | 2.22E-08 | Up |
| IL18 | 2.03 | 8.75E-08 | Up |
| CD244 | 1.71 | 1.04E-07 | Up |
| TGFA | -1.36 | 1.19E-06 | Down |
| ST1A1 | -0.95 | 1.70E-06 | Down |
| IL12B | 1.09 | 3.63E-06 | Up |
| IFNG | 1.99 | 4.44E-06 | Up |
| IL10 | 2.06 | 4.72E-06 | Up |
| CSF1 | 0.40 | 1.73E-05 | Non |
| CD8A | 1.32 | 2.04E-05 | Up |
| CD5 | 0.67 | 2.59E-05 | Up |
| CCL23 | 0.85 | 5.73E-05 | Up |
| AXIN1 | -1.01 | 7.06E-05 | Down |
| I15RA | 0.62 | 7.46E-05 | Up |
| GROA | 0.76 | 0.000131688 | Up |
| HGF | -0.62 | 0.000166425 | Down |
| TNF14 | -0.78 | 0.000170005 | Down |
| DNER | -0.56 | 0.000176076 | Non |
| CDCP1 | 0.98 | 0.000184089 | Up |
| CCL3 | 3.67 | 0.000279761 | Up |
| LIFR | 0.36 | 0.000401263 | Non |
| STABP | -0.51 | 0.00042093 | Non |
| CCL4 | 2.20 | 0.000712936 | Up |
| CCL11 | -0.52 | 0.000714382 | Non |
| CASP8 | 1.73 | 0.002146029 | Up |
| SCF | -0.52 | 0.002176649 | Non |
| CXL10 | 0.38 | 0.002654528 | Non |
| TNF12 | -0.41 | 0.002716452 | Non |
| IL8 | 3.30 | 0.003618908 | Up |
| NGF | -0.09 | 0.004440244 | Non |
| CCL28 | 0.62 | 0.011384546 | Up |
| CCL8 | -0.36 | 0.012035193 | Non |
| S10AC | -0.64 | 0.015827342 | Down |
| FGF21 | 1.18 | 0.019761314 | Up |
| VEGFA | 0.52 | 0.020062472 | Non |
| NTF3 | -0.35 | 0.026382934 | Non |
| IL18R | 0.27 | 0.028541088 | Non |
| FGF19 | -1.13 | 0.029279342 | Down |
| TNFA | 2.73 | 0.029571552 | Up |
| SLAF1 | 0.40 | 0.050161301 | Non |
| CXCL6 | 0.37 | 0.05873361 | Non |
| TNR5 | 0.24 | 0.060249503 | Non |
| TNF11 | -0.29 | 0.085582716 | Non |
| NRTN | 0.38 | 0.093025666 | Non |
| MMP10 | -0.55 | 0.100150698 | Non |
| IL6 | 5.81 | 0.100313314 | Non |
| TR11B | -0.24 | 0.135624823 | Non |
| MMP1 | 0.23 | 0.135827368 | Non |
| I10R1 | 1.64 | 0.146198417 | Non |
| IL17C | -0.53 | 0.159219821 | Non |
| ADA | 0.30 | 0.160805393 | Non |
| CYTD | -0.30 | 0.16097995 | Non |
| IL5 | 1.10 | 0.177257958 | Non |
| SIR2 | -0.34 | 0.181301492 | Non |
| FGF23 | 0.85 | 0.184321117 | Non |
| CCL7 | 2.88 | 0.186088327 | Non |
| TNF10 | 0.12 | 0.195503766 | Non |
| I10R2 | 0.17 | 0.218757208 | Non |
| IL2RB | 0.26 | 0.234951884 | Non |
| IL1A | 1.66 | 0.287499383 | Non |
| I20RA | -0.65 | 0.289316904 | Non |
| IL2 | -0.13 | 0.346486389 | Non |
| IL17 | -0.61 | 0.351250069 | Non |
| IL4 | -1.50 | 0.358983797 | Non |
| IL20 | 0.59 | 0.38524045 | Non |
| CCL2 | -0.14 | 0.392590246 | Non |
| CCL20 | 0.76 | 0.399581636 | Non |
| FGF5 | 0.21 | 0.408007199 | Non |
| X3CL1 | 0.12 | 0.476524694 | Non |
| IL13 | 1.65 | 0.478775676 | Non |
| IL33 | -0.09 | 0.512769085 | Non |
| ARTN | -0.21 | 0.53285516 | Non |
| TSLP | -0.09 | 0.53542215 | Non |
| IL24 | -0.16 | 0.565004051 | Non |
| CCL13 | -0.12 | 0.573934666 | Non |
| ONCM | -0.18 | 0.609563316 | Non |
| FLT3L | 0.13 | 0.617517197 | Non |
| LIF | 0.06 | 0.649245851 | Non |
| CCL25 | 0.10 | 0.664627927 | Non |
| CCL19 | 0.20 | 0.687503686 | Non |
| IL7 | -0.07 | 0.695132243 | Non |
| 4EBP1 | 0.18 | 0.710047481 | Non |
| I22R1 | 0.11 | 0.741301305 | Non |
| CXCL5 | 0.08 | 0.748980458 | Non |
| UROK | -0.02 | 0.818728325 | Non |
| GDNF | -0.03 | 0.859047831 | Non |
| TNFB | 0.04 | 0.87480408 | Non |
| TGFB1 | -0.01 | 0.949714601 | Non |

Table S9.

List of differential cytokines of 17 paired patients before and after treatment

| Entry Name | log2FC | adj_pvalue | Class |
| --- | --- | --- | --- |
| TR11B | 0.05 | 0.697534188 | None |
| CXL11 | -1.32 | 0.001701841 | Down |
| TNF11 | 0.27 | 0.318542052 | None |
| AXIN1 | 0.31 | 0.296498056 | None |
| CCL25 | -0.09 | 0.662439491 | None |
| TNF12 | 0.01 | 0.962056962 | None |
| TNF14 | -0.06 | 0.829936167 | None |
| STABP | 0.10 | 0.662580936 | None |
| FGF19 | 0.15 | 0.575763176 | None |
| IL33 | 0.02 | 0.888721909 | None |
| UROK | -0.06 | 0.637000996 | None |
| ADA | -0.48 | 0.178701281 | None |
| TGFA | 0.21 | 0.470290674 | None |
| TGFB1 | -0.11 | 0.515194144 | None |
| NGF | -0.06 | 0.173768748 | None |
| TNFB | -0.11 | 0.569230075 | None |
| TNFA | -0.63 | 0.2854679 | None |
| IFNG | -0.06 | 0.921049372 | None |
| IL1A | -0.35 | 0.227464068 | None |
| CD8A | -0.20 | 0.593088176 | None |
| CXL10 | -0.11 | 0.407997727 | None |
| MMP1 | 0.08 | 0.662629487 | None |
| IL4 | 0.32 | 0.460408781 | None |
| IL5 | -0.71 | 0.365342931 | None |
| IL6 | -3.03 | 0.031820274 | Down |
| CD5 | -0.11 | 0.587926257 | None |
| MMP10 | -0.12 | 0.594893434 | None |
| GROA | -0.72 | 0.005383782 | Down |
| CSF1 | -0.13 | 0.117232385 | None |
| IL8 | -1.96 | 0.031096601 | Down |
| CCL3 | -1.83 | 0.028290629 | Down |
| FGF5 | 0.13 | 0.281512082 | None |
| IL7 | 0.08 | 0.761644176 | None |
| CCL4 | -0.95 | 0.07292515 | None |
| CCL2 | -0.09 | 0.67329794 | None |
| ONCM | -0.37 | 0.398405156 | None |
| HGF | 0.31 | 0.110514038 | None |
| IL2RB | 0.01 | 0.91458961 | None |
| LIF | 0.10 | 0.521564383 | None |
| VEGFA | -0.34 | 0.228910114 | None |
| NTF3 | -0.15 | 0.340691729 | None |
| SCF | 0.04 | 0.801181122 | None |
| IL10 | -0.27 | 0.59993436 | None |
| TNR5 | -0.36 | 0.028941146 | None |
| CYTD | 0.28 | 0.148526121 | None |
| IL12B | 0.37 | 0.257272608 | None |
| CD6 | -0.32 | 0.42595725 | None |
| IL13 | -2.55 | 0.401748298 | None |
| GDNF | 0.42 | 0.058838295 | None |
| LIFR | -0.26 | 0.062362144 | None |
| CXCL5 | -0.70 | 0.025633454 | Down |
| FLT3L | -0.40 | 0.006506029 | None |
| ST1A1 | 0.55 | 0.037382625 | None |
| TNF10 | -0.11 | 0.400818997 | None |
| CCL11 | -0.01 | 0.941421391 | None |
| CCL23 | -0.18 | 0.309001519 | None |
| IL2 | 0.06 | 0.617082161 | None |
| X3CL1 | -0.20 | 0.354698217 | None |
| CCL20 | -0.69 | 0.112641825 | None |
| CCL8 | 0.67 | 0.05386203 | None |
| CCL7 | -2.73 | 0.370771132 | None |
| CXCL6 | -0.57 | 0.026085562 | None |
| S10AC | 0.18 | 0.667425916 | None |
| TNR9 | -0.39 | 0.170788657 | None |
| CXCL9 | -0.20 | 0.47704315 | None |
| I10R2 | -0.11 | 0.559361426 | None |
| IL24 | 0.21 | 0.386247863 | None |
| I15RA | -0.28 | 0.108313898 | None |
| SLAF1 | 0.24 | 0.435215707 | None |
| IL18R | -0.08 | 0.60766459 | None |
| 4EBP1 | -0.33 | 0.63482032 | None |
| I10R1 | 0.62 | 0.435972934 | None |
| IL18 | -0.37 | 0.212846823 | None |
| CASP8 | -1.14 | 0.104837307 | None |
| IL17 | 0.28 | 0.264091699 | None |
| ARTN | 0.13 | 0.615991603 | None |
| SIR2 | 0.10 | 0.76835946 | None |
| I22R1 | -0.02 | 0.932959031 | None |
| DNER | 0.19 | 0.121818557 | None |
| TSLP | 0.02 | 0.905002724 | None |
| CCL13 | 0.09 | 0.764889434 | None |
| CCL19 | 0.07 | 0.880067701 | None |
| NRTN | 0.27 | 0.557567029 | None |
| CD244 | -0.54 | 0.189223156 | None |
| FGF23 | -1.97 | 0.088191497 | None |
| CDCP1 | 0.10 | 0.699139039 | None |
| CCL28 | -0.06 | 0.830772246 | None |
| FGF21 | -0.85 | 0.11099636 | None |
| IL20 | -0.04 | 0.970838963 | None |
| PD1L1 | -0.31 | 0.08285459 | None |
| IL17C | 0.01 | 0.968213669 | None |
| I20RA | 0.11 | 0.633515885 | None |

Table S10.

List of differential cytokines of 17 paired patients before and after treatment

| Entry Name | log2FC | adj_pvalue | Class |
| --- | --- | --- | --- |
| TR11B | 0.10 | 0.5595766 | None |
| CXL11 | -1.30 | 0.01323724 | Down |
| TNF11 | 0.20 | 0.52446558 | None |
| AXIN1 | 0.27 | 0.53035619 | None |
| CCL25 | -0.18 | 0.54220255 | None |
| TNF12 | -0.05 | 0.76043309 | None |
| TNF14 | -0.04 | 0.91051416 | None |
| STABP | 0.15 | 0.45639431 | None |
| FGF19 | 0.35 | 0.2932771 | None |
| IL33 | 0.01 | 0.96171475 | None |
| UROK | -0.08 | 0.63422426 | None |
| ADA | -0.55 | 0.25407921 | None |
| TGFA | 0.25 | 0.55077008 | None |
| TGFB1 | -0.14 | 0.5958709 | None |
| NGF | -0.06 | 0.28426338 | None |
| TNFB | -0.12 | 0.6655642 | None |
| TNFA | 0.31 | 0.74427622 | None |
| IFNG | 0.33 | 0.71784277 | None |
| IL1A | -0.13 | 0.44505258 | None |
| CD8A | -0.30 | 0.5760728 | None |
| CXL10 | -0.09 | 0.62286583 | None |
| MMP1 | 0.10 | 0.61819274 | None |
| IL4 | 0.46 | 0.38935041 | None |
| IL5 | -0.94 | 0.34039178 | None |
| IL6 | -1.42 | 0.2519491 | None |
| CD5 | -0.16 | 0.51318606 | None |
| MMP10 | -0.05 | 0.84485717 | None |
| GROA | -0.80 | 0.02921674 | Down |
| CSF1 | -0.13 | 0.21351572 | None |
| IL8 | -1.80 | 0.17649465 | None |
| CCL3 | -0.91 | 0.43567485 | None |
| FGF5 | 0.11 | 0.49121916 | None |
| IL7 | 0.06 | 0.84980831 | None |
| CCL4 | -0.30 | 0.64967599 | None |
| CCL2 | -0.17 | 0.58642082 | None |
| ONCM | -0.17 | 0.7955589 | None |
| HGF | 0.34 | 0.22933128 | None |
| IL2RB | -0.10 | 0.49286048 | None |
| LIF | 0.15 | 0.52566824 | None |
| VEGFA | -0.37 | 0.281436 | None |
| NTF3 | -0.30 | 0.16691538 | None |
| SCF | 0.06 | 0.81125871 | None |
| IL10 | -0.23 | 0.75198657 | None |
| TNR5 | -0.47 | 0.0410276 | None |
| CYTD | 0.34 | 0.21955776 | None |
| IL12B | 0.28 | 0.38521233 | None |
| CD6 | -0.62 | 0.21103523 | None |
| IL13 | -2.65 | 0.40974586 | None |
| GDNF | 0.53 | 0.06671165 | None |
| LIFR | -0.39 | 0.05293045 | None |
| CXCL5 | -0.75 | 0.13370313 | None |
| FLT3L | -0.35 | 0.04150611 | None |
| ST1A1 | 0.47 | 0.22852894 | None |
| TNF10 | -0.20 | 0.31075658 | None |
| CCL11 | -0.06 | 0.77278616 | None |
| CCL23 | -0.43 | 0.04164615 | None |
| IL2 | -0.17 | 0.18937399 | None |
| X3CL1 | -0.37 | 0.19418626 | None |
| CCL20 | -0.52 | 0.35984873 | None |
| CCL8 | 0.67 | 0.11322393 | None |
| CCL7 | -3.26 | 0.3704584 | None |
| CXCL6 | -0.63 | 0.10991052 | None |
| S10AC | 0.34 | 0.5733337 | None |
| TNR9 | -0.52 | 0.13884329 | None |
| CXCL9 | -0.57 | 0.0823038 | None |
| I10R2 | -0.14 | 0.60458103 | None |
| IL24 | 0.34 | 0.37892388 | None |
| I15RA | -0.34 | 0.15994839 | None |
| SLAF1 | 0.31 | 0.49424712 | None |
| IL18R | -0.18 | 0.38049863 | None |
| 4EBP1 | 0.45 | 0.37408612 | None |
| I10R1 | 0.26 | 0.78687034 | None |
| IL18 | -0.59 | 0.08023487 | None |
| CASP8 | -1.50 | 0.20832384 | None |
| IL17 | 0.38 | 0.24798573 | None |
| ARTN | 0.29 | 0.46371404 | None |
| SIR2 | -0.10 | 0.82345964 | None |
| I22R1 | 0.10 | 0.73568828 | None |
| DNER | 0.22 | 0.18106518 | None |
| TSLP | -0.02 | 0.94676921 | None |
| CCL13 | 0.08 | 0.79744959 | None |
| CCL19 | 0.31 | 0.55121656 | None |
| NRTN | 0.45 | 0.3889046 | None |
| CD244 | -0.77 | 0.19735223 | None |
| FGF23 | -2.42 | 0.10376219 | None |
| CDCP1 | 0.05 | 0.872821 | None |
| CCL28 | 0.01 | 0.98785234 | None |
| FGF21 | -0.89 | 0.15409794 | None |
| IL20 | 0.00 | 0.99827138 | None |
| PD1L1 | -0.31 | 0.08285459 | None |
| IL17C | 0.01 | 0.968213669 | None |
| I20RA | 0.11 | 0.633515885 | None |

Appendix.

Thrombosis Risk Assessment Form (Caprini Model).

| A1 1 point per risk factor | B 2 points per risk factor |
| --- | --- |
| □□ Age 40–59 years old  □□ Minor surgery planned  □□ History of prior major surgery  □□ Obesity (BMI > 30 kg/m^2^)  □□ Medical patient at bed rest  □□ History of inflammatory bowel disease  □□ Swollen legs  □□ Varicose vein  □□ Serious lung disease, including pneumonia (<1 month)  □□ Abnormal pulmonary function (chronic obstructive pulmonary disease)  □□ Acute myocardial infarction (<1 month)  □□ Congestive heart failure (<1 month)  □□ Sepsis (<1 month)  □□ Blood transfusion (<1 month)  □□ Plaster casting or fixation of lower limb  □□ Central venous cannulation  □□ Other risk factors | □□ Age 60–74 years old  □□ Major surgery < 60 min  □□ Laparoscopic surgery > 60 min  □□ Arthroscopic surgery > 60 min  □□ Previous malignancy  □□ Obesity (BMI > 40 kg/m^2^) |
|  | C 3 points per risk factor |
|  | □□ Age > 75 years old  □□ Major surgery 2–3 h  □□ Obesity (BMI > 50 kg/m^2^)  □□ History of superficial/deep vein thrombosis or pulmonary embolism  □□ Family history of thrombosis  □□ Current malignancy or chemotherapy  □□ Heparin-induced thrombocytopenia  □□ Congenital or acquired thrombosis not listed  □□ Anticardiolipin antibodies positive  □□ Prothrombin 20210A positive  □□ Factor Vleiden positive  □□ Lupus anticoagulant positive  □□ Elevated serum homocysteine |
| A2 For women only (1 point each) | D 5 points per risk factor |
| □□ Oral contraceptives or hormone replacement therapy  □□ Pregnancy or postpartum (< 1 month)  □□ History of unexplained stillborn infant, recurrent spontaneous abortion ≥3, premature birth with toxemia or growth-restricted infant | □□ Stroke (<1 month)  □□ Acute spinal cord injury (<1 month)  □□ Elective lower extremity arthroplasty  □□ Hip, pelvis or leg fracture  □□ Multiple traumas (<1 month)  □□ Major surgery > 3 h |
| □□ Total score of risk factors: | |

Notes: ① The weight of each risk factor depends on the possibility of causing thrombotic events (for example, if the score of cancer is 3 points and that of bed rest is 1, the former is more likely to cause thrombus than the latter); ② Only one surgical factor can be selected.

References.

1. Sanikommu SR, Clemente MJ, Chomczynski P, et al. Clinical features and treatment outcomes in large granular lymphocytic leukemia (LGLL). Leuk Lymphoma 2018;59:416-22.
