# Supplementary material for: Thalidomide-based regimen shows promising efficacy in large granular lymphocytic leukemia: a multicenter phase II study
Source: Signal Transduct Target Ther. 2025 Mar 12;10:85. doi: 10.1038/s41392-025-02164-4 (PMC11897152; doi:10.1038/s41392-025-02164-4)
Supplement: Supplementary file 3 — Protocol [file 41392_2025_2164_MOESM3_ESM.pdf]

# Protocol

This trial protocol has been provided by the authors to give readers additional information about the work. Contents are redacted from the Chinese version for Journal use

Contents:

1. Original protocol (version 1.0)
2. Summary of protocol changes and final protocol (version 2.0)

# Phase II Trial of Thalidomide for symptomatic large granular lymphocyte leukemia

## CLINICAL STUDY PROTOCOL

**Protocol Number: BDH/LGLL01**
**Version: 1.0**
**Version Date: 25 April 2020**

**Clinical trial sponsor: State Key Laboratory of Experimental Hematology, National Clinical Research Center for Blood Diseases, Haihe Laboratory of Cell Ecosystem, Institute of Hematology & Blood Diseases Hospital, Chinese Academy of Medical Sciences & Peking Union Medical College, Tianjin 300020, China; Tianjin Institutes of Health Science**

This document contains confidential information, which should not be copied, released or published without Institute of Hematology & Blood Diseases Hospital

Research institutes and participants:

Responsible Person: Shuhua Yi/Lugui Qiu

Sponsor: Institute of Hematology & Blood Diseases Hospital

| **Study Site** | **Lead** |
|---|---|
| Institute of Hematology and Blood Diseases Hospital, Chinese Academy of Medical Sciences | Shuhua Yi/Lugui Qiu |
| The First Affiliated Hospital of Nanchang University | Fei Li |
| The Eighth Affiliated Hospital, Sun Yat-sen University | Yan Chen |
| The People's Hospital of Yongcheng | Xiaoli Hu |
| The Third People's Hospital of Datong | Yaqing Feng |

# Table of Contents


List of Abbreviations ........ 8

1. Synopsis ........ 10

2. Background ........ 16

2.1 Large Granular Lymphocytic Leukemia ........ 16

2.2 Current Treatment Landscape ........ 17

2.3 Investigational Product -Thalidomide ........ 19

2.4 Investigational Product - Methotrexate ........ 23

2.5 Rationale for Thalidomide Combined with Methotrexate and Prednisone in the Treatment of LGLL ........ 26

2.6 Risk/Benefit Assessment ........ 27

2.6.1 Investigational Plan ........ 27

2.6.2 Potential Risks ........ 27

2.6.3 Potential Benefits ........ 28

2.6.4 Assessment of Potential Risks and Benefits ........ 28

3. Objectives ........ 29

4. Study Design ........ 30

4.1 Overall Study Design ........ 30

4.2 Description of Overall Study Design and Plan ........ 30

4.3 Duration of Treatment ........ 31

4.4 Follow-up ........ 31

4.5 Sample Size ........ 31

4.6 Steering Committee ........ 31

4.7 Independent Data Monitoring Committee ........ 32

4.8 Study Termination ........ 32

5 Study Population ........ 33

5.1 Eligibility Criteria for Prescreening ........ 33

5.2 Inclusion and Exclusion Criteria ........ 33

5.2.1 Inclusion Criteria ........ 33

5.2.2 Exclusion Criteria ........ 34

5.3 Withdrawal Criteria ........ 34

5.4 Removal of Patients from Therapy or Assessment ........ 35

5.5 Identification Codes, Enrollment, and Screening Log of Screening Failed Patients 35
6 Treatments ........ 37
6.1 Treatment Regimen ........ 37
6.2 Administration of Study Drugs ........ 37
6.3 Concomitant Medications and Supportive Care ........ 37
6.3.1 Prophylactic Medications ........ 38
6.3.2 Permitted Medications ........ 38
6.3.3 Prohibited Medications ........ 39
6.4 Source and Use of Study Drugs ........ 40
7 Adverse Reactions and Dose Adjustment ........ 41
7.1 Common Adverse Reactions of Thalidomide ........ 41
7.2 Common Adverse Reactions of Methotrexate ........ 42
7.3 Common Adverse Reactions of Prednisone ........ 43
7.4 Guidelines for Dose Adjustment and Discontinuation ........ 44
7.4.1 Dose Adjustment and Discontinuation of Thalidomide ........ 45
7.4.2 Dose Adjustment and Discontinuation of Methotrexate ........ 45
7.4.3 Dose Adjustment and Discontinuation of Prednisone ........ 45
7.5 Other Treatment Considerations ........ 45
8. Study Procedures ........ 46
8.1 Screening Period ........ 46
8.2 Treatment Period ........ 47
8.2.1 Disease Progression ........ 49
8.3 List of Tests and Key Time Points ........ 49
8.4 Early Withdrawal (Discontinuation of Study Treatment) ........ 50
8.5 Follow-up ........ 50
8.5.1 Survival Follow-up ........ 50
8.5.2 Safety Follow-up ........ 51
9. Efficacy Evaluation ........ 52
9.1 Definition ........ 52
9.1.1 General Definition ........ 52
9.2 Efficacy Evaluation ........ 52

9.3 Study Endpoints Definition and Evaluation ....................................... 52
10. Safety Evaluation ................................................................... 54
10.1 Definition and Classification of Adverse Events .............................. 54
10.2 Safety Evaluation ................................................................... 56
10.3 Recording of Adverse Events ....................................................... 57
10.4 Reporting of Adverse Events ....................................................... 58
10.5 Data Safety ........................................................................... 58
11. Statistical Analysis ................................................................. 60
11.1 General Consideration ............................................................. 60
11.2 Safety Analysis ..................................................................... 60
11.3 Efficacy Analysis ................................................................... 60
11.4 Biomarkers Exploratory Analysis ................................................. 61
12. Quality Control and Assurance of Good Clinical Practice ...................... 62
12.1 Monitoring, Audit, and Inspection ................................................ 62
12.2 Investigator's Responsibilities .................................................... 62
13. Ethical and Regulatory Considerations ............................................ 64
13.1 Review and Approval by Institutional Review Board/Independent Ethics Committee ... 64
13.2 Protocol Amendment ............................................................... 64
13.3 Informed Consent ................................................................... 64
14. Data Handling and Documentation .................................................. 66
14.1 Data/Document ...................................................................... 66
14.2 Data Management ................................................................... 66
14.3 Record Retention ................................................................... 66
15. Confidentiality ....................................................................... 67
16. References ........................................................................... 68
17. Appendices ........................................................................... 73

# List of Abbreviations

| Abbreviation | Definition |
|---|---|
| AE | adverse event |
| ALT | alanine aminotransferase |
| AML | acute myeloid leukemia |
| ANC | absolute neutrophil count |
| AST | aspartate aminotransferase |
| CLPD-NK | chronic lymphoproliferative disorders of nature killer cells |
| $C_{max}$ | peak concentration |
| CrCl | creatinine clearance |
| $C_{ssmax}$ | steady-state peak concentration |
| CT | computed tomography |
| CTCAE | Common Terminology Criteria for Adverse Events |
| CTD | cyclophosphamide, thalidomide, and dexamethasone |
| CR | complete remission |
| CRA | clinical research associate |
| CRF | case report form |
| CRR | complete remission rate |
| DoR | duration of response |
| DRO | Data Request Order |
| DSMC | Data Safety Monitoring Committee |
| ECG | electrocardiograph |
| ECOG | Eastern Cooperative Oncology Group |
| EOS | end of study |
| GCP | good clinical practice |
| HBV | hepatitis B virus |
| HCV | hepatitis C virus |
| ICH | International Council for Harmonisation of Technical Requirements for Pharmaceuticals for Human Use |
| IDMC | Independent Data Monitoring Committee |
| ICF | informed consent form |
| IEC | Independent Ethics Committee |
| IL | interleukin |
| IRB | Institutional Review Board |
| LDH | lactate dehydrogenase |
| LGL | large granular lymphocyte |
| LGLL | Large granular lymphocyte leukemia |
| mDoR | median duration of response |
| MDS | myelodysplastic syndrome |
| MM | multiple myeloma |
| MMM | myelofibrosis with myeloid metaplasia |
| NCI | National Cancer Institute |
| NK | nature killer |
| ORR | overall response rate |
| OS | overall survival |
| PD | disease progression |
| PFS | progression-free survival |

| | |
|---|---|
| PR | partial remission |
| QD | once daily |
| QOD | every other day |
| SAE | serious adverse event |
| SD | stable disease |
| STAT3 | signal transducer and activator of transcription 3 |
| TLS | tumor lysis syndrome |
| TNF-α | tumor necrosis factor alpha |
| TPM | thalidomide combined with methotrexate and prednisone |
| T-LGLL | T-cell large granular lymphocytic leukemia |
| TTNT | Time to next treatment |
| ULN | upper limit of normal |
| US | United States |
| WBC | white blood cell |
| WHO | World Health Organization |
| WM | Waldenström macroglobulinemia |

# 1. Synopsis

**Project Version and Date.:** Version 2.0/30 January 2021

**Study Drugs:** Thalidomide, methotrexate, and prednisone

**Study Phase: Phase II**

**Study Title:** Thalidomide for symptomatic large granular lymphocyte leukemia

**Study Population:** Treatment-naive patients, or patients treated with or relapsed from a non-methotrexate /thalidomide-based regimen

**Principal Investigators:** Lugui Qiu and Shuhua Yi

**Primary Objective:**

- To evaluate the complete remission rate (CRR) of symptomatic large granular lymphocyte leukemia (LGLL) patients treated with thalidomide combined with methotrexate and prednisone (TPM) regimen

**Secondary Objectives:**

- To evaluate the safety and tolerance of symptomatic LGLL patients treated with TPM regimen
- To evaluate the overall response rate (ORR) and partial remission (PR) rate of symptomatic LGLL patients treated with TPM regimen
- To evaluate the progression-free survival (PFS) and overall survival (OS) of symptomatic LGLL patients treated with TPM regimen
- To evaluate the time to next treatment (TTNT) and duration of response (DoR) in symptomatic LGLL patients treated with TPM regimen

**Exploratory Objective:**

- To explore biomarkers significantly and potentially associated with clinical efficacy

**Study Design:**

This is a prospective multicenter Phase II clinical study employing an oral regimen administered in 3-month cycles. Patients will receive thalidomide of 50 to 100 mg once daily (QD) before bedtime, prednisone of 0.5 to 1.0 mg/kg every other day (QOD), methotrexate of 10 mg/m$^2$/week for up to 4 cycles. Pred was gradually tapered after 12 months. Patients who achieved a CR could discontinue MTX after one cycle consolidation, with a maximum duration of four cycles. Study Design:

Efficacy evaluation will be conducted at the end of each cycle starting from Cycle 1. The efficacy evaluations will continue until disease progression (PD), discontinuation of treatment (for patients who continue treatment after PD), initiation of a new anti-cancer therapy, or withdrawal from the study (eg, death, patient’s request, loss of follow-up, etc.), whichever occurs first.

The primary objective of this study is to evaluate the efficacy and safety of TPM regimen and further explore the biomarkers associated with clinical efficacy.

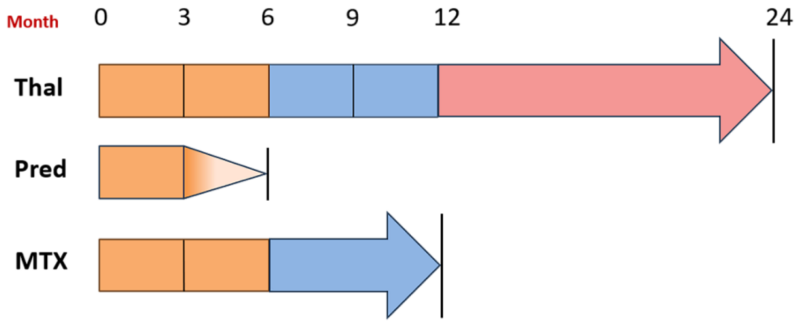


Abbreviations: CR, complete remission; MTX, methotrexate; Pred, Prednisone; Thal, thalidomide.

Notes:

Thal: 50 to 100 mg, oral, before bed, for 2 years

Pred: 0.5to 1 mg/kg, oral, every other day, could be tapered after 12 months

MTX: 10 mg/m$^2$, once weekly, patients who achieve CR can discontinue MTX after 4 months of consolidation therapy, with a maximum duration of 1 year

**Description of Overall Study Design and Plan:**

The study consists of a 28-day screening period, a treatment period (up to half a year for prednisone, up to 1 year for methotrexate, and 2 years for thalidomide), and a follow-up period (safety and efficacy follow-up). Patients who provide the signed informed consent will undergo baseline examinations during the screening period. Prior treatment and efficacy will also be evaluated before enrollment. Patients who met the inclusion/exclusion criteria will enter the treatment period. Relevant examinations specified in the protocol will be completed for all patients during treatment to observe efficacy, safety, and hemogram changes after discontinuation of the study drugs. The follow-up period will begin after the end of the treatment period.

**Duration of Treatment:**

Patients enrolled in this study will be treated with thalidomide combined with methotrexate and prednisone for 3 months and then evaluated for efficacy. Based on the efficacy evaluation, patients will either continue treatment as per the initial regimen or be withdrawn from the study. The Institute of Hematology and Blood Diseases Hospital, Chinese Academy of Medical Sciences, is a specialized hospital for hematology, where treats 30 to 40 LGLL patients every year, of which approximately 5 to 10 are eligible for enrollment. A total of 5 to 10 patients are expected to be enrolled in multiple centers nationwide every year. The enrollment period of this study is expected to span 2 years. This study is of a limited course of treatment. Patients who obtain sustained clinical benefits from the study treatment without consent withdrawal, PD, and intolerable toxicity may continue the study and receive follow-up. The end of study (EOS; study completion) is defined as the time when the last patient completes

the last visit.

**Sample Size:**

A retrospective study of 45 patients is used as the historical control, in which cyclophosphamide monotherapy was applied with an effective rate of 72% and CRR of 47%. From the perspective of clinical expertise, taking the CRR of 70% to 80% as the minimum threshold for clinical significance, the sample size of 35 patients in this group is deemed sufficient to test the overall true CRR that is superior to that of the historical control at a false positive rate $\alpha = 0.05$ (two sides) and false negative rate $\beta = 0.2$ (ie, power = 80%). With a 20% dropout rate, at least 42 patients would be required to enroll in this study.

**Inclusion and Exclusion Criteria:**

**Inclusion Criteria:**

1. Patients who fulfill the diagnostic criteria for T-LGLL or chronic lymphoproliferative disorders of nature killer cells (CLPD-NK) specified in the 2016 World Health Organization (WHO) Classification of Lymphoid Neoplasms [1]
2. Patients are fully informed about the study and willing to participate in the study and provide written informed consent form (ICF)
3. Male or female, aged ≥18 years
4. Treatment-naive patients, or patients treated with or relapsed from a non-methotrexate/ thalidomide-based regimen
5. Patients with at least one of the following indications for LGLL treatment:
    a. absolute neutrophil count (ANC) $<0.5 \times 10^9$/L, or neutropenia with recurrent infections
    b. Hemoglobin <100 g/L, or requiring infusion of red blood cells
    c. Platelets level $<50 \times 10^9$/L
    d. Concomitant autoimmune diseases requiring treatment
    e. Symptomatic splenomegaly
    f. Severe B symptoms (fever of unknown cause with body temperature over 38 °C; night sweats; weight loss of ≥10% within half a year)
    g. Pulmonary hypertension
6. Patients with Eastern Cooperative Oncology Group (ECOG) score of 0 to 2 (Appendix 1)

7. Patients with life expectancy ≥6 months.

**Exclusion Criteria:**

1. Patients not diagnosed with T-LGLL or CLPD-NK

2. Patients with no indication for LGLL treatment

3. Patients who are unable to understand or follow study procedures

4. Patients who have been diagnosed or treated for malignancies other than LGLL within the past five years

5. Patients with non-lymphoma-related hepatic and renal impairment: alanine aminotransferase (ALT) > 3 × upper limit of normal (ULN), aspartate aminotransferase (AST) > 3 × ULN, total bilirubin > 2 × ULN, and serum creatinine clearance (CrCl) <30 mL/min

6. Patients with other severe diseases that have an impact on this study (uncontrolled diabetes, stomach ulcers, other severe cardiopulmonary conditions, etc.), at the discretion of investigators.

7. Patients with a known history of HIV infection, or with active hepatitis B virus (HBV) infection, or any uncontrolled active systemic infection requiring intravenous antibiotics

Notes: HBV infection is considered active if all following criteria are met: a. HBV DNA quantification ≥2000 IU/mL; b. ALT ≥2 times × ULN; c. LGLL, medications, and other causes-induced hepatitis are excluded. Patients with active HBV infection at the time of initial diagnosis who are converted to inactive HBV infection after adequate anti-HBV treatment could be enrolled in this study.

8. Patients who have undergone major surgery (excluding lymph node biopsy) within the past 14 days or who are expected to undergo major surgery during study treatment

9. Pregnant or lactating women, and women of childbearing potential who have not taken contraceptive measures

10. Patients who are allergic to the agents or the ingredients

**Withdrawal Criteria:**

1. Patients withdraw the ICFs

2. Patients experience PD or death

3. Patients experience intolerable toxicity that cannot be relieved after symptomatic treatment

4. Patients are unwilling to continue treatment

5. There was no clinical benefit for more than half a year

6. Delayed dosing for more than 4 weeks and patients are judged by investigators to be unsuitable for further dosing

7. Pregnancy

8. Lost to follow-up

9. Treatment should be discontinued based on the best interests of the patients at the investigator’ discretion

10. Patients does not comply with the protocol, and the investigator deems it necessary to discontinue the treatment

11. The collaborator or regulatory authority notifies to end the clinical study.

**Dosage Form, Dosage, and Route of Administration:**

Treatment will be provided in 3-month cycles. Patients will receive thalidomide of 50 to 100 mg orally QD before bedtime, methotrexate of 10 mg/m$^2$ orally once weekly, and prednisone of 0.5 to 1 mg/kg orally QOD. Prednisone will be tapered after 12 months. Methotrexate will be dosed for up to 1 year. Thalidomide will be dosed for up to 2 years or discontinued based on patients or investigator's choices. Efficacy was evaluated after 3 months of treatment, if PR or above response to treatment, the initial treatment regimen will be continued. If there is no clinical benefit after 6 months of initial treatment, patients will switch to the second-line treatment was and will be withdrawn from the study.

**Study Endpoints:**

**Primary Endpoint:**

- CRR of patients with symptomatic LGLL treated with thalidomide, prednisone, and methotrexate.

**Secondary Endpoints:**

**1. Secondary Safety Endpoint**

- Incidence and severity of hematological and non-hematological adverse events (AEs)

Safety endpoints include serious adverse events (SAEs), treatment-emergent adverse events (TEAEs), physical examination (including ECOG performance status), vital sign measurements, standard clinical laboratory parameters, and electrocardiograph (ECG). TEAEs will be graded according to National Cancer Institute (NCI) Common Terminology Criteria for Adverse Events (CTCAE) Version 5.0.

**2. Secondary Efficacy Endpoints**

- Response rates: ORR and PR rate (time frame: 24 months after the last treatment)

Point estimates of rates will be calculated for each protocol analysis set. An estimate of the 95% CI for the response rate will also be derived. Graphical and descriptive analyses will be used to explore associations between relevant markers and responses.

- PFS and OS (time frame: 24 months after the last treatment)

Time from the first day to PD or death from any reason. The median duration of the overall response will be assessed with a 95% CI.

- DoR (time frame: 24 months after the last treatment)
- TTNT (time frame: 24 months after the last treatment)

The time when CR or PR is met until the first date of recurrent or progressive disease.

The median duration of the overall response will be assessed with a 95% CI.

- Effect of biomarkers on efficacy and adverse reactions

**Exploratory Endpoint:**

- To explore biomarkers significantly and potentially associated with clinical efficacy

**Statistical Analysis:**

The data cut-off date for the primary analysis will be 1 year after the completion of treatment for the last patient. After initial analysis, the primary part of the study will be closed and data will be tracked until completion.

Descriptive statistics will be provided for selected demographic and safety data by group and time as appropriate. For continuous data, descriptive statistics will be presented as mean, median, standard deviation, and range (including geometric mean and geometric coefficient of variation for peak concentration [$C_{max}$]). Categorical data will be summarized as frequency and percentage. Data can also be presented as a graphical abstract.

**Safety Analysis:**

The safety profile will be analyzed based on AEs, physical examination, vital sign measurements, laboratory measurements, and ECG findings. AEs will be graded as per NCI CTCAE (Version 5.0).

In general, the safety analysis will be descriptive and presented in tabular form with appropriate summary statistics. The number of patients and events of hematological and non-hematological toxicity (based on NCI CTCAE Version 5.0) will be listed, and the incidence will be calculated.

**Efficacy Analysis:**

The efficacy analysis will involve CRR and ORR (sum of CR and PR rates). Efficacy variables will be tabulated and summarized. For the numbers and percentages of CRR and ORR, point estimates and 95% exact binomial CIs will be provided.

Time-to-event variables including DoR, TTNT, PFS, and OS will be summarized descriptively using the Kaplan-Meier method. The rules for DoR, TTNT, PFS, and OS, analyses will be specified in the Statistical Analysis Plan (SAP). Growth modulation indices (intra-subject ratios of DOR, PFS, OS, and TTNT post-study treatment versus DOR, PFS, and OS, and TTNT post the most recent prior treatment regimen) will be summarized.

Data will be summarized using descriptive statistics. Continuous variables will be presented as number, mean, standard deviation, median, and range. Discrete variables will be summarized as frequency.

**Biomarker Exploratory Analysis:**

The exploratory analysis of biomarkers will be tabulated and summarized using descriptive statistics.

# 2. Background

## 2.1 Large Granular Lymphocytic Leukemia

Large granular lymphocyte leukemia (LGLL) is a clonal group of lymphoproliferative diseases that originate from CD3+ cytotoxic T cells or CD3- nature killer (NK) cells. Clinically, LGLL is characterized by infiltration of large granular lymphocytes (LGLs) in peripheral blood and bone marrow, as well as splenomegaly and hemocytopenia. In 2008, the World Health Organization (WHO) classified the disease into 3 subtypes by the cell origin and clinical characteristics, including T-cell large granular lymphocytic leukemia (T-LGLL), chronic lymphoproliferative disorders of nature killer cells (CLPD-NK), and aggressive nature killer cell leukemia (ANKL).[1] T-LGLL and CLPD-NK are similar in clinical manifestations, biological characteristics, treatment options and prognosis, both of which have an inert course of disease. They are LGLL in the true sense, and they are also the research objects of this clinical trial. ANKL belongs to aggressive lymphoma, and its treatment and prognosis are different from the previous two, which is not covered by this clinical trial.

As a rare hematological malignancy, T-LGLL accounts for 2% to 5% of chronic lymphoproliferative diseases in North America and up to 6% in Asia. The incidence of T-LGLL is estimated to be 1/10 million in the United States (US),[2] while a registered Dutch study reported an incidence of 0.72/1 million person-years.[3] The median age of onset of T-LGLL is 60 years (range: 4 to 88 years) without a gender difference. Only 10% of T-LGLL patients are younger than 40 years old. T-LGLL is also rare in children.[4]

The exact mechanism by which LGLs clones proliferated remains unclear. It is generally believed that LGLL is caused by chronic antigenic stimulation caused by viral infection. Initially, an unknown antigen leads to the expansion of oligoclonal LGL, and the continuous stimulation of the antigen leads to the activation of signal transducer and activator of transcription 3 (STAT3) and the emergence of dominant cloning. Studies on T cell lineages have confirmed the transition process from oligoclonal to dominant cloning.[5] Both activation of survival pathways and evasion of apoptosis are primary contributors to the clonal proliferation of LGLs in leukemia, with a complex survival network driven by both internal and external stimuli. Multiple dysregulated pathways coordinate pro-survival and anti-apoptotic signals, including resistance to Fas/FasL-mediated apoptosis, activation of interleukin (IL)-15 and platelet-derived growth factor (PDGF), activation of JAK-STAT, P13K-AKT, RAS-RAF MAPK and NF-kB signaling pathways, and dysregulation of the sphingolipid rheostat.[6] The most common mutations in LGLL are the site-directed mutations of the STAT3 gene.[7-8] It is reported to occur in 28% to 75% of patients with T-LGLL. STAT3 forms a dimer upon phosphorylation, which enters the nucleus and functions as a transcription factor to enhance anti-apoptotic pathways, resulting in cytokine-independent proliferation and survival of leukemic cells. Patients with STAT3 mutations have a higher incidence of neutropenia, anemia, and autoimmune diseases, which generally require treatment.[9-10]

LGLL is at the "intersection" of a clonal lymphoproliferative disease, autoimmunity, and chronic inflammation.[11] Chronic antigen stimulation is a trigger for the LGL activation and proliferation, while a strong pro-inflammatory environment plays a crucial role in the pathogenesis of LGLL. Compared with healthy people, multiple cytokines and chemokines (eg, IL-2,[12] IL-6,[13] IL-15[14-15], IL-18,[16] RANTES,[16] and PDGF [17]) significantly increased in the serum of LGLL patients, thereby promoting survival and proliferation of LGL. It has been reported that patients with LGLL could present with a series of changes in immunological indicators (eg, rheumatoid factors, anti-nuclear antibodies, anti-neutrophilic antibodies), highlighting the immune background of this disease.[18] LGL is particularly associated with autoimmune diseases, especially rheumatoid arthritis (observed in 11% to 36% of cases[19]), other hematological diseases and bone marrow failure syndromes (eg, myelodysplastic syndrome (MDS), paroxysmal nocturnal hemoglobinuria, aplastic anemia, pure red cell aplasia) often co-occur with LGLL. This also suggests widespread immune system dysfunction with LGLL.[18] Therefore, the immune mechanism of LGLL serves as a valuable starting point for the development of more effective and better-tolerated drug combinations and clinical regimens.

## 2.2 Current Treatment Landscape

Patients with LGLL do not always need treatment at the time of diagnosis, and patients with newly diagnosed asymptomatic LGLL are observed rather than treated immediately. LGLL cannot be cured by current treatments, and most patients present an indolent disease course with median survival >10 years. Western countries have reported that two-thirds of patients eventually require treatment indications, primarily due to recurrent infections resulting from severe neutropenia.[20] Treatment is required when patients have symptomatic or life-threatening peripheral blood cytopenia, mainly including (meeting at least 1 of the following conditions):

- absolute neutrophil count (ANC) <0.5 × $10^9$/L, or neutropenia with recurrent infections
- hemoglobin <100 g/L, or requiring infusion of red blood cells
- platelets level <50 × $10^9$/L
- Concomitant autoimmune diseases requiring treatment
- Symptomatic splenomegaly
- Severe B symptoms (fever of unknown cause with body temperature over 38 °C; night sweats; weight loss of ≥10% within half a year)
- Pulmonary hypertension

Current treatment options for LGLL are limited, because of the lack of a deep and adequate understanding of its pathogenesis and the absence of effective targeted therapies. At the same time, LGLL is a rare disease with a low incidence and insufficient prospective clinical trials, and there are no standard treatment options for

LGLL. Current treatment options for LGLL include immunosuppressive therapies, purine analogues, chemotherapies with cytotoxic drugs, splenectomy, targeted therapies, and hematopoietic stem cell transplantation.

For T-LGLL and CLPD-NK, immunosuppressants, including methotrexate, cyclophosphamide, and cyclosporin A, are still the most recognized first-line regimen.[21-23] The overall response rate of the 3 immunosuppressants were similar. However, irrespective of the type of immunosuppressant, the low complete response rate indicates that current immunosuppressive treatment has reached the maximum efficacy.

The efficacy of immunosuppressants in LGLL patients is summarized below:

| Product/Regimen | Author (Year) | Number of Cases | ORR, % (n) | CR, % (n) |
|---|---|---|---|---|
| **Methotrexate** | Sanikommu et al (2018)[23] | 34 | 44% (15) | |
| | Bareau et al (2010)[24] | 36 | 44% (16) | 14% (5) |
| | Loughran et al (1994)♯[24] | 10 | 60% (6) | 50% (5) |
| | Loughran et al (2015)♯[25] | 55 | 38% (21) | 5% (3) |
| **Cyclophosphamide** | Sanikommu et al (2018)[23] | 22 | 47% (10) | |
| | Li JY et al (2016)[26] | 36 | 86% (31) | 22% (8) |
| | Moignet et al (2014)[27] | 45 | 72% (32) | 47% (21) |
| | Poullot et al (2014)[28] | 13 | 69% (9) | 46% (6) |
| | Dhodapkar et al (1994)[29] | 16 | 63% (10) | 38% (6) |
| | Sanikommu et al (2018)[23] | 44 | 45% (20) | |
| | Fengkui et al (2020)[30] | 16 | 56% (9) | 31% (6) |
| | Osuji et al (2006)[31] | 14 | 92% (13) | |
| **Cyclosporin A + Prednisone** | Fengkui et al (2020)[30] | 83 | 48% (40) | 18% (15) |

♯ Prospective studies

Based on the pathogenesis of T-LGLL, it is speculated that potentially effective novel drugs may include monoclonal antibodies (eg, anti-CD2, CD22, and CD52 antibodies), small molecule-targeted drugs (eg, RAS inhibitors [tipifarnib]), JAK3-specific inhibitors (tofacitinib), PI3K inhibitors and proteasome inhibitors (eg, bortezomib).[32-33] At present, alemtuzumab and tofacitinib are the main applications and shown to be effective. The efficacy and side effects of novel drugs are not yet known, so a larger patient population needs to be involved in clinical trials for further validation. Meanwhile, most new drugs are expensive and inaccessible, rendering them unsuitable as first-line treatments for LGLL patients. Hematopoietic stem cell transplantation and splenectomy are not routine treatments for LGLL and are only considered if a patient is resistant to most drugs.

To sum up, there is currently no standard first-line treatment regimen for LGLL, and addressing the challenge of providing patients with more profound and durable

remission remains a pressing clinical issue. The TPM regimen (thalidomide combined with methotrexate and prednisone) has been employed in our site for the treatment of LGLL since 2013. Preliminary results showed that a total of 20 patients were enrolled, of whom 20 (90.0%) achieved hematological remission and 16 (80.0%) achieved complete remission (CR), demonstrating favorable preliminary efficacy. Grade ≥3 adverse events (AEs) were rare, demonstrating favorable safety. Therefore, we designed this study to observe the efficacy and safety of TPM regimen in patients with symptomatic LGLL.

## 2.3 Investigational Product -Thalidomide

Thalidomide, also known as 2-phthalimidoglutarimide, has been widely used in early pregnancy due to its sedative and antiemetic effects, and later due to its severe teratogenicity, it caused the thalidomide tragedy and was banned from use in many countries. But the study of thalidomide did not stop there. With the continuous exploration of the pharmacological mechanism of thalidomide, it has been found that thalidomide has the effect of anti-angiogenesis and inhibition of tumor necrosis factor alpha (TNF-α), so that is widely used in immune diseases and various malignant tumors.[34-36] At present, thalidomide has been approved for multiple myeloma (MM), erythema nodosum leprosum, graft versus host disease, recurrent aphthous ulcer in HIV infection/Behcet's syndrome, paraneoplastic sweating, paraneoplastic and uremic pruritus, cachexia in HIV and cancer, intractable gastrointestinal hemorrhage, refractory irinotecan-induced diarrhea, discoid lupus erythematosus, rheumatoid arthritis, etc.

**Pharmacokinetics of Thalidomide**

Thalidomide is interconvertible between (R)- and (S)- enantiomers in plasma, with protein binding rates of 55% and 65%, respectively. More than 90% of the absorbed drugs are excreted through urine and feces within 48 hours. Thalidomide is little metabolized in the liver, but it spontaneously hydrolyzes into a variety of renal excreted metabolites.

After a single oral dose of thalidomide of 200 mg (the United States approved capsule form) in healthy volunteers, thalidomide was slowly absorbed and reached a peak concentration ($C_{max}$) of 1 to 2 mg/L 3 to 4 hours after administration, the absorption lag time was 30 minutes, the total exposure (AUCβ) was 18 mg ± h/L, the elimination half-life was 6 hours, and the total clearance rate was 10 L/h. Due to the low solubility of thalidomide in the gastrointestinal tract, the absorption rate of thalidomide was limited, and its elimination rate was faster than the absorption rate.

Multiple doses of thalidomide 200 mg/day for 21 days will not change pharmacokinetics, with a steady-state peak concentration ($C_{ssmax}$) of 1.2 mg/L. Simulated multiple doses of 400 mg/day and 800 mg/day also showed no accumulation, with $C_{ssmax}$ of 3.5 and 6.0 mg/L, respectively. Multiple dose studies in cancer patients showed that pharmacokinetic profile was similar to that in healthy population at similar

doses. At dose range of 50 to 400 mg, thalidomide showed a proportional increase in area under the concentration-time curve with increasing doses. Due to the low solubility of thalidomide, $C_{max}$ is not proportional to the dose, while the time to reach the maximum plasma concentration is prolonged with increasing doses. Age, sex, and smoking have no effect on the pharmacokinetics of thalidomide, and food has little effect. Thalidomide does not change the pharmacokinetics of oral contraceptives and is unlikely to interact with warfarin and grapefruit juice. Since thalidomide is primarily excreted by hydrolysis and passive excretion, pharmacokinetics is unlikely to be changed in patients with impaired liver or kidney function.

**Anti-tumor Mechanisms of Thalidomide**

The exact mechanism of thalidomide as an antineoplastic agent is not fully understood, but some studies have suggested that it may have anti-tumor effects on tumor cells through a variety of pathways. The following are some possible anti-tumor mechanisms: [34, 36]

- Antiangiogenic effect: Thalidomide may reduce the tumor blood supply by inhibiting angiogenesis. It is thought to affect some growth factors associated with angiogenesis, such as vascular endothelial growth factor (VEGF), basic fibroblast growth factor (bFGF), and TNF-α, thereby inhibiting the angiogenesis of tumor cells.
- Immunomodulatory effects: Thalidomide may have an effect on the immune system, enhancing the body’s immune response to tumor cells. It may promote the activity of NK cells, improve the immune response of T cells, and reduce the utilization of immune escape mechanisms by tumor cells.
- Anti-inflammatory effect: Thalidomide has anti-inflammatory effects and may affect tumor growth by reducing inflammation in the tumor microenvironment. It can reduce the activity of inflammatory cells and reduce the production of inflammation-related factors.
- Immunoregulatory apoptosis: Thalidomide is thought to induce apoptosis of tumor cells, thereby reducing tumor load. It may achieve this effect by altering cytokine production and cell signaling pathways.

**Thalidomide in the treatment of Hematologic Tumors**

The first in human Phase I clinical study of thalidomide was conducted in the early 1950s. The study was initially conducted on the drug as a sedative and hypnagogue. However, it was later discovered that thalidomide could cause severe congenital malformations, and its use was discontinued. Thalidomide has not been reported in the treatment of LGLL, but it has been used in hematologic tumors, including MM, Waldenström macroglobulinemia (WM), and MDS.

**Study on Thalidomide in the Treatment of Multiple Myeloma**

MM is a disease that cannot be cured despite active treatment such as high-dose chemotherapy and stem cell transplantation. The theoretical basis for treatment with thalidomide is primarily based on the observation of enhanced neoangiogenesis in bone marrow of patients with progressive disease, and the potential anti-angiogenic effects through the TNF-α, basic fibroblast growth factor (bFGF), and vascular endothelial growth factor (VEGF) in preclinical studies. A study team of the University of Arkansas reported for the first time the efficacy and safety of thalidomide monotherapy in refractory MM.[37] Of the 84 evaluable patients who received 200 mg of thalidomide (gradually increased to 800 mg), serum M-protein concentration was reduced by more than half in 25% of the patients and by more than 90% in 8 patients. Two CRs were observed, and these remissions were durable because the median time to progression had not been reached in the 14-month follow-up. Although more than 3 quarters of patients experienced a response consistent with reduced plasma cell infiltration in the bone marrow, the therapy had no effect microvascular density. Further follow-up of 169 patients showed that 2-year event-free survival and overall survival (OS) were 20% and 48%, respectively. In addition, high-dose thalidomide was associated with improved survival in high-risk patients, supporting the opinion of the dose-related effect. The response to thalidomide in patients with MM usually occurs after 1 to 2 months of treatment of 200 to 400 mg daily, a dose of 50 mg daily may be sufficient as a maintenance therapeutic dose for some patients if considering the clinical response.

The main toxicity of thalidomide observed in these patients included neurological events (somnolence, dizziness, delirium, tremor, uncoordinated movement, stinging and numbness), gastrointestinal disorders (constipation, nausea, vomiting and stomatitis) and systemic symptoms (weakness, weight loss, and fever). It is concluded that thalidomide had significant anti-tumor activity in advanced high-risk MM, the clinical data on early single agent were more strongly supported, and based on early favorable results for refractory myeloma, thalidomide was granted as an orphan drug, providing it with a 7-year protective study and development period. CC-5013, an immunomodulatory derivative of thalidomide, has good anti-tumor effects and shows promising results in relapsed and refractory myeloma, with a 25% reduction in M protein concentration in 71% of 24 treated patients.[38] Thalidomide is currently used for the treatment of patients with advanced refractory or newly diagnosed MM, and is also of important value in maintenance therapy.

**Study on Thalidomide in the treatment of Waldenström Macroglobulinemia**

WM is a malignant lymphoplasmacytic lymphoma that may cause symptoms and complications due to infiltration of bone marrow, spleen, or lymph nodes. A Phase II study of thalidomide in WM enrolled 20 patients with a median age of 74 years (range: 48 to 85).[39] Ten of the patients were previously untreated, while the other 10 were previously treated, including 4 who did not respond to any previous course (initially refractory), 5 who relapsed despite chemotherapy (refractory relapse), and 1 who relapsed without treatment. Approximately one-third of patients had at least 1 of severe anemia, splenomegaly, or elevated serum β2-microglobulin. The initial dose of thalidomide was 200 mg orally per day, taken before bedtime. The dose was gradually

increased by 200 mg every 14 days up to a maximum dose of 600 mg depending on patient tolerance. Five of the 20 patients (25%) achieved partial remission (PR), defined as a reduction of >50% in tumor infiltration at all involved sites. Five patients were rated as stable disease (SD), while 10 showed early disease progression (PD) with shorter remission duration, ranging from 0.8 to 2.8 months. PR was observed in 3 of 10 previously untreated patients and 2 of 10 previously treated patients. Of the 5 patients treated during refractory relapse and 7 patients treated for more than 24 months, none responded to thalidomide. For all patients, the median time to progression was 5 months and the median duration of response (mDoR) was 11 months.

Thalidomide has a variety of side effects, such as peripheral neuropathy, constipation, and nausea. These side effects are more commonly reported in patients over 70 years of age. Therefore, the dose of thalidomide was gradually increased to the target dose of 600 mg in only 5 patients. Four patients only received a maximum dose of 200 mg, and 11 patients received a maximum dose of 400 mg. Ten patients were treated with thalidomide for 2 months, 3 of whom discontinued thalidomide within 30 days of the first administration. The main cause for lowering the maximum dose of thalidomide or early discontinuation of treatment was intolerance.

**Study on Thalidomide in the Treatment of Light Chain Amyloidosis**

Thalidomide has also been used in some patients with AL amyloidosis. In one study, a significant reduction in urine M-protein was observed in 25% of patients. Grade 3 or 4 toxicity occurred in 50% of patients, and thalidomide was discontinued due to side effects in 25% of patients. Fatigue and other central nervous system toxicities were major dose-limiting toxicities.[40]

In another study, dexamethasone was used in combination with thalidomide at a starting dose of 100 mg daily in increments of 100 mg every 2 weeks up to 400 mg. Decreased M-protein and more than 50% reduction in proteinuria were observed in 59% of all affected patients. Serious side effects included bradycardia, which led to treatment interruption in 65% of patients. Six patients receiving a dose of 400 mg responded to treatment, while none of the 4 patients receiving 100 mg daily responded to treatment.[41]

**Study on Thalidomide in the Treatment of Myelofibrosis**

Myelofibrosis with myeloid metaplasia (MMM) is a proliferative disorder accompanied by myelofibrosis, ectopic hematopoiesis, and increased microvessel density. Several Phase II studies have evaluated the efficacy and tolerability of thalidomide in MMM.[42-45] Improvements in thrombocytopenia and anemia appeared to be the main benefits from the treatment. Significant spleen shrinkage was observed in less than 20% of patients. Adverse hematological effects, such as marked leukocytosis and thrombosis, were observed in some patients. In addition, up to 50% of patients discontinued thalidomide due to side effects. Therefore, multiple studies have confirmed that thalidomide was associated with clinical benefit in patients with MMM, but its tolerability was a major concern in this disease. A Phase II trial conducted by Mesa et

al investigated the effect and tolerability of low-dose thalidomide and prednisone in patients with MMM.[46] Twenty-one patients received 50 mg of thalidomide daily along with oral prednisone for 3 months. Thalidomide and prednisone were well tolerated, with a treatment response in 13 patients (62%). Of the 10 patients requiring blood transfusion, 7 improved and 4 no longer required blood transfusion. Six of the 8 patients with thrombocytopenia had platelet counts increased by more than 50%; and of the 21 patients, 4 had spleen shrinkage by more than 50%. Low doses of thalidomide and prednisone appeared to be more tolerated, meanwhile improving therapeutic effects. Thalidomide and prednisone need to be further evaluated in the early stages of MMM. This combination is a reasonable treatment option for patients with MMM who mainly present with cytopenia. Two long-term outcome analysis trials conducted by the Mayo Clinic, which included 36 patients, showed that thalidomide monotherapy or in combination with prednisone achieved an overall response in 19 patients (53%). In addition, 19% of patients remained in remission without additional medication for a median of 17 months after discontinuation of thalidomide.[47]

**Study on Thalidomide in the Treatment of Myelodysplastic Syndrome**

The immunomodulatory and antiangiogenic effects of thalidomide have prompted the evaluation of its use in the treatment of MDS. In a large-scale study, Raza et al treated 83 MDS patients with thalidomide at a starting dose of 100 mg/day, which was gradually increased to a maximum of 400 mg/day, if tolerated. Fifty-one patients completed 3 months of treatment with an overall response rate (ORR) of 19%; hemoglobin rebounded in 15 patients, 10 no longer required blood transfusion, and 1 patient had increased platelets. The mDOR was 306 days. Patients who were more likely to benefit were those with lower primitive naïve cells and higher baseline platelet counts.[48] Thalidomide was administered to 30 patients with MDS in a study conducted by Zorat et al. Hemoglobin rebounded in 10 patients, and 6 no longer required blood transfusion. Clinical responses were more common in patients with higher baseline platelet counts and lower percentage of outbreaks.[49] Stupp et al treated 34 patients and observed hematological improvements in 56% of the patients. The quality of life was improved in responders.[50]

**Study on Thalidomide in the Treatment of Acute Myeloid Leukemia**

Angiogenesis is involved in the pathophysiology of acute myeloid leukemia (AML). Twenty AML patients with poor prognosis received 200 to 400 mg of thalidomide daily. Four of these patients achieved a PR, defined as decreased bone marrow infiltration in leukemia with improvement in red blood cell and platelet counts.[51] A thalidomide trial of 16 patients with refractory or relapsed AML showed that 1 patient achieved CR for 36 months and two patients had transient reductions in bone marrow outbreaks. There was no correlation between the reduced levels of angiogenesis and response.[52] Therefore, thalidomide is not recommended in patients with AML outside of clinical trials.

## 2.4 Investigational Product - Methotrexate

Methotrexate, commonly referred to as MTX for short, is widely used agent in the medical field with a variety of uses, including anti-cancer therapy, treatment of autoimmune diseases, and the control of inflammatory diseases. Details of methotrexate are provided as follows:

**Pharmacokinetics of Methotrexate**

In the treatment of inflammatory autoimmune diseases, methotrexate is usually taken orally once a week. In clinical practice, treatment typically starts at a dose of 10 mg per week and is increased by 5 mg every 2 to 4 weeks, up to a maximum dose of 20 to 30 mg per week, which will vary depending on patient's clinical response and tolerance. In recent years, there is increasing interest in the injectable use of methotrexate, especially in the subcutaneous form, which has greater benefits than oral administration. Compared with the oral form, subcutaneous injection of methotrexate showed better clinical efficacy and tolerability. Subcutaneous injection of methotrexate is currently recommended in cases of where oral methotrexate is ineffective or poorly tolerated. In juvenile idiopathic arthritis, methotrexate has shown to be effective at doses of 10 to 20 $mg/m^2$ body surface area.

After oral administration of methotrexate, its absorption occurs in the proximal jejunum, mainly through the proton-coupled folate transporter (PCFT/SLC46A1), which transports reduced folic acid and methotrexate. In addition, part of methotrexate may be metabolized to 4-amino-4-deoxy-N10-methylpara aminobenzoic acid with the involvement of intestinal bacteria. The bioavailability of methotrexate is relatively high, generally in the range of 64% to 90%, but varies widely between patients and decreases with increasing doses, with stable bioavailability at doses greater than 15 mg per week, indicating intestinal transporter saturation. Methotrexate, when taken orally, has a $C_{max}$ of between 0.3 to 1.6 μmol/L, generally occurring 0.75 to 2 hours after administration. Multiple studies have demonstrated that subcutaneous injection of methotrexate has higher bioavailability compared with oral methotrexate. Subcutaneous injection of methotrexate resulted in linear, dose-proportional increases in blood drug concentrations, with no dose saturation effect. Approximately 50% of circulating methotrexate binds to plasma protein. Methotrexate can be distributed into synovial fluid at concentrations comparable to those in plasma. Methotrexate undergoes the first-pass effect metabolism in the liver and is converted to 7-hydroxymethotrexate (7-OH-MTX), a major metabolite of methotrexate. Renal excretion is the main route of excretion for methotrexate. Methotrexate is filtered through the glomeruli, as well as active tubular secretion and reabsorption. A small portion of methotrexate is excreted through bile and a portion enters hepatic-enteric circulation. The plasma half-life of low dose of methotrexate is between 4.5 to 10 hours. The excretion of methotrexate is reduced in patients with impaired renal function, ascites, or pleural effusion, who need to be particularly careful to monitor toxic effects and may need to reduce the dose or in some cases discontinue methotrexate therapy.

Cellular uptake of methotrexate is mediated by reduced folate carrier 1 (RFC1/SLC19A1), with limited contributions from α and β folate receptors. The

outflow of intracellular methotrexate is regulated by ATP-binding cassette transporters (ABCC), which transport many drugs and chemotherapeutic agents. Intracellularly, part of intracellular methotrexate is converted to methotrexate polyglutamate under the catalysis of folate polyglutamate synthase, which adds up to 7 glutamic acid residues to methotrexate. Methotrexate derivatives with more than 3 glutamic acid residues are not substrates of ABCC and therefore have greater retention ability in cells. Polyglutamylation can be reversed by a deglutamylation process catalyzed by gamma-glutamyl hydrolase, resulting in steady-state intracellular methotrexate levels. After oral administration, methotrexate polyglutamate is found in red blood cells, neutrophils, monocytes, hepatocytes, and synovial cells. The accumulation of intracellular methotrexate polyglutamate leads to sustained efficacy and allows weekly administration of methotrexate, despite its relatively short plasma clearance half-life.

**Clinical Experience with Methotrexate**

Methotrexate is widely used in the treatment of patients with rheumatoid arthritis (RA), and in a subset of RA patients, it is closely related to LGLL. LGLL exhibits activation of multiple survival signaling pathways, among which the JAK/STAT pathway has shown to be involved with LGL transformation.[53] Low-dose methotrexate has anti-inflammatory and immunosuppressive mechanisms that can effectively inhibit the JAK/STAT pathway.[54] As a standard first-line treatment, methotrexate has good efficacy in patients with neutropenia and autoimmune diseases. At the same time, methotrexate has been successful in treating patients with neutropenia caused by Felaty syndrome, while patients with LGLL often present with neutropenia. Therefore, in 1994, Loughran et al reported for the first time the results of prospective clinical trials on methotrexate of T-LGLL.[55] Ten patients were treated with methotrexate at 10 $mg/m^2$ per week in this study, some of whom also received prednisone. Five of these patients achieved a complete hematologic remission, 3 of them achieved a molecular response with the disappearance of T-cell clones. The mDOR was 3.8 years.

In a large series of studies in 2010 which involved 62 patients, the ORR was 55% in 36 patients treated with methotrexate, and a large percentage of patients relapsed after treatment. Twelve of the 18 patients (67%) relapsed after more than one year follow-up. The onset time was 2 to 12 weeks.[56] In general, methotrexate could be continued indefinitely due to its good tolerability. Neutropenia gradually reappeared even in patients who achieved a complete molecular response.

In 2015, Loughran et al reported the results of the second T-LGLL prospective Phase II clinical study.[25] The study enrolled 55 patients who received oral methotrexate at 10 $mg/m^2$ per week as initial treatment, and those who failed the methotrexate treatment were eligible for cyclophosphamide with 100 mg orally daily. The ORR of methotrexate was 38%, and the complete remission rate (CRR) was 5%. For patients with neutropenia, the ORR was 42%. The ORR in patients with anemia was 34%. Serum biomarker studies confirmed the inflammatory environment of LGL, but LGL is not a predictor of response. Also, response-related gene expression characteristics were confirmed, and it was speculated that gene expression was driven by STAT3

mutations, while methotrexate as an immunosuppressant was a suitable and effective treatment option.

## 2.5 Rationale for Thalidomide Combined with Methotrexate and Prednisone in the Treatment of LGLL

Methotrexate is one of the main therapeutic agents for LGLL at present. The ORR of single drug treatment for LGLL is 40% to 60%, but the CRR is low which is usually less than 10%. As an indolent lymphoma, disease recurrence is inevitable. How to improve the CRR, progression-free survival (PFS), OS, and improve the quality of life are still difficult issues at present.

Although there is currently no direct evidence of thalidomide combined with methotrexate and dexamethasone, a retrospective study in 2007 reported oral regimens using cyclophosphamide, thalidomide, and dexamethasone (CTD) or attenuated CTD (CTDa) in patients with AL amyloidosis.[57] A total of 75 advanced patients (including 44 patients with clonal relapse after prior treatment) were enrolled, of whom 51 (68%) received CTD and 24 (32%) received CTDa. Of the 65 evaluable patients, 48 (74%) had a hematologic response, of whom 14 (21%) had a CR and 34 (53%) had a PR. The median estimated OS from the start of the treatment was 41 months, and the OS from diagnosis was not reached with a median follow-up time of 22 months. The 3-year estimated OS for complete and partial hematological responders was 100% and 82%, respectively. The incidence of toxicity requiring treatment to be discontinued was 8%, and 52% of patients had ≥Grade 2 events. The TRM was 4%. The clonal response rate to CTD reported was higher than that previously reported for AL amyloidosis non-transplant regimen, and it allows the use of CTD in patients at low risks. Prospective randomized study of CTD is needed.

A Phase II clinical study showed that thalidomide combined with cyclophosphamide and dexamethasone had good efficacy and safety in patients with idiopathic Castleman's disease.[58] A total of 25 patients were enrolled, and patients received thalidomide with 100 mg before bed for 2 years. Cyclophosphamide was taken orally at 300 mg/m$^2$ on Days 1, 8, 15, and 22, 4 weeks as a cycle for a total of 1 year. Prednisone was given at 1 mg/kg on Days 1 and 2, 8 and 9, 15 and 16, 22 and 23, 4 months as a cycle for a total of 1 year. This regimen lasted for 2 years or until treatment failure. One patient in this study died of pulmonary infection and 1 developed a Grade 3 rash. There were no other AEs with Grade ≥ 3. Grade 1 or 2 constipation (40%), pruritus (20%), rash (16%), peripheral sensory neuropathy (16%), and nausea (16%) were the most common AEs. Other AEs included irregular menstruation (12.5% in female patients), glucose intolerance (12%), alanine aminotransferase increased (12%), pneumonia (12%), sexual hypoactivity (12%), leukopenia (8%), peripheral neuropathy (motor) (8%), erectile dysfunction (5.8% in male patients), neutropenia (4%), and hepatitis B virus reactivation (4%). No thrombotic events were observed and no hemorrhagic events were reported during follow-up. The results suggested that

thalidomide combined with immunosuppressants and glucocorticoids was a safe combination.

Since 2013, the Institute of Hematology and Blood Diseases Hospital, Chinese Academy of Medical Sciences has been using TPM regimen to treat LGLL, ie, thalidomide of 50 to 100 mg/night, prednisone of 0.5 to 1.0 mg/kg every other day (QOD), and methotrexate of 10 mg/m$^2$/week. The regimen will continue for up to 12 months until PD or intolerance. Preliminary results showed that a total of 20 patients were enrolled, of which 20 (90.0%) achieved hematological remission, 16 (80.0%) achieved CR, demonstrating favorable preliminary efficacy. Grade ≥3 AEs were rare, and the regimen had good safety profile. The preliminary data showed that TPM had a high effective rate and good safety and tolerability in the treatment of LGLL.

Thalidomide combined with immunosuppressants and hormones showed combined efficacy in other hematological tumors, and adverse reactions did not increase significantly demonstrating good safety profile. The preliminary data of this regimen in LGLL also proved its efficacy and safety, thus, this study further explores the TPM combination therapy for LGLL.

## 2.6 Risk/Benefit Assessment

### 2.6.1 Investigational Plan

This is a prospective multicenter Phase II clinical study employing an oral regimen administered in 3-month cycles. Patients will receive thalidomide of 50 to 100 mg once daily (QD) before bedtime, prednisone of 0.5 to 1.0 mg/kg QOD, methotrexate of 10 mg/m$^2$/week for up to 4 cycles. Prednisone will be tapered after 12 months. Patients who achieve CR can discontinue methotrexate after 4 months of consolidation therapy, with a maximum duration of 1 year. For patients who achieve PR or CR, thalidomide will be dosed for up to 2 years or discontinued at the investigator's discretion based on clinical symptoms.

Efficacy evaluation will be conducted at the end of each cycle starting from Cycle 1. The efficacy evaluations will continue until PD, discontinuation of treatment (for patients who continue treatment after PD), initiation of a new anti-cancer therapy, or withdrawal from the study (eg, death, patient's request, loss of follow-up, etc.), whichever occurs first.

The primary objective of this study is to evaluate the efficacy and safety of TPM regimen and further explore the biomarkers associated with clinical efficacy.

### 2.6.2 Potential Risks

According to the preliminary summarized clinical safety data, the adverse reactions of thalidomide combined with immunosuppressants and hormones regimen were mainly gastrointestinal reactions and peripheral neuropathy, manifested as numbness of hands and feet, loss of appetite, constipation, dizziness, etc. However, the adverse reactions were mainly Grade 1 or 2 and could be managed. The most severe toxicities of thalidomide were embryo-fetal toxicity and venous thromboembolism. Due to the risk

of embryo-fetal toxicity and venous thrombosis. Pregnant and lactating women will be excluded from the study and those who become pregnant during the study will be promptly withdrawn from the study. Patients will take aspirin concurrently with oral thalidomide to prevent venous thromboembolism and coagulation function will be monitored at the end of each cycle. The preliminary data of this study showed that except for 1 Grade 3 nausea, no other Grade ≥3 adverse reactions occurred and all adverse reactions were clinically manageable. The safety of thalidomide combined with methotrexate and prednisone will be monitored in this study.

### 2.6.3 Potential Benefits

Both prospective and retrospective data indicated that the current immunosuppressive treatment had a low CRR in patients with LGLL, and the current immunotherapy has reached the “ceiling”. Addressing the challenge of providing patients with more profound and durable remission remains a pressing clinical issue to be solved. LGLL is an immune disorder, immunosuppression alone is not the best choice. As an immune modulator, thalidomide combined with immunosuppressants can enhance the body's immune response to tumor cells, promote the activity of NK cells, improve the immune response of T cells, reduce the utilization of immune escape mechanisms by tumor cells, and be more targeted for the treatment of LGLL. At the same time, previous data showed that the TPM regimen had a high effective rate and CRR, far exceeding the current immunosuppressants. In addition, thalidomide combined with immunosuppressants and hormones showed good safety in hematologic tumors. Therefore, it is predicted that the TPM regimen in the treatment of LGLL can further improve the effective rate and deep response rate in patients with LGLL while ensuring the safety of patients.

### 2.6.4 Assessment of Potential Risks and Benefits

Thalidomide combined with immunosuppressants and hormones has been used in a variety of hematologic treatments. The adverse reactions are mainly gastrointestinal reactions and peripheral neuropathy, with sufficient safety information and manageable risks. Previous data showed that TPM regimen had a good effective rate and CRR in patients with LGLL. In this study, the adverse reactions of patients during treatment will be closely monitored and treated in a timely manner until they return to normal or reach clinical stability.

Therefore, the risk of this study is manageable, and the expected benefits outweigh the risks.

# 3. Objectives

**Primary Objective:**

- To evaluate the CRR of symptomatic LGLL patients treated with TPM regimen

**Secondary Objectives:**

- To evaluate the safety and tolerance of symptomatic LGLL patients treated with TPM regimen
- To evaluate the ORR and PR rate of symptomatic LGLL patients treated with TPM regimen
- To evaluate the PFS and OS of symptomatic LGLL patients treated with TPM regimen
- To evaluate the time to next treatment (TTNT) and duration of response (DoR) in symptomatic LGLL patients treated with TPM regimen
-

**Exploratory Objective:**

- To explore biomarkers significantly and potentially associated with clinical efficacy

# 4. Study Design

## 4.1 Overall Study Design

This is a prospective multicenter Phase II clinical study employing an oral regimen administered in 3-month cycles. Patients will receive thalidomide of 50 to 100 mg QD before bedtime, prednisone of 0.5 to 1.0 mg/kg QOD, methotrexate of 10 mg/m$^2$/week for up to 4 cycles. Prednisone will be tapered after 12 months. Patients who achieve CR can discontinue methotrexate after 4 months of consolidation therapy, with a maximum duration of 1 year. For patients who achieved a partial remission (PR) or CR, Thal maintenance therapy was recommended to be continued for a maximum duration of two years, but also depended on patients or investigator's choices.

Efficacy evaluation will be conducted at the end of each cycle starting from Cycle 1. The efficacy evaluations will continue until PD, discontinuation of treatment (for patients who continue treatment after PD), initiation of a new anti-cancer therapy, or withdrawal from the study (eg, death, patient's request, loss of follow-up, etc.), whichever occurs first.

The primary objective of this study is to evaluate the efficacy and safety of TPM regimen and further explore the biomarkers associated with clinical efficacy.

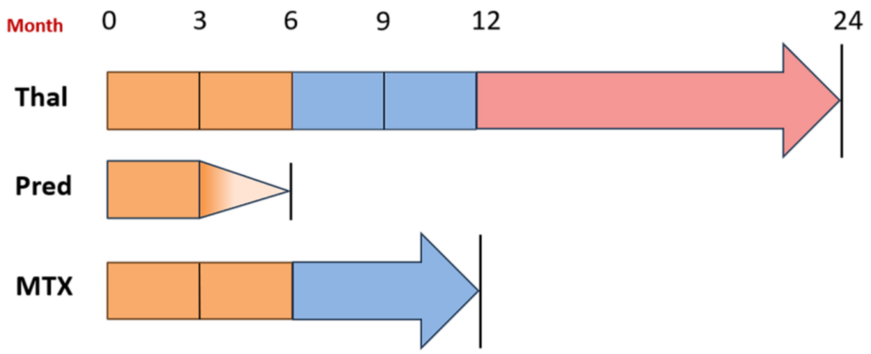


Abbreviations: CR, complete remission; MTX, methotrexate; Pred, Prednisone; Thal, thalidomide.
Notes:
Thal: 50 to 100 mg, oral, before bed, for 2 years;
Pred: 0.5-1 mg/kg, oral, every other day, could be tapered after 12 months
MTX: 10 mg/m$^2$, once weekly, patients who achieve CR can discontinue MTX after 4 months of consolidation therapy, with a maximum duration of 1 year.

## 4.2 Description of Overall Study Design and Plan

The study consists of a 28-day screening period, a treatment period (up to half a year for prednisone, up to 1 year for methotrexate, and 2 years for thalidomide), and a follow-up period (safety and efficacy follow-up). Patients who provide the signed informed consent will undergo baseline examinations during the screening period. Prior

treatment and efficacy will also be evaluated before enrollment. Patients who met the inclusion/exclusion criteria will enter the treatment period. Relevant examinations specified in the protocol will be completed for all patients during treatment to observe efficacy, safety, and hemogram changes after discontinuation of the study drugs. The follow-up period will begin after the end of the treatment period.

## 4.3 Duration of Treatment

Patients enrolled in this study will be treated with thalidomide combined with methotrexate and prednisone for 3 months and then evaluated for efficacy. Based on the efficacy evaluation, patients will either continue treatment as per the initial regimen or be withdrawn from the study. The Institute of Hematology and Blood Diseases Hospital, Chinese Academy of Medical Sciences, is a specialized hospital for hematology, where treats 30 to 40 LGLL patients every year, of which approximately 5 to 10 are eligible for enrollment. A total of 5 to 10 patients are expected to be enrolled in multiple centers nationwide every year. The enrollment period of this study is expected to span 2 years. This study is of a limited course of treatment. Patients who obtain sustained clinical benefits from the study treatment without consent withdrawal, PD, and intolerable toxicity may continue the study and receive follow-up. The end of study (EOS; study completion) is defined as the time when the last patient completes the last visit.

## 4.4 Follow-up

Patients who withdraw from the study due to intolerable AEs will be followed up in this study until the events resolve to Grade 1 or lower, or the AE is stable. Patients will be followed up for up to 24 months after completion of the treatment, or until study termination, PD, initiation of new anti-tumor therapy, or death.

## 4.5 Sample Size

A retrospective study of 45 patients is used as the historical control, in which cyclophosphamide monotherapy was applied with an effective rate of 72% and CRR of 47%. From the perspective of clinical expertise, taking the CRR of 70% as the minimum threshold for clinical significance, the sample size of 35 patients in this group is deemed sufficient to test the overall true CRR that is superior to that of the historical control at a false positive rate $\alpha = 0.05$ (two sides) and false negative rate $\beta = 0.2$ (ie, power = 80%). With a 20% dropout rate, at least 42 patients would be required to enroll in this study.

## 4.6 Steering Committee

A Steering Committee will be established for this study. The Steering Committee will consist of investigator participating in the study, an additional study physician, and a statistician. Study design and inclusion/exclusion/withdrawal criteria will be

determined by the Steering Committee.

Members of the Steering Committee will periodically review the status of enrolled patients and adjudicate on situations not covered by existing inclusion, exclusion, or withdrawal criteria.

## 4.7 Independent Data Monitoring Committee

The Independent Data Monitoring Committee (IDMC) will review the trial data to monitor the quality of trial operations, including the protocol compliance, recruitment status, dropout rate, and data integrity. The IDMC will monitor the accumulated patient safety data every 1 month upon request. The IDMC may propose recommendations on adjustment of the ongoing trial design on the premise of ensuring the integrity of the trial.

## 4.8 Study Termination

EOS is defined as the last patient last visit at the end of the follow-up period, which will be the last data collection point and the last patient may be an outpatient or laboratory sample. EOS is expected to occur approximately 2 years after enrollment of the last patient. A patient will be considered to have completed the study if he/she dies or meets any of the withdrawal criteria.

# 5 Study Population

Eligible patients will be screened within 28 days prior to the administration of the study drugs. Refer to Section 5.5, Screen Failures, for conditions that allow any screening procedure to be repeated.

The inclusion and exclusion criteria for patients enrolled in this study are summarized below. If there is any doubt about these criteria, the investigator must consult with the Sponsor representative and resolve all issues prior to study enrollment. No exemptions are allowed.

## 5.1 Eligibility Criteria for Prescreening

1. Sign pre-screening informed consent form (ICF).

2. Willing to provide a tumor tissue sample (archived or recently collected) for tumor genetic analysis.

## 5.2 Inclusion and Exclusion Criteria

### 5.2.1 Inclusion Criteria

1. Patients who fulfill the diagnostic criteria for T-LGLL or CLPD-NK specified in the 2016 WHO Classification of Lymphoid Neoplasms [1]

2. Patients are fully informed about the study and willing to participate in the study and provide written ICF

3. Male or female, aged ≥18 years

4. Treatment-naive patients, or patients treated with or relapsed from a non-methotrexate/ thalidomide-based regimen

5. Patients with at least one of the following indications for LGLL treatment:

    a. ANC <0.5 × $10^9$/L, or neutropenia with recurrent infections

    b. Hemoglobin <100 g/L, or requiring infusion of red blood cells

    c. Platelets level <50 × $10^9$/L

    d. Concomitant autoimmune diseases requiring treatment

    e. Symptomatic splenomegaly

    f. Severe B symptoms (fever of unknown cause with body temperature over 38 °C; night sweats; weight loss of ≥10% within half a year)

    g. Pulmonary hypertension

6. Patients with Eastern Cooperative Oncology Group (ECOG) score of 0 to 2 (Appendix 1)

7. Patients with life expectancy ≥6 months.

### 5.2.2 Exclusion Criteria

1. Patients not diagnosed with T-LGLL or CLPD-NK
2. Patients with no indication for LGLL treatment
3. Patients who are unable to understand or follow study procedures
4. Patients who have been diagnosed or treated for malignancies other than LGLL within the past five years
5. Patients with non-lymphoma-related hepatic and renal impairment: alanine aminotransferase (ALT) > 3 × upper limit of normal (ULN), aspartate aminotransferase (AST) >3 × ULN, total bilirubin > 2 × ULN, and serum creatinine clearance (CrCl) <30 mL/min
6. Patients with other severe diseases that have an impact on this study (uncontrolled diabetes, stomach ulcers, other severe cardiopulmonary conditions, etc.), at the discretion of investigators.
7. Patients with a known history of HIV infection, or with active hepatitis B virus (HBV) infection, or any uncontrolled active systemic infection requiring intravenous antibiotics

Notes: HBV infection is considered active if all following criteria are met: a. HBV DNA quantification ≥2000 IU/mL; b. ALT ≥2 times × ULN; c. LGLL, medications, and other causes-induced hepatitis are excluded. Patients with active HBV infection at the time of initial diagnosis who are converted to inactive HBV infection after adequate anti-HBV treatment could be enrolled in this study.

8. Patients who have undergone major surgery (excluding lymph node biopsy) within the past 14 days or who are expected to undergo major surgery during study treatment
9. Pregnant or lactating women, and women of childbearing potential who have not taken contraceptive measures
10. Patients who are allergic to the agents or the ingredients

## 5.3 Withdrawal Criteria

1. Patients withdraw the ICFs
2. Patients experience PD or death
3. Patients experience intolerable toxicity that cannot be relieved after symptomatic treatment
4. Patients are unwilling to continue treatment
5. There was no clinical benefit for more than half a year
6. Delayed dosing for more than 4 weeks and patients are judged by investigators to be unsuitable for further dosing
7. Pregnancy

8. Lost to follow-up

9. Treatment should be discontinued based on the best interests of the patients at the investigator' discretion

10. Patients does not comply with the protocol, and the investigator deems it necessary to discontinue the treatment

11. The collaborator or regulatory authority notifies to end the clinical study.

If treatment discontinuation is due to any of the above reasons, the investigator must notify the Principal Investigator and complete the case summary page in the case report form (CRF), along with the date and reason for the treatment discontinuation. If treatment discontinuation is due to an AE, the AE will be followed up until it is resolved and stable.

## 5.4 Removal of Patients from Therapy or Assessment

Efforts must be made to complete the efficacy and safety tests at the time of treatment discontinuation as specified in the protocol, and to complete safety follow-up, with complete documentation of AEs and outcomes. The investigator may recommend or provide new or alternative treatments to the patient according to the actual conditions. Patients with non-PD should continue to be followed up for efficacy evaluation until they start a new antineoplastic therapy or have PD.

If a patient refuses to visit the study site for further visits, he/she should continue to be followed up for the collection of information about the study unless he/she withdraws consent to disclose further information or be contacted. In this case, no further evaluation should be conducted and no further information should be collected.

## 5.5 Identification Codes, Enrollment, and Screening Log of Screening Failed Patients

Patients who meet the screening failure criteria may be rescreened. For abnormal screening values leading to exclusion during the screening period, it is allowed to retest (to reassess eligibility), and discuss with the Sponsor as appropriate. The last test result obtained prior to the first dose of the study drugs will be used to determine if a patient is eligible for the study. Measurements taken closest to the first dose of the study drugs (prior to the first dose) will be used as baseline values for safety assessments and treatment decisions.

The investigator will fill out the patient identification code and enrollment log to facilitate easy identification of each patient during and after completion of the study. The completeness of this document will be reviewed by the Sponsor's site contact. Patient identification codes and enrollment logs are confidential documents and will be kept by the investigator in the study documentation. To ensure the confidentiality of patient's information, this document should not be copied. For all study-related reports

and correspondences, patients will be identified by patient identification code and age on the initial ICF. If a patient is not enrolled in the study, the date when the patient is met and the age on the initial ICF will be used.

# 6 Treatments

## 6.1 Treatment Regimen

Treatment will be provided in 3-month cycles. Patients will receive thalidomide of 50 to 100 mg orally QD before bedtime, methotrexate of 10 mg/m$^2$ orally once weekly, and prednisone of 0.5 to 1 mg/kg orally QOD. Prednisone will be tapered after 12 months of dosing. Methotrexate will be dosed for up to 1 year. Thalidomide will be dosed for up to 2 years or discontinued at the investigator's discretion based on clinical symptoms. Efficacy was evaluated after 3 months of treatment, if PR or above response to treatment, the initial treatment regimen will be continued. If there is no clinical benefit after 6 months of initial treatment, patients will switch to the second-line treatment was and will be withdrawn from the study.

Dosage and administration for study drugs are shown in the following table:

| TPM Regimen | Dosage and Administration |
|---|---|
| Methotrexate | 10 mg/m$^2$ orally, once weekly |
| Prednisone | 0.5–1 mg/kg orally, every other day |
| Thalidomide | 50–100 mg orally, once daily before bedtime |

## 6.2 Administration of Study Drugs

**Thalidomide:**

Thalidomide administration will be started at 50 mg QD to observe whether there are adverse reactions such as rash and numbness of extremities, and then increased to 100 mg QD, if tolerated, for 1 week. Attention will be paid to observe adverse reactions such as peripheral neuritis. Thalidomide administration will be maintained for a total of 2 years and whether to discontinue will be determined based on the clinical symptoms. If the patient has a study drug-related adverse reaction, the dosage will be adjusted according to the dose adjustment guidelines in Section 7.4.1.

**Methotrexate:**

Methotrexate will be administered orally at 10 mg/m$^2$ once weekly on a fixed day in the morning. Patients who achieve CR can discontinue methotrexate after 4 months of consolidation therapy, with a maximum duration of 1 year.

**Prednisone:**

Prednisone will be administered orally at 0.5 to 1 mg/kg QOD for up to 6 months. It is recommended to take prednisone before 9:00 AM, with food or milk, to reduce stomach irritation. Take antacid between meals to help prevent peptic ulcer. The dose of oral prednisone will be tapered by 5 mg every 2 weeks after Months 3, and 5 mg every week after Month 4 until discontinuation.

## 6.3 Concomitant Medications and Supportive Care

### 6.3.1 Prophylactic Medications

Patients will be instructed not to take any additional medication (including over-the-counter drugs) during the study without consultation with the investigator. At each visit, the investigator will ask the patient about any new medication he/she is taking or has taken since the start of the study drug.

When combined with thalidomide, aspirin will be taken orally at 100 mg QD for antiplatelet therapy to prevent thrombosis.

For all patients for long-term use of low doses of methotrexate, folate supplementation (1 mg/day) is recommended to use to reduce the risk of some common methotrexate toxicities and the possibility of treatment discontinuation. The dosage of folate can be increased as needed to a maximum of 5 mg/day, depending on the reported residual symptoms. Folinate can be used to replace folate. Daily supplementation with folic acid or weekly supplementation with folinate can also prevent increases in plasma homocysteine concentration, which will be induced by methotrexate treatment without supplementation.

When combined with prednisone, calcium carbonate will be administered orally at 1 g QD and calcitriol will be administered orally at 0.25 μg twice daily to prevent osteoporosis; at the same time, proton pump inhibitor will be administered orally to inhibit gastric acid secretion and prevent gastrointestinal reactions.

Patients with positive hepatitis B surface antigen should receive routine examination of the HBV DNA copy number and be administered with anti-hepatitis B therapy (lamivudine or entecavir). Treatment in the active phase of the hepatitis B virus should be avoided. During treatment and within 6 months after treatment, lamivudine or entecavir should be administrated for prophylaxis, and hepatitis B DNA copy number quantification should be monitored during treatment (once every 1 to 2 months).

### 6.3.2 Permitted Medications

The investigator may prescribe any concomitant medication or therapy deemed necessary to provide adequate supportive treatment throughout the study, including those listed below:

Standard supportive treatment (antiemetics, antidiarrheals, anticholinergics, spasmodic drugs, antipyretics, antihistamines, analgesics, and other medications designed to treat the symptoms or signs) will be administered as clinically indicated according to local standard of care and when deemed necessary by the investigator.

- Growth factor support, erythropoietin, and blood product transfusion are allowed for the treatment of symptoms or signs of neutropenia, anemia, or thrombocytopenia according to local standard of care.
- Infectious complications with documented evidence should be treated with oral or intravenous antibiotics, or other anti-infective drugs that the investigator considers appropriate for the infectious condition according to local standards

of care. If clinically indicated, the investigator may administer prophylactic anti-infective therapy to patients according to local standards of care.

- Prevention of tumor lysis syndrome (TLS): For patients with elevated risk of TLS such as high tumor load and renal impairment, adequate hydration and alkalinization should be performed during treatment according to clinical practice, and blood chemistry should be closely monitored.
- Prevention and treatment of HBV activation.
- Other medications for concomitant diseases: Treatment for hypertension, diabetes, etc.
- If a patient experiences an AE during study treatment, the investigator should provide appropriate treatment according to clinical practice, including but not limited to antiemetics, hepatoprotective drugs, drugs for increasing leukocyte, transfusion/blood products, anti-infection drugs, and drugs causing QTc prolongation.

### 6.3.3 Prohibited Medications

The following medications are prohibited during the study. The clinical research associate (CRA) must be notified in advance (or as soon as possible thereafter) of any circumstances requiring prohibited or restricted therapy. In case of any queries or concerns about the use of any of the following prohibited or restricted medications, the investigator must consult the CRA for guidance.

- Any anti-cancer chemotherapy, immunotherapy, targeted therapy, experimental therapy, or hormone therapy (except for therapy with luteinizing hormone-releasing hormone). However, supportive therapeutic drugs, including bisphosphonates and hormone replacement drugs, are allowed.
- Hormonal contraceptives: Hormonal contraceptives can enhance the thrombotic effect of thalidomide.
- Azelastine (nasal): Thalidomide tablets can enhance azelastine’s depressant effect on the central nervous system.
- Ethanol: Concomitant administration with methotrexate tablets will increase the risk of drug reactions and liver toxicity, and long-term use may cause liver damage.
- Kanamycin: Concomitant administration with methotrexate tablets will affect its therapeutic effect and may increase the risk of other reactions, such as nausea, vomiting, diarrhea, and other gastrointestinal symptoms.
- Triamterene: Concomitant administration with methotrexate tablets will lead to increased serum concentration, increase toxicity and side effects, and also cause renal excretion disorders, resulting in kidney injury. Methotrexate tablets need to be administered under the guidance of the investigator.

- Carbonic anhydrase inhibitor: Long-term use of carbonic anhydrase inhibitor in combination with prednisone may lead to hypocalcemia, osteoporosis, etc. which should be avoided.
- COVID-19 vaccines (adenovirus vector, messenger RNA), vaccines (inactivated): The immune efficacy of these vaccines may be reduced.

## 6.4 Source and Use of Study Drugs

The study drugs in this study include thalidomide, methotrexate, and prednisone.

**Thalidomide:**

Commercially available thalidomide tablets will be used in this study. Each site will use commercially available thalidomide tablets for treatment according to clinical needs. Refer to local prescribing information for dosage form, packaging, and storage of thalidomide.

**Methotrexate:**

Commercially available methotrexate tablets will be used in this study. Each site will use commercially available methotrexate tablets according to clinical needs. Refer to local prescribing information for dosage form, packaging, and storage of methotrexate.

**Prednisone:**

Commercially available prednisone tablets will be used in this study. Each site will use commercially available prednisone according to clinical needs. Refer to local prescribing information for dosage form, packaging, and storage of prednisone.

# 7 Adverse Reactions and Dose Adjustment

## 7.1 Common Adverse Reactions of Thalidomide

**Teratogenic Effects:**

Thalidomide should not be used in pregnant women. When administered to pregnant women, thalidomide may cause fetal harm. Thalidomide is a highly potent human teratogen that induces severe and life-threatening birth defects at high frequencies, even with single dose. Approximately 40% of infants whose mothers have received this drug are reported to have died at birth or shortly after birth. Pregnant patients will not be enrolled in the study.

**Constipation:**

Administration of thalidomide may irritate the gastrointestinal tract, thus resulting in constipation.

**Nausea:**

Nausea ranks as the second most common adverse reaction in the digestive system, most nausea is mild to moderate in severity.

**Venous and Arterial Thromboembolism:**

Thalidomide may increase the risk of venous thromboembolism (eg, deep vein thrombosis and pulmonary embolism), which may be significantly increased when thalidomide is used in combination with standard chemotherapeutic agents including dexamethasone.

**Peripheral Neuropathy:**

Thalidomide may cause permanent nerve damage. Peripheral neuropathy is common (≥10%) and potentially irreversible with the serious adverse reaction to the treatment with thalidomide. Peripheral neuropathy usually develops after prolonged use over several months. However, peripheral neuropathy after relatively short-term use has also been reported. The correlation with cumulative dose is unknown. Symptoms may appear for some time after the discontinuation of treatment with thalidomide, and these symptoms may disappear slowly or completely.

**Neutropenia:**

White blood cell (WBC) count decreased with the clinical use of thalidomide, including neutropenia, have been reported.

**Thrombocytopenia:**

Grade 3 or 4 thrombocytopenia with the clinical use of thalidomide has been reported.

**Severe Skin Reactions**

Severe skin reactions have been reported for the treatment with thalidomide, including Stevens-Johnson syndrome (SJS), toxic epidermal necrolysis (TEN), and drug reaction

with eosinophilia and systemic symptoms (DRESS). DRESS may result in skin reactions (eg, rash or exfoliative dermatitis), eosinophilia, fever, and/or lymphadenopathy with systemic complications, such as hepatitis, nephritis, pneumonia, myocarditis, and/or pericarditis.

**Allergy**

Hypersensitivity reactions, including angioedema and allergic reactions to thalidomide, have been reported. Signs and symptoms include macular rash, which may be associated with fever, tachycardia, and hypotension.

**Seizure**

Although no premarketing controlled clinical trials have been reported, seizures, including severe convulsions, have been reported during the approval of thalidomide in clinical practice.

**Tumor Lysis Syndrome**

Monitor patients at risk for TLS (eg, patients with high tumor load before treatment)

**HIV Viral Load Increased**

In a randomized, placebo-controlled trial of thalidomide in HIV seropositive patients, increased plasma HIV RNA levels were observed with the median change from placebo of 0.42 log10 copies of HIV RNA/mL ($p = 0.04$). A similar trend was observed in another unpublished study of HIV seropositive patients. The clinical significance of this increase is unknown. Both studies were conducted before the implementation of highly active antiretroviral therapy.

**Bradycardia**

Bradycardia has been reported to be associated with the use of thalidomide. There are reported cases of bradycardia, some requiring medical intervention. The clinical significance and underlying etiology of bradycardia found in some patients treated with thalidomide are unknown.

**Somnolence**

Thalidomide often causes somnolence. Patients should be advised to avoid somnolence and not to take other medications that may cause somnolence without medical advice.

## 7.2 Common Adverse Reactions of Methotrexate

Methotrexate, an organic compound, is mainly used as an antifolate antineoplastic drug, which can inhibit dihydrofolate reductase, thereby impeding synthesis in tumor cells and inhibiting the growth and proliferation. Refer to the locally approved labeling (eg, refer to local prescribing information) for details of methotrexate. In this study, low-dose methotrexate is administered orally at 10 mg/m$^2$, once a week. The most common adverse reactions of low-dose methotrexate are summarized below.

**Elevated Transaminase**

Elevated transaminase is the most common adverse reaction of methotrexate and also the most common non-hematological AE, which is usually mild in severity. Liver function tests should be performed regularly after administration. Although these mild AEs will not lead to the discontinuation of methotrexate, this drug should be used with caution.

**Cytopenia**

In patients receiving low-dose methotrexate, cytopenia (neutropenia, thrombocytopenia, and anemia) is generally reported as Grade 1 or 2, but treatment-emergent Grade 3 or 4 cytopenia (neutropenia, thrombocytopenia, and anemia) is also reported. Anemia is the most common hematological adverse reaction, followed by neutropenia and thrombocytopenia.

**Nausea and vomiting**

Nausea and vomiting are another common non-hematological AEs. Other frequently reported gastrointestinal events included diarrhea, abdominal pain, cholecystitis, and gallstones. These events are rarely serious. If symptoms are severe or persist for a long time, follow the guidelines for dose adjustment in the protocol.

**Stomatitis**

Stomatitis has an incidence of < 10% and is generally of Grade 1 or 2. Symptoms may improve after symptomatic treatment.

**Rash**

Rash can be manifested as erythematous rash, alopecia, pruritus, redness and swelling, with an incidence of <3%. There are rare case reports of severe skin reactions, including toxic epidermal necrolysis, Stevens-Johnson syndrome, exfoliative dermatitis, cutaneous necrosis, and erythema multiforme.

## 7.3 Common Adverse Reactions of Prednisone

Prednisone is a glucocorticoid with anti-inflammatory, anti-allergic, antirheumatic and immunosuppressive effects. It is a commonly used medication for hematologic disorders. Due to its frequent clinical use, details will not be provided in this protocol.

**Water-electrolyte Disturbance**

Water-electrolyte disturbance includes sodium retention, fluid retention, congestive heart failure in susceptible patients, potassium loss, hypokalemic alkalosis, and hypertension.

**Musculoskeletal System**

Musculoskeletal system events include myasthenia, steroid myopathy, muscle tissue loss, osteoporosis, tendon rupture (especially Achilles's tendon rupture), vertebral compression fracture, aseptic necrosis of femoral and humeral heads, and pathological fracture of diseased long bone.

**Digestive System**

Digestive system events include ulcer (possible perforation and bleeding), pancreatitis, abdominal distension, and ulcerative esophagitis.

**Skin/Subcutaneous Tissue**

Difficult-to-heal wounds, thin fragile skin, petechiae and ecchymosis, facial erythema, and increased sweating may suppress reactions to skin tests.

**Metabolic System**

Metabolic system event includes negative nitrogen balance caused by protein catabolism.

**Nervous System**

Elevated intracranial pressure, vertigo and headache with papilledema (pseudoencephaloma) usually occur after treatment.

**Endocrine System**

Endocrine system events include irregular menstruation, development of Cushingoid syndrome, secondary adrenocortical and pituitary unresponsiveness (especially in stressful states such as trauma, surgery or disease), inhibition of growth and development in children, reduced carbohydrate tolerance, manifestations of latent diabetes, increased demand for insulin or oral hypoglycemic agents in diabetic patients.

**Eyes**

Eyes events include posterior subcapsular cataract, elevated intraocular pressure, glaucoma, and proptosis (exophthalmos).

**Anaphylaxis**

Anaphylaxis events include urticaria and other allergy, anaphylaxis or hypersensitivity.

## 7.4 Guidelines for Dose Adjustment and Discontinuation

In the event of toxicity, the investigator should accurately assess the severity of AEs and serious adverse events (SAEs) based on the patient’s condition and the National Cancer Institute (NCI) Common Terminology Criteria for Adverse Events (CTCAE, Version 5.0). Appropriate medication (including infusions, cytokine growth factors, antiemetics, antidiarrheals, etc.) should be administered to treatment AEs. All AEs will be assessed from the time of the first dose of the study treatment until the final study visit. Patients who have experienced AEs may be followed up after the EOS visit for additional assessments until the AE resolves or is deemed stable.

For patients who are unable to tolerate the protocol-specified administration regimen, treatment discontinuation or dose adjustments are allowed. Before treatment discontinuation and dose adjustment, the attribution of AEs to the 3 drugs (thalidomide, methotrexate, and prednisone) should be determined. For non-hematological toxicities attributable to methotrexate and prednisone (not thalidomide), administration of thalidomide may be continued.

### 7.4.1 Dose Adjustment and Discontinuation of Thalidomide

If thalidomide-related peripheral neuropathy occurs, neuroprotective drugs can be used as appropriate; if there are ≥ Grade 2 or Grade 1 side effects with pain, reduce the dose by 1 dose level and discontinue in those without improvement. See Appendix 3 for details of thalidomide neurotoxicity grading and dose adjustment.

### 7.4.2 Dose Adjustment and Discontinuation of Methotrexate

Methotrexate is contraindicated in LGLL patients with an estimated glomerular filtration rate (eGFR) <30 mL/min. For patients with an eGFR of 30 to 59 mL/min, a lower initial dosage is required; and a more gradual dose increase, close monitoring, and a lower maximum weekly dose should be considered, depending on the overall clinical conditions. Although low doses of methotrexate are not nephrotoxic, methotrexate is almost completely excreted through the kidney. Therefore, kidney function should be monitored. If renal insufficiency occurs, the methotrexate dose should be reduced or discontinued.

Guidelines from Cancer Care Ontario in Canada recommend a 25% reduction of dose at CrCl of 80 mL/min, 40% reduction at CrCl of 60 mL/min, 50% reduction at CrCl of 50 mL/min, and discontinuation at CrCl <50 mL/min. The guidelines also state that less conservative dose adjustments may be considered when using a low-dose regimen (<50 $mg/m^2$). High-dose methotrexate could only be administered only when CrCl is >60 mL/min.

See Appendix 2 for the calculation method of CrCl.

### 7.4.3 Dose Adjustment and Discontinuation of Prednisone

The initial dose of prednisone is 0.5 mg/kg QOD, which should be adjusted according to the hematologic index. Actively monitor blood pressure and blood glucose, and observe adverse reactions of glucocorticoids. If there are adverse reactions of Grade 1 or 2, the dose can be reduced. If adverse reactions with ≥ Grade 3 occur, dose can be gradually reduced and eventually discontinued. If steroid-induced acute psychotic symptoms occur and antipsychotics are ineffective; or if corneal ulcers caused by the herpes virus occur, which can quickly lead to corneal perforation and may result in permanent blindness, discontinue the use of prednisone immediately or rapidly reduce the dosage significantly, instead of reducing it gradually.

## 7.5 Other Treatment Considerations

During the patient's treatment, if unrelated illnesses or accidents occur, and there is a need to temporarily stop the treatment, it should be decided in consultation with the investigator.

# 8. Study Procedures

Only untreated or standard-of-care naive patients, patients with poor responses (failure to achieve PR) or relapse after receiving a non-methotrexate/thalidomide-based regimen, and patients who did not achieve PR within 4 weeks of methotrexate monotherapy can be enrolled in this study. Therefore, prior treatment and efficacy will also be evaluated before enrollment. The study consists of a screening period (Day −28 to Day 0), a treatment period, end of treatment, and a follow-up period.

## 8.1 Screening Period

All patients will undergo study eligibility screening, which will be completed within 4 weeks before the start of the study, including:

- Review of inclusion/exclusion criteria
- Physical examinations
- Vital signs measurements (body temperature, pulse, systolic and diastolic blood pressure, and respiratory rate)
- Height, weight, and body surface area
- ECOG performance status
- Disease-related signs and symptoms
- Assessment and recording of serious pre-treatment events
- Complete medical history (previous treatment and efficacy, enlargement of liver, spleen and lymph nodes; presence of other comorbidities)
- Hematology: Hemoglobin, ANC, WBC count, platelets
- Blood chemistry: Serum glucose, AST, ALT, total bilirubin, creatinine, Na, K, uric acid, total protein, and albumin
- Serum lactate dehydrogenase (LDH)
- Serum β2 microglobulin
- Serum immunoglobulin quantification
- Serological tests for HIV, hepatitis C virus (HCV), and HBV (including hepatitis B surface antigen [HBsAg], anti- hepatitis B surface antibody [HBsAb], and anti-hepatitis B core antibody [HBcAb])
- HBV-DNA and HCV RNA: HBV-DNA and HCV RNA will be used in patients with serologically positive HBV or HCV, respectively
- Coagulation function (8 items)
- Detection of peripheral blood lymphocyte subsets (B/T/NK; Treg; T-cell immune function; TH1/TH2)

- Peripheral blood flow cytometry
- Pregnancy test (if applicable)
- Urinalysis and stool
- Bone marrow aspiration and biopsy
- Bone marrow smear, immunohistochemistry, and flow immunophenotyping
- Flow cytometry detection of LGLs in patient's bone marrow fluid or peripheral blood; flow cytometry detection of killer immunoglobulin-like receptor; TCR rearrangement; TCRvβ flow cytometry, karyotyping; next generation sequencing
- Electrocardiograph (ECG)
- Computed tomography (CT) for assessment of measurable lesions (including neck, chest, abdomen, and pelvis)
- Assessment of cardiac function and ejection fraction by two-dimensional echocardiography; abdominal color Doppler ultrasound, etc.

Other test options:

Infection-related markers; fluorescence in situ immunohybridization (17p13); complete test of viruses, hemolysis-related tests (if relevant clinical manifestations are present): Coombs test and cold agglutinin test; rheumatoid factor, complement, ENA antibody spectrum, antinuclear antibody, antiplatelet antibody (when immune thrombocytopenia is suspected), antiphospholipid antibody (when combined with thrombus), serum ferritin plus serum iron test.

## 8.2 Treatment Period

Collect, record and report patient information and data in a timely manner to ensure that the data and information are timely, consistent, complete, reliable, and accurate. AEs, especially SAEs, should be treated in a timely and proper manner, timely tracked, followed up, recorded, and reported. Original records should be kept.

**Months 1 to 24 of TPM Treatment**

- Physical examination and vital sign measurements
- ECOG performance status
- Assessment and recording of AEs to further assess whether the treatment can be continued
- Detection of LGLs in peripheral blood
- Hematology: Hemoglobin, ANC, WBC count, platelets.
- Blood chemistry: Serum glucose, AST, ALT, total bilirubin, creatinine, uric acid, etc.

**At the End of Cycle 1 (Every 3 months as 1 cycle)**

- Physical examination and basic vital sign measurements
- ECOG performance status
- Assessment and recording of AEs to further assess whether the treatment can be continued
- Hematology: Hemoglobin, ANC, WBC count, platelets
- Blood chemistry: Serum glucose, AST, ALT, total bilirubin, creatinine, uric acid, LDH, etc.
- Detection of LGLs in peripheral blood
- Detection of peripheral blood lymphocyte subsets (B/T/NK; Treg; T-cell immune function; TH1/TH2)
- Peripheral blood flow cytometry
- Peripheral blood TCRvβ flow cytometry
- Bone marrow smear, bone marrow biopsy, and immunohistochemistry
- Chromosome karyotype examination in bone marrow or peripheral blood
- ECG
- Evaluation of measurable lesions (including neck, chest, abdomen, and pelvis) by CT in patients with lymph node enlargement before admission
- Assessment of cardiac function and ejection fraction by two-dimensional echocardiography; abdominal color Doppler ultrasound, etc.
- Coagulation function

**At the End of Cycles 2 to 8 (Every 3 months as 1 cycle)**

- Physical examination and vital sign measurements
- ECOG performance status
- Assessment and recording of AEs to further assess whether the treatment can be continued
- Hematology: Hemoglobin, ANC, WBC count, platelets
- Blood chemistry: Serum glucose, AST, ALT, total bilirubin, creatinine, uric acid, LDH, etc.
- Detection of LGLs in peripheral blood
- Detection of peripheral blood lymphocyte subsets (B/T/NK; Treg; T-cell immune function; TH1/TH2)
- Peripheral blood flow cytometry
- Peripheral blood TCRvβ flow cytometry

- ECG
- Evaluation of measurable lesions (including neck, chest, abdomen, and pelvis) by CT in patients with lymph node enlargement before admission
- Assessment of cardiac function and ejection fraction by two-dimensional echocardiography; abdominal color Doppler ultrasound, etc.
- Coagulation function

### 8.2.1 Disease Progression

The specific tests are the same as those in the screening period.

## 8.3 List of Tests and Key Time Points

| Test | Screening/ Recurrence Progression | Post-Cycle 1 | Every 3 Months Thereafter |
|---|---|---|---|
| Hematology | √ | √ | √ |
| Blood type | √ | | |
| HIV/syphilis/hepatitis | √ | | |
| Complete test of viruses | √ | | |
| Lymphocyte subsets (at Blood Research Institute) | √ | √ | √ |
| All items of cytokines (at Blood Research Institute is available) | √ | √ | √ |
| Detection of peripheral blood large granular lymphocytes | √ | √ | √ |
| TCRvβ flow cytometry | √ | √ | √ |
| Immunoglobulin quantification | √ | | |
| Hepatorenal and cardiac function, blood glucose | √ | √ | √ |
| Electrolytes | √ | √ | √ |
| Lactate dehydrogenase | √ | | |
| Blood β2-MG | √ | | |
| Coagulation (8 items) | √ | √ | √ |
| Classification of bone marrow smears | √ | When necessary | When necessary |
| Bone marrow biopsy (paraffin-embedded) | √ | When necessary | When necessary |
| Flow immunophenotyping | √ | When necessary | When necessary |
| Karyotype | √ | When necessary | When necessary |
| TCR β、TCRγ、TCRδ | √ | When necessary | When necessary |
| FISH | When necessary | When necessary | When necessary |
| Second-generation genetic testing | √ | | |
| Electrocardiograph | √ | √ | √ |
| Cardiac color Doppler ultrasound (cardiac function) | √ | √ | √ |
| B-scan ultrasonography (superficial lymph nodes) | √ | √ | √ |
| Cervical/chest/abdominal computed tomography | √ | When necessary | When necessary |
| Test of infection foci | When necessary | When necessary | When necessary |

## 8.4 Early Withdrawal (Discontinuation of Study Treatment)

**Three months after the Last Treatment:**

- Physical examinations
- Vital signs measurements (body temperature, pulse, systolic and diastolic blood pressure, and respiratory rate)
- ECOG performance status
- Disease-related signs and symptoms
- Assessment and recording of AEs
- Hematology: Hemoglobin, ANC, WBC count, platelets
- Blood chemistry: Serum glucose, AST, ALT, total bilirubin, creatinine, Na, K, uric acid, total protein, and albumin
- Serum LDH
- Serum immunoglobulin quantification
- Urinalysis and stool
- Detection of LGLs in peripheral blood
- Detection of peripheral blood lymphocyte subsets (B/T/NK; Treg; T-cell immune function; TH1/TH2)
- Peripheral blood flow cytometry
- Peripheral blood TCRvβ flow cytometry
- Bone marrow smear, bone marrow biopsy, and immunohistochemistry
- Chromosome karyotype examination in bone marrow or peripheral blood
- ECG
- Evaluation of measurable lesions (including neck, chest, abdomen, and pelvis) by CT in patients with lymph node enlargement before admission
- Assessment of cardiac function and ejection fraction by two-dimensional echocardiography; abdominal color Doppler ultrasound, etc.
- Coagulation function

Following this assessment, patients will be followed up twice a year until the EOS, including survival, disease status, and long-term toxicity.

## 8.5 Follow-up

### 8.5.1 Survival Follow-up

Survival follow-up will be performed after the end-of-treatment visit (including patients who discontinue treatment early due to intolerance). Patients will be re-examined every

6 months and followed up for 2 years from the end of treatment.

### 8.5.2 Safety Follow-up

Safety follow-up will be performed for all patients who complete or discontinue treatment until 1 year after the last dose of the study drug, or until initiation of a new antineoplastic therapy (whichever occurs first).

Patients will be contacted by phone 1 year after the last dose, or asked to the study site for a safety visit to collect the ongoing AEs or changes in laboratory abnormalities, and to assess new AEs, SAEs, new concomitant medications, or changes in current medications since the last assessment. Any new conditions that occur during the safety follow-up need to be documented in the CRF as appropriate. After the safety follow-up (ie, 28 days after the last dose), there is no need to actively collect AEs. However, if an SAE occurs and the investigator assesses a reasonable causality to the study drug, the SAE should be reported.

AEs (regardless of causality) that do not completely resolve at the end of safety follow-up must be followed up until recovery (chronic, baseline value, or completely recovery), or clinical stability is achieved.

# 9. Efficacy Evaluation

## 9.1 Definition

### 9.1.1 General Definition

Evaluable toxicity: All patients who have received at least 1 month of TPM treatment are evaluable for toxicity evaluation from the first treatment.

Evaluable objective response: Only patients who have a measurable disease at baseline, have received at least 1 treatment cycle, and have been re-evaluated for the disease will be considered as evaluable responses. These patients will be categorized for their responses according to the following definitions (Note: Patients who demonstrate objective PD or death by the end of Cycle 1 will also be considered evaluable).

## 9.2 Efficacy Evaluation

The evaluation will be started from Month 3. Hematologic ORR is defined as the sum of CR and PR.

Hematologic CR: Clinical symptoms and signs turn normal (splenomegaly disappears), and blood cell count completed turn normal, ie, hemoglobin >110 g/L, platelets >100 × $10^9$/L, ANC >1.5 × $10^9$/L, lymphocyte count <4 × $10^9$/L, and LGLs in peripheral blood are within the normal range (<0.5 × $10^9$/L).

Complete molecular response: No LGLs can be detected when reaching CR (negative TCR rearrangement by flow cytometry or polymerase chain reaction [PCR]).

Hematologic PR: Hematologic PR is defined as blood cell count that do not meet criteria for CR but improvement is observed, such as neutrophil counts >0.5 × $10^9$/L without repeated infection, an increase of hemoglobin level from baseline by more than 20 g/L without blood transfusion, platelets >50 × $10^9$/L.

SD: CR/PR criteria are not met after standardized treatment for 4 months, but no significant progression is observed.

PD: Worsening of hematologic parameters in patients previously achieving PR/CR.

PFS: PFS is defined as the time from the first day of study to PD or death, including PD and death from any cause (eg, PD, toxicity, unrecorded progression in a new treatment, or death).

DoR: DoR is defined as the time when CR or PR is met until the first date of recurrent or progressive disease.

TTNT: TTNT is defined as the time between the start of TPM regimen and the start of the subsequent treatment line.

## 9.3 Study Endpoints Definition and Evaluation

**Primary Endpoint:**

- CRR of patients with symptomatic LGLL treated with thalidomide, prednisone, and methotrexate.

**Secondary Endpoints**

**1. Secondary Safety Endpoint**

- Incidence and severity of hematological and non-hematological AEs

Safety endpoints include SAEs, treatment-emergent adverse events (TEAEs), physical examination (including ECOG performance status), vital sign measurements, standard clinical laboratory parameters, and ECG. TEAEs will be graded according to NCI CTCAE Version 5.0.

**2. Secondary Efficacy Endpoints**

- Response rates: ORR and PR rate (time frame: 24 months after the last treatment)

Point estimates of rates will be calculated for each protocol analysis set. An estimate of the 95% CI for the response rate will also be derived. Graphical and descriptive analyses will be used to explore associations between relevant markers and responses.

- PFS and OS (time frame: 24 months after the last treatment)

Time from the first day to PD or death from any reason. The median duration of the overall response will be assessed with a 95% CI.

- DoR (time frame: 24 months after the last treatment)

The time when CR or PR is met until the first date of recurrent or progressive disease. The median duration of the overall response will be assessed with a 95% CI.

- TTNT (time frame: 24 months after the last treatment)
- Effect of biomarkers on efficacy and adverse reactions

**Exploratory Endpoint:**

- To explore biomarkers significantly and potentially associated with clinical efficacy

# 10. Safety Evaluation

Timely, accurate, and complete reporting and analysis of safety information from clinical studies are essential to protect patients and investigators and are mandatory by regulatory authorities worldwide. Each AE verbatim term will be coded to a system organ class and preferred term using the Medical Dictionary for Regulatory Activities (MedDRA).

## 10.1 Definition and Classification of Adverse Events

**Adverse Events:**

An AE is any untoward medical occurrence in a patient to whom a medicinal product is administered, and which does not necessarily have a causal relationship with this treatment. An AE can therefore be any unfavorable and unintended sign (eg, an abnormal laboratory finding), symptom, or disease temporally associated with the use of a medicinal product, whether considered related to this medicinal product. (As defined by the International Council for Harmonisation of Technical Requirements for Pharmaceuticals for Human Use [ICH]).

It includes any new event, any event worsened from baseline in severity or frequency, or abnormal results of diagnostic procedures, including laboratory test abnormalities.

**Serious Adverse Events:**

An SAE, as defined by ICH, as an AE that fulfils one or more of the following criteria at any dose:

- Results in death
- Is life-threatening ((life-threatening refers to a reaction in which the patient was at risk of death at the time of the reaction; it does not refer to a reaction that hypothetically might have caused death if more severe).)
- Requires hospitalization or prolongation of existing hospitalization
- Results in persistent disability/incapacity (The term disability means a substantial disruption of a person's ability to conduct normal life functions.)
- Is a congenital anomaly/birth defect
- Is an important medical event

Note: Medical or scientific judgment should be exercised in deciding whether SAE reporting is appropriate in other situations such as important medical events that may not be immediately life-threatening or result in death or hospitalization but may jeopardize the patient or may require medical or surgical intervention to prevent one of the other outcomes listed in the above definition. These events should usually be considered serious.

Events that are not considered SAEs include planned hospitalization before enrollment

into the clinical study; a disease for elective treatment and unrelated to the indication under study or its treatment; events occurring in an emergency outpatient department and not leading to hospitalization (unless other criteria above are met); part of normal treatment or monitoring of the study indication and unrelated to condition worsening.

If an AE is considered serious, the AE page in the CRF and the SAE Report Form must be completed. The investigator will provide information on severity, start and end dates, relationship to the study drug, actions taken with the study drug, and outcomes.

**Suspected Unexpected Serious Adverse Reaction:**

For unexpected AEs, their nature or severity is inconsistent with the that of the study drug. For the study drug, the expectedness of an AE will depend on whether it is listed in the Investigator's Brochure of the study drug. For a drug with marketing authorization, the expectedness of an AE will depend on whether it is listed in the summary of product characteristics (SmPC).

**Severity Assessment:**

For AEs and SAEs, the investigator must assess the severity of the events. According to the NCI CTCAE Version 4.03, the severity of AEs will be graded from 1 to 5. A copy of the NCI CTCAE Version 5.0 can be downloaded from the homepage of the Cancer Therapy Evaluation Program (CTEP) (https://ctep.cancer.gov/protocolDevelopment/electronic_applications/docs/CTCAE_v5_Quick_Reference_5x7.pdf). In those cases where NCI CTCAE is not applicable, the severity should be defined according to the following criteria:

Grade Definition:

Grade 1 (mild AE): asymptomatic or mild symptoms; usually transient, not requiring treatment, and generally not interfering with daily activities

Grade 2 (moderate AE): Moderate discomfort, interfering with daily activities; usually relieved by basic treatment

Grade 3 (severe AE): Severe incapacitation, inability to perform daily activities, or significantly affecting clinical status, requiring intervention. Hospitalization may or may not be required.

Grade 4 (life-threatening or disabling AE): Life-threatening consequences, life-threatening; requiring hospitalization and clinical intervention.

Grade 5 (AE-related death): Death related to AE

**Causality Assessment:**

The investigator must determine that the relationship between the administration of the study drug and AEs/SAEs as defined below:

- Unrelated. AEs are not related to the use of the study drug.

- Unlikely/suspicious. AEs that are more likely to have alternative explanations, such as concomitant medications, concomitant diseases, or temporal relationships suggest little causality.
- Possibly. Treatment with the study drug caused or contributed to the AE. Alternative explanations, such as concomitant medications and diseases, are uncertain. The event follows a reasonable temporal sequence from the time of drug administration; therefore, causality cannot be ruled out.
- Probably. AEs are probably caused by the use of the study drug. A reasonable temporal sequence of the event with drug administration exists Alternative explanations don't make sense, such as concomitant medications and concomitant diseases.
- Definitely. AEs that are listed as possible AE reactions and cannot be reasonably explained by alternative explanations, such as concomitant medications and concomitant diseases. The relationship in time is very suggestive (eg, it is challenged and re-challenged).
- Non-evaluable: The evidence for making a clinical judgment of causality is insufficient or incomplete.

**Treatment-related Mortality:**

If an AE related to the study drug is considered to result in the death, that event will be listed as "treatment-related mortality".

## 10.2 Safety Evaluation

If a patient completes at least 1 month of effective treatment, the patient will be analyzed for safety. The tolerability of treatment will be assessed by evaluating laboratory parameters and AEs. The incidence of all AEs during the treatment and up to 30 days after the last drug administration will be recorded.

AEs will be classified by severity, duration, and frequency. Details of AEs will be listed by patients, including time of onset, duration, toxicity grade, corrective treatment, outcome, and relationship to the investigational product. Safety parameters will be assessed by 2 independent investigators who will assess severity and treatment each time.

Study drug should be discontinued for any uncontrolled non-hematological toxicity that may be associated with the study drug and of Grade 3 or higher, and for any hematological toxicity that meets the criteria. For patients requiring full-dose anticoagulation (eg, heparin), the study drug should be interrupted until the anticoagulation is stable. For patients requiring an invasive procedure or surgery, the study drug must be interrupted. Any other clinically important events, in which the investigator may deem appropriate dose delays, must be discussed with the CRA. Details of drug adjustment and discontinuation criteria, refer to Section 7.4.

## 10.3 Recording of Adverse Events

### Diagnosis/Pathogenic Events and Signs and Symptoms

All AEs and SAEs should be reported. If known, the diagnosis should be recorded on the CRF rather than individual sign and symptom. However, if a series of signs and/or symptoms cannot be medically characterized as a single syndrome at the time of reporting, individual sign and/or symptom should be recorded on the CRF as AEs or SAEs. If a serious of signs and/or symptoms can be medically characterized as a single syndrome (eg, nausea and vomiting), the most medically significant sign and/or symptom should be reported as an AE, and other signs and/or symptoms should be recorded in the Additional Case Details of the CRF. If a diagnosis is subsequently confirmed, the event term should be updated to reflect the medical diagnosis.

In general, AEs secondary to other events (eg, cascade events or clinical sequelae) should be identified by the primary cause (pathogenic event) and additional sequelae should be recorded in the Additional Case Details of the CRF.

However, AEs that are temporally separate or secondary to an initial event of medical significance should be recorded on the CRF as independent events.

### Persistent or Recurrent Adverse Events

A persistent AE is an ongoing extension with no resolution between assessment time points. Such events should be recorded only once on the CRF unless the severity increases. If persistent AEs become more severe, they should be recorded again on the AE page of the CRF.

Recurrent AEs are those that occur and resolve between assessment time points and subsequently recur. All recurrent AEs should be recorded on the AE page of the CRF.

### Laboratory Abnormalities

Only clinically significant laboratory abnormalities requiring active management are recorded as AE or SAE on the CRF. The criteria for clinical significance are as follows:

- Laboratory abnormalities with clinical symptoms
- Laboratory abnormalities requiring dose adjustment, interruption, or termination of the study drug
- Laboratory abnormalities requiring more frequent follow-up assessments and further diagnostic investigations, etc.
- Laboratory abnormalities requiring changes in concomitant medication, therapy, or treatment

If a clinically significant laboratory abnormality is a symptom of disease or syndrome (eg, alkaline phosphatase and bilirubin are 5 × ULN associated with cholecystitis), only the diagnosis (eg, cholecystitis) needs to be recorded on the AE page of the CRF.

If a clinically significant laboratory abnormality is not a symptom of disease or syndrome, the abnormality itself should be recorded on the CRF as an AE or SAE. Observations of the same clinically significant laboratory abnormalities at each visit should not be repeatedly recorded as AEs or SAEs on the CRF unless the severity or etiology changes.

**Number of Deaths**

Deaths that occur during the protocol-specified AE reporting period and are attributed solely to progression by the investigator will be recorded only on the CRF. Other deaths, regardless of attribution, will be recorded on the CRF and promptly reported to the investigator.

## 10.4 Reporting of Adverse Events

All AEs and SAEs that occur between the first study-related procedures and within 30 days after the last dose of the study drug will be reported. All AEs, regardless of severity or presumed relationship to the study drug, must be recorded on the CRF using medical terms. Refer to (https://ctep.cancer.gov/protocolDevelopment/electronic_applications/docs/CTCAE_v5_Quick_Reference_5x7.pdf) for details. The investigators also need to record their opinions on the relationship between AEs and the study drug on the CRF. All actions required for AE management must be recorded in the source documents. All SAE reports must be submitted within 24 hours. For initial SAEs, all case details should be collected within 24 hours. The initial report must be as complete as possible, including details of the current diseases and SAEs, as well as an assessment of the causality between the event and the study drug. Information that is not available at the time of initial reporting (eg, end date or laboratory values of AEs) must be recorded on subsequent SAE forms when available and/or immediately upon request. The investigator must keep a copy of all SAE information on file. All SAEs that are unresolved at the time a patient discontinues study must be followed until the event is fully resolved, stabilized/sequelae resolved, or baseline (if any) is returned.

## 10.5 Data Safety

Each site shall complete the CRF and adverse reaction form or provide pictures according to the requirements of the CRF, and submit to the leading site (Institute of Hematology and Blood Diseases Hospital, Chinese Academy of Medical Sciences) for data entry and management after being reviewed by investigators. All process should be recorded.

Two independent site personnel should be responsible for data entry and management at each site. To ensure the accuracy of data, two statisticians independently enter them in duplicate and check. For queries about the CRF, the data statistician will fill in the Data Request Order (DRO) and send it to the investigator, who should promptly respond and return the DRO. Based on the investigator’s response, the data statistician

will make necessary data modifications, confirmations, and entries, and send a DRO again if necessary.

A Data Safety Monitoring Committee (DSMC) consisting of at least 4 independent members (2 clinicians and 2 independent statisticians) will be established for this study. The DSMC will meet regularly to review safety and efficacy data from trials prepared by independent statisticians, and the clinician will perform interim analyses. All data presented at the meeting will be kept confidential. After each meeting, the DSMC will prepare a report and may recommend changes to the protocol.

# 11. Statistical Analysis

## 11.1 General Consideration

The statistical analyses of this study will be conducted using the statistical analysis software SAS 9.4.

Quantitative data will be statistically described using mean, standard deviation, median, maximum, and minimum. Enumeration data or grade data are expressed in terms of the number and percentage of patients.

1. Describe the completion of the trial, and statistically describe the excluded and dropout patients one by one.

2. Describe demographics and other baseline parameters.

Efficacy analysis: Summarize the data with descriptive statistics, and the main indicator is the response rate of patients, including ORR, CRR, and PR rate. The $\chi^2$ test is used for the overall comparison, while the $\chi^2$ partitioning method is used to correct α for pairwise inter-group comparisons. Continuous variables will be presented as number, mean, standard deviation, median, and range. Discrete variables will be summarized as frequency. For time-to-event variables such as DoR and TTNT, the Kaplan-Meier method is used to estimate the event occurrence rate and plot the curve. The comparison of the curves is carried out using the Log-rank method. The Cox regression model is used to analyze the influencing factors of event occurrence.

Comparisons between the 2 treatment groups are as follows: for changes of continuous variables from baseline to a specific post-baseline time point, an analysis of variance (ANOVA) is used. For discrete variables, the Cochran-Mantel-Haenszel chi-square test is used. For variables of time to onset, a stratified log-rank test is used. Except for the analysis of primary indicators, the two-sided α level in other tests is 0.05. If pairwise comparisons between groups are involved.

## 11.2 Safety Analysis

The safety profile will be analyzed based on AEs, physical examination, vital sign measurements, laboratory measurements, and ECG findings. AEs will be graded as per NCI CTCAE (Version 5.0).

In general, the safety analysis will be descriptive and presented in tabular form with appropriate summary statistics. The number of patients and events of hematological and non-hematological toxicity (based on NCI CTCAE Version 5.0) will be listed, and the incidence will be calculated.

## 11.3 Efficacy Analysis

The efficacy analysis will involve CRR and ORR (sum of CR and PR rates). Efficacy variables will be tabulated and summarized. For the numbers and percentages of CRR

and ORR, point estimates and 95% exact binomial CIs will be provided.

Time-to-event variables including DoR, TTNT, PFS, and OS will be summarized descriptively using the Kaplan-Meier method. The rules for DoR, TTNT, PFS, and OS analyses will be specified in the Statistical Analysis Plan (SAP). Growth modulation indices (intra-subject ratios of DOR, PFS, OS, and TTNT post-study treatment versus DOR, PFS, OS, and TTNT post the most recent prior treatment regimen) will be summarized.

## 11.4 Biomarkers Exploratory Analysis

The exploratory analysis of biomarkers will be tabulated and summarized using descriptive statistics.

# 12. Quality Control and Assurance of Good Clinical Practice

## 12.1 Monitoring, Audit, and Inspection

Monitoring will be conducted mainly by email and phone during the study. The on-site CRA will visit the site when required, mainly in cases of data inconsistencies, to check the completeness of patient records, the accuracy of CRF entries, adherence to the protocol and good clinical practice (GCP) guidelines, and the status of enrollment. Key investigators must assist the on-site CRA during these visits.

The investigator must retain source documents for each patient in the study, including medical records and visit records (hospital or clinic medical records) containing demographic and medical information, laboratory data, ECG, and results of any other tests or assessments. All information on the CRF must be traceable to these source files in the patient file. The investigator must also retain the original signed ICF from patients (providing the patients with a signed copy).

The investigator must grant the CRA access to all relevant source documents to confirm the consistency with CRF entries. The safety monitoring standards require a thorough validation of the existence of informed consent, compliance with inclusion/exclusion criteria, documentation of SAEs, and data records to be used for all primary and safety variables. Additional checks for consistency of source data with the CRF are performed according to a study-specific monitoring plan. Information about the patient's identity will not be disclosed in source files.

## 12.2 Investigator's Responsibilities

The responsibilities of investigators are set out in the ICH GCP guidelines. The investigator must grant the CRA access to relevant records to confirm the above.

The investigator is responsible to record for all patients who have signed ICFs and have been screened for participation in the study. For patients who are excluded, reasons must be recorded in the patient's source documents.

No procedures/assessments/measurements/tests, other than those outlined herein or on the study evaluation schedule, can be performed without prior written approval from the Principal Investigator, or unless deemed necessary for the patient's medical care by the investigator. The investigator and/or authorized designee must enter study data into the provided CRFs. Data on the CRFs will be recorded anonymously to protect the identity of the patients by using unique identifiers that prevent the disclosure of personal identity information.

The investigator or designee must be available during the monitoring visit to review data and resolve any queries, as well as allow direct access to patient records (eg, medical records, office charts, hospital charts, and study-related charts) for source data validation. The CRFs must be completed as soon as possible after the patient's visit, but no later than the start of each monitoring visit, and provided to the representative for

checking accuracy and completeness.

# 13. Ethical and Regulatory Considerations

## 13.1 Review and Approval by Institutional Review Board/Independent Ethics Committee

This study will be conducted in accordance with the Declaration of Helsinki - Ethical Principles for Medical Research Involving Human Subjects (for more information, see: http://www.wma.net/e/policy/b3.html). The review by the Institutional Review Board/Independent Ethics Committee (IRB/IEC) of this study, as well as the conduct of the study and the methods used to obtain informed consent, must also comply with the principles outlined in the declaration and the ICH guidelines. The protocol, proposed ICF, and other patient information must be reviewed by IRB/IEC before the conduct of this study. A signed and dated statement indicating that the protocol and the ICF have been approved must be provided to the Institution before the start of the study.

The Institute of Hematology and Blood Diseases Hospital, Chinese Academy of Medical Sciences (Institute of Hematology, Chinese Academy of Medical Sciences) is the sponsor of the study. The Principal Investigator and the on-site investigator will prepare study-related documents (if applicable) and to submit to appropriate institutions and obtain written approval for this study. Approval will be obtained before the start of the study.

Participating sites will be provided with copies of the IRB/IEC's approved protocol and ICF. The approval of the protocol and ICF must specify the date of approval, protocol number, and version or revision number.

The participating sites are responsible for notifying the Principal Investigator, Safety Monitoring office, and IRB/IEC of any major deviations from the protocol, or any other condition that might pose additional risks to the patients. Any advertisements used to recruit patients must be reviewed and approved by the Principal Investigator and IRB/IEC before use.

## 13.2 Protocol Amendment

As the study progresses, any revisions to this protocol that appear appropriate will be submitted to the IRB/IEC for written approval before implementation of the revised version. The written approval signed by the IRB/IEC should specifically mention the investigator, protocol number, and title, as well as any applicable revision number. Administrative revisions do not require IRB/IEC approval, but will be submitted to the IRB/IEC for reference.

## 13.3 Informed Consent

In accordance with GCP, investigators must obtain informed consent from patients or their designees before any study-related procedures. The documentation of informed

consent and the informed consent obtaining process should be recorded in the patient's source documents before the patient enters the study. Patients will be informed that their participation is voluntary, and they can withdraw their consent at any time. Patients will be informed that if they don't participate in the study, it will not affect the treatment received, they can use alternative treatments, and such refusal will not affect future treatments. Patients or legally representatives will have sufficient time to read the ICF and will have the opportunity to ask questions. After the interpretation and before the entry to the study, the ICF should be signed by the patients or legally representatives. After consent is obtained, a copy of the ICF must be provided to the patient.

If the patient or a legally representative is unable to read or write, an impartial witness should be present throughout the informed consent process (including reading and interpreting all written information) and sign the ICF in person after the consent is obtained.

The original consent form, signed and dated by the patient and investigator, must be kept in the Investigator's Study File before the patient enters the study, and a copy should be provided to the patient. In addition, if the protocol is amended and affects the content of the ICF, the ICF must be updated. Patients participating in the study must re-consent to the revised ICF when a protocol amendment is implemented. The amended consent form, signed and dated by the patient and the investigator, must be kept in the Investigator's Study File, and a copy should be provided to the patient.

# 14. Data Handling and Documentation

## 14.1 Data/Document

The investigator must ensure that records and documents related to the conduct of the study and dispensing of the study drug, ie, copies of CRFs and source files, source documents, data and records (eg, medical records; clinical and office charts; laboratory notes; memorandum; listing of patient diaries or assessments; dispensing records of pharmacy; recorded data from automated instruments; a certified true copy or transcript; microfilm; photographic negatives, microfilm or magnetic media; X-ray; patient files) and records kept in the pharmacy, laboratory and medical technical departments involved in clinical studies are complete, accurate, and documented.

## 14.2 Data Management

Data will be entered into the clinical database. These data will be validated electronically using online checks during data entry, as well as through programmed editing checks as specified by the study team. If necessary, discrepancies in the data will be brought to the attention of the study team and site personnel in the form of a Data Clarification Form. Solutions to these queries will be reflected in the database. An audit trail within the system will track all changes made to data.

## 14.3 Record Retention

The investigator must maintain records of all study files and supporting information relevant to the conduct of the study. Documents include, but are not limited to, the protocol, CRFs, patient participation reports, AE reports, patient source data, correspondence with health authorities and IRB/IEC, ICFs, investigator curricula vitae, monitoring visit logs, laboratory reference ranges, laboratory accreditation or quality control procedures, and laboratory director curricula vitae. Patient files and other source data must be kept for the maximum period allowed by the hospital, institution, or private clinic. If the investigator wishes to assign study files to others, move them to another location, or is unable to retain them for a specified period, he/she must consult the CRA. The investigator must retain study records in accordance with local laws or requirements, whichever is longer. CRA will notify the investigator of the retention date. All study files should be available upon request. Essential documents should be retained until at least 2 years after the last approval of a marketing application in an ICH region and until there are no pending or contemplated marketing applications in an ICH region or at least 2 years have elapsed since the formal discontinuation of clinical development of the study treatment. These documents should be retained for a longer period. If required by other applicable regulatory requirements, these documents should be retained for a longer period of time.

# 15. Confidentiality

The collection and processing of personal data from patients enrolled in this study will be limited to those data that are necessary to investigate the efficacy, safety, quality, and effectiveness of the investigational product used in this study. Adequate precautions must be taken for the collection and processing of such data to ensure confidentiality and compliance with applicable laws and regulations on data privacy protection.

The Investigator (Sponsor) ensures that personal data will be:

1. processed fairly and legally
2. collected for specific, explicit, and legitimate purposes, and will not be further processed for purposes other than study
3. adequate, relevant and not excessive for the purposes set out above
4. accurate and keep up-to-date as necessary

Before data collection, consent for processing of personal data will be obtained from the patient (or the legally representative). Such consent should also include the transfer of data to other entities or countries. Patients have the right to request access to their personal data through an investigator and to request correction of any incorrect or incomplete data. Applicable steps should be taken to respond to such requests, taking into account the nature of the request, the conditions of the study, and applicable laws and regulations.

Enrolled patients will be registered on website before treatment is initiated. Patient names will not be recorded in the data center. A serial identification number will be automatically assigned to each patient enrolled in the trial. This number will be used for identification and must be included in all CRFs.

## 16. References


1. Swerdlow SH, Campo E, Pileri SA, Harris NL, Stein H, Siebert R, et al. The 2016 revision of the World Health Organization classification of lymphoid neoplasms. **Blood 2016**, 127(20): 2375-2390.
2. Loughran TJ, Kadin ME, Starkebaum G, Abkowitz JL, Clark EA, Disteche C, et al. Leukemia of large granular lymphocytes: association with clonal chromosomal abnormalities and autoimmune neutropenia, thrombocytopenia, and hemolytic anemia. **Ann Intern Med 1985**, 102(2): 169-175.
3. Dinmohamed AG, Brink M, Visser O, Jongen-Lavrencic M. Population-based analyses among 184 patients diagnosed with large granular lymphocyte leukemia in the Netherlands between 2001 and 2013. **Leukemia 2016**, 30(6): 1449-1451.
4. Boeckx N, Uyttebroeck A, Langerak AW, Brusselmans C, Goossens W, Bossuyt X. Clonal proliferation of T-Cell large granular lymphocytes. **Pediatr Blood Cancer 2004**, 42(3): 275-277.
5. Clemente MJ, Wlodarski MW, Makishima H, Viny AD, Bretschneider I, Shaik M, et al. Clonal drift demonstrates unexpected dynamics of the T-cell repertoire in T-large granular lymphocyte leukemia. **Blood 2011**, 118(16): 4384-4393.
6. Lamy T, Moignet A, Loughran TJ. LGL leukemia: from pathogenesis to treatment. **Blood 2017**, 129(9): 1082-1094.
7. Koskela HL, Eldfors S, Ellonen P, van Adrichem AJ, Kuusanmaki H, Andersson EI, et al. Somatic STAT3 mutations in large granular lymphocytic leukemia. **N Engl J Med 2012**, 366(20): 1905-1913.
8. Jerez A, Clemente MJ, Makishima H, Koskela H, Leblanc F, Peng NK, et al. STAT3 mutations unify the pathogenesis of chronic lymphoproliferative disorders of NK cells and T-cell large granular lymphocyte leukemia. **Blood 2012**, 120(15): 3048-3057.
9. Sanikommu SR, Clemente MJ, Chomczynski P, Afable MN, Jerez A, Thota S, et al. Clinical features and treatment outcomes in large granular lymphocytic leukemia (LGLL). **Leuk Lymphoma 2018**, 59(2): 416-422.
10. Barila G, Teramo A, Calabretto G, Vicenzetto C, Gasparini VR, Pavan L, et al. Stat3 mutations impact on overall survival in large granular lymphocyte leukemia: a single-center experience of 205 patients. **Leukemia 2020**, 34(4): 1116-1124.
11. Lamy T, Moignet A, Loughran TJ. LGL leukemia: from pathogenesis to treatment. **Blood 2017**, 129(9): 1082-1094.
12. Yoon HJ, Sugamura K, Loughran TJ. Activation of leukemic large granular lymphocytes by interleukin-2 via the p75 interleukin-2 receptor. **Leukemia 1990**, 4(12): 848-850.
13. Teramo A, Gattazzo C, Passeri F, Lico A, Tasca G, Cabrelle A, et al. Intrinsic and extrinsic mechanisms contribute to maintain the JAK/STAT pathway aberrantly activated in T-type large granular lymphocyte leukemia. **Blood 2013**, 121(19): 3843-3854, S1.
14. Zambello R, Facco M, Trentin L, Sancetta R, Tassinari C, Perin A, et al. Interleukin-15 triggers the proliferation and cytotoxicity of granular lymphocytes

in patients with lymphoproliferative disease of granular lymphocytes. **Blood 1997**, 89(1): 201-211.

15. Mishra A, Liu S, Sams GH, Curphey DP, Santhanam R, Rush LJ, et al. Aberrant overexpression of IL-15 initiates large granular lymphocyte leukemia through chromosomal instability and DNA hypermethylation. **Cancer Cell 2012**, 22(5): 645-655.
16. Kothapalli R, Nyland SB, Kusmartseva I, Bailey RD, Mckeown TM, Loughran TJ. Constitutive production of proinflammatory cytokines RANTES, MIP-1beta and IL-18 characterizes LGL leukemia. **Int J Oncol 2005**, 26(2): 529-535.
17. Yang J, Liu X, Nyland SB, Zhang R, Ryland LK, Broeg K, et al. Platelet-derived growth factor mediates survival of leukemic large granular lymphocytes via an autocrine regulatory pathway. **Blood 2010**, 115(1): 51-60.
18. Sun H, Wei S, Yang L. Dysfunction of immune system in the development of large granular lymphocyte leukemia. **Hematology 2019**, 24(1): 139-147.
19. Moignet A, Lamy T. Latest Advances in the Diagnosis and Treatment of Large Granular Lymphocytic Leukemia. **Am Soc Clin Oncol Educ Book 2018**, 38: 616-625.
20. Dhodapkar MV, Li CY, Lust JA, Tefferi A, Phyliky RL. Clinical spectrum of clonal proliferations of T-large granular lymphocytes: a T-cell clonopathy of undetermined significance? **Blood 1994**, 84(5): 1620-1627.
21. Cheon H, Dziewulska KH, Moosic KB, Olson KC, Gru AA, Feith DJ, et al. Advances in the Diagnosis and Treatment of Large Granular Lymphocytic Leukemia. **Curr Hematol Malig Rep 2020**, 15(2): 103-112.
22. Lamy T, Moignet A, Loughran TJ. LGL leukemia: from pathogenesis to treatment. **Blood 2017**, 129(9): 1082-1094.
23. Sanikommu SR, Clemente MJ, Chomczynski P, Afable MN, Jerez A, Thota S, et al. Clinical features and treatment outcomes in large granular lymphocytic leukemia (LGLL). **Leuk Lymphoma 2018**, 59(2): 416-422.
24. Bareau B, Rey J, Hamidou M, Donadieu J, Morcet J, Reman O, et al. Analysis of a French cohort of patients with large granular lymphocyte leukemia: a report on 229 cases. **Haematologica 2010**, 95(9): 1534-1541.
25. Loughran TJ, Zickl L, Olson TL, Wang V, Zhang D, Rajala HL, et al. Immunosuppressive therapy of LGL leukemia: prospective multicenter phase II study by the Eastern Cooperative Oncology Group (E5998). **Leukemia 2015**, 29(4): 886-894.
26. Qiu ZY, Fan L, Wang R, Gale RP, Liang HJ, Wang M, et al. Methotrexate therapy of T-cell large granular lymphocytic leukemia impact of STAT3 mutation. **Oncotarget 2016**, 7(38): 61419-61425.
27. Moignet A, Hasanali Z, Zambello R, Pavan L, Bareau B, Tournilhac O, et al. Cyclophosphamide as a first-line therapy in LGL leukemia. **Leukemia 2014**, 28(5): 1134-1136.
28. Poullot E, Zambello R, Leblanc F, Bareau B, De March E, Roussel M, et al. Chronic natural killer lymphoproliferative disorders: characteristics of an international cohort of 70 patients. **Ann Oncol 2014**, 25(10): 2030-2035.

29. Dhodapkar MV, Lust JA, Phyliky RL. T-cell large granular lymphocytic leukemia and pure red cell aplasia in a patient with type I autoimmune polyendocrinopathy: response to immunosuppressive therapy. **Mayo Clin Proc 1994**, 69(11): 1085-1088.
30. Zhu Y, Gao Q, Hu J, Liu X, Guan D, Zhang F. Clinical features and treatment outcomes in patients with T-cell large granular lymphocytic leukemia: A single-institution experience. **Leuk Res 2020**, 90: 106299.
31. Osuji N, Matutes E, Tjonnfjord G, Grech H, Del GI, Wotherspoon A, et al. T-cell large granular lymphocyte leukemia: A report on the treatment of 29 patients and a review of the literature. **Cancer-Am Cancer Soc 2006**, 107(3): 570-578.
32. Lamy T, Loughran TJ. How I treat LGL leukemia. **Blood 2011**, 117(10): 2764-2774.
33. Matutes E. Large granular lymphocytic leukemia. Current diagnostic and therapeutic approaches and novel treatment options. **Expert Rev Hematol 2017**, 10(3): 251-258.
34. D'Amato RJ, Loughnan MS, Flynn E, Folkman J. Thalidomide is an inhibitor of angiogenesis. **Proc Natl Acad Sci U S A 1994**, 91(9): 4082-4085.
35. Moreira AL, Sampaio EP, Zmuidzinas A, Frindt P, Smith KA, Kaplan G. Thalidomide exerts its inhibitory action on tumor necrosis factor alpha by enhancing mRNA degradation. **J Exp Med 1993**, 177(6): 1675-1680.
36. Sampaio EP, Sarno EN, Galilly R, Cohn ZA, Kaplan G. Thalidomide selectively inhibits tumor necrosis factor alpha production by stimulated human monocytes. **J Exp Med 1991**, 173(3): 699-703.
37. Singhal S, Mehta J, Desikan R, Ayers D, Roberson P, Eddlemon P, et al. Antitumor activity of thalidomide in refractory multiple myeloma. **N Engl J Med 1999**, 341(21): 1565-1571.
38. Richardson PG, Schlossman RL, Weller E, Hideshima T, Mitsiades C, Davies F, et al. Immunomodulatory drug CC-5013 overcomes drug resistance and is well tolerated in patients with relapsed multiple myeloma. **Blood 2002**, 100(9): 3063-3067.
39. Dimopoulos MA, Zomas A, Viniou NA, Grigoraki V, Galani E, Matsouka C, et al. Treatment of Waldenstrom's macroglobulinemia with thalidomide. **J Clin Oncol 2001**, 19(16): 3596-3601.
40. Seldin DC, Choufani EB, Dember LM, Wiesman JF, Berk JL, Falk RH, et al. Tolerability and efficacy of thalidomide for the treatment of patients with light chain-associated (AL) amyloidosis. **Clin Lymphoma 2003**, 3(4): 241-246.
41. Palladini G, Perfetti V, Perlini S, Obici L, Lavatelli F, Caccialanza R, et al. The combination of thalidomide and intermediate-dose dexamethasone is an effective but toxic treatment for patients with primary amyloidosis (AL). **Blood 2005**, 105(7): 2949-2951.
42. Barosi G, Grossi A, Comotti B, Musto P, Gamba G, Marchetti M. Safety and efficacy of thalidomide in patients with myelofibrosis with myeloid metaplasia. **Br J Haematol 2001**, 114(1): 78-83.
43. Canepa L, Ballerini F, Varaldo R, Quintino S, Reni L, Clavio M, et al. Thalidomide

in agnogenic and secondary myelofibrosis. **Br J Haematol 2001**, 115(2): 313-315.
44. Elliott MA, Mesa RA, Li CY, Hook CC, Ansell SM, Levitt RM, et al. Thalidomide treatment in myelofibrosis with myeloid metaplasia. **Br J Haematol 2002**, 117(2): 288-296.
45. Piccaluga PP, Visani G, Pileri SA, Ascani S, Grafone T, Isidori A, et al. Clinical efficacy and antiangiogenic activity of thalidomide in myelofibrosis with myeloid metaplasia. A pilot study. **Leukemia 2002**, 16(9): 1609-1614.
46. Mesa RA, Steensma DP, Pardanani A, Li CY, Elliott M, Kaufmann SH, et al. A phase 2 trial of combination low-dose thalidomide and prednisone for the treatment of myelofibrosis with myeloid metaplasia. **Blood 2003**, 101(7): 2534-2541.
47. Mesa RA, Elliott MA, Faoro L, Tefferi A. Durable and unmaintained remissions with thalidomide-based drug therapy in myelofibrosis with myeloid metaplasia: Long term outcome analysis of 2 prospective trials.; 2003 2003-01-01; 2003. p. 33A.
48. Raza A, Meyer P, Dutt D, Zorat F, Lisak L, Nascimben F, et al. Thalidomide produces transfusion independence in long-standing refractory anemias of patients with myelodysplastic syndromes. **Blood 2001**, 98(4): 958-965.
49. Zorat F, Shetty V, Dutt D, Lisak L, Nascimben F, Allampallam K, et al. The clinical and biological effects of thalidomide in patients with myelodysplastic syndromes. **Br J Haematol 2001**, 115(4): 881-894.
50. Strupp C, Germing U, Aivado M, Misgeld E, Haas R, Gattermann N. Thalidomide for the treatment of patients with myelodysplastic syndromes. **Leukemia 2002**, 16(1): 1-6.
51. Steins MB, Padro T, Bieker R, Ruiz S, Kropff M, Kienast J, et al. Efficacy and safety of thalidomide in patients with acute myeloid leukemia. **Blood 2002**, 99(3): 834-839.
52. Thomas DA, Estey E, Giles FJ, Faderl S, Cortes J, Keating M, et al. Single agent thalidomide in patients with relapsed or refractory acute myeloid leukaemia. **Br J Haematol 2003**, 123(3): 436-441.
53. Teramo A, Gattazzo C, Passeri F, Lico A, Tasca G, Cabrelle A, et al. Intrinsic and extrinsic mechanisms contribute to maintain the JAK/STAT pathway aberrantly activated in T-type large granular lymphocyte leukemia. **Blood 2013**, 121(19): 3843-3854, S1.
54. Thomas S, Fisher KH, Snowden JA, Danson SJ, Brown S, Zeidler MP. Methotrexate Is a JAK/STAT Pathway Inhibitor. **Plos One 2015**, 10(7): e130078.
55. Loughran TJ, Kidd PG, Starkebaum G. Treatment of large granular lymphocyte leukemia with oral low-dose methotrexate. **Blood 1994**, 84(7): 2164-2170.
56. Bareau B, Rey J, Hamidou M, Donadieu J, Morcet J, Reman O, et al. Analysis of a French cohort of patients with large granular lymphocyte leukemia: a report on 229 cases. **Haematologica 2010**, 95(9): 1534-1541.
57. Wechalekar AD, Goodman HJ, Lachmann HJ, Offer M, Hawkins PN, Gillmore JD. Safety and efficacy of risk-adapted cyclophosphamide, thalidomide, and dexamethasone in systemic AL amyloidosis. **Blood 2007**, 109(2): 457-464.
58. Zhang L, Zhao AL, Duan MH, Li ZY, Cao XX, Feng J, et al. Phase 2 study using

oral thalidomide-cyclophosphamide-prednisone for idiopathic multicentric Castleman disease. **Blood 2019**, 133(16): 1720-1728.

# 17. Appendices

## Appendix 1. ECOG Performance Status

| Grade | ECOG |
|---|---|
| 0 | Fully active, able to carry on all pre-disease performance without restriction |
| 1 | Restricted in physically strenuous activity but ambulatory and able to carry out work of a light or sedentary nature, e.g., light housework, office work |
| 2 | Ambulatory and capable of all self-care but unable to carry out any work activities. Up and about more than 50% of waking hours |
| 3 | Capable of only limited self-care, confined to bed or chair more than 50% of waking hours |
| 4 | Completely disabled. Cannot carry on any selfcare. Totally confined to bed or chair |
| 5 | Dead |

Abbreviation: ECOG, Eastern Cooperative Oncology Group.

## Appendix 2. Calculation of Creatinine Clearance

Cockcroft-Gault formula:

The patient's creatinine clearance (CrCl) is calculated using the following Cockcroft-Gault formula:

CrCl = 1.222 × [(140-age) × body weight (kg)]/serum creatinine (μmol/L)

Multiply by 0.85 in case of a female patient

If serum creatinine is expressed in the International System of Units (SI) (ie, μmol/L), convert SI units to conventional units (mg/dL) using the following formula (Manual of Laboratory Testing and Diagnosis, 2004):

- Serum creatinine (μmol/L) ÷ 88.4 = serum creatinine (mg/dL)

## Appendix 3. Thalidomide Neurotoxicity Grading and Dose Adjustment

| | 0 | 1 | 2 | 3 | 4 |
|---|---|---|---|---|---|
| | Normal | Asymptomatic: Loss of deep tendon reflexes or paresthesia (including tingling), but not interfering with function | Sensory alteration or paresthesia (including tingling) interfering with function but not with activities of daily living | Sensory alteration or paresthesia interfering with activities of daily living | Disabling |
| Not applicable | No action | Thalidomide reduced by one dose level | Thalidomide reduced by two dose levels | Thalidomide withheld | Thalidomide withheld |
| Slight pain but not interfering with function | No action | Thalidomide reduced by two dose levels | Thalidomide discontinued | Thalidomide withheld | Thalidomide withheld |
| Moderate pain (interfering with function but not with activities of daily living) | Thalidomide reduced by one dose level | Thalidomide reduced by two dose levels | Thalidomide withheld | Thalidomide withheld | Thalidomide withheld |
| Severe pain (interfering with activities of daily living) | Thalidomide reduced by one dose level | Thalidomide reduced by two dose levels | Thalidomide withheld | Thalidomide withheld | Thalidomide withheld |
| Disabling | Thalidomide withheld | Thalidomide withheld | Thalidomide withheld | Thalidomide withheld | Thalidomide withheld |

Notes:

Withheld: Thalidomide should be withheld for 2 weeks until the toxicity returns to Grade 1 or better condition.

Reduced by one dose level: Thalidomide reduced from 100 mg/d to 50 mg/d.

Reduced by two dose levels: Thalidomide reduced from 100 mg/d to 50 mg, every other day.

# Phase II Trial of Thalidomide for symptomatic large granular lymphocyte leukemia

## CLINICAL STUDY PROTOCOL

**Protocol Number: BDH/LGLL01**
**Version: 2.0**
**Version Date: 30 January 2021**

**Clinical trial sponsor: State Key Laboratory of Experimental Hematology, National Clinical Research Center for Blood Diseases, Haihe Laboratory of Cell Ecosystem, Institute of Hematology & Blood Diseases Hospital, Chinese Academy of Medical Sciences & Peking Union Medical College, Tianjin 300020, China; Tianjin Institutes of Health Science**

This document contains confidential information, which should not be copied, released or published without Institute of Hematology & Blood Diseases Hospital

# PROTOCOL HISTORY

| Version | Version date |
|---|---|
| 1.0 | 25 April 2020 |
| 2.0 | 30 January 2021 |

# PROTOCOL AMENDMENT

| Summary of changes for version 2.0 | Rationale | Protocol section(s) |
|---|---|---|
| Changed prednisone taper time from 12 months to 3 months | It has been reported that the use of steroids in combination with immunosuppressive therapy such as MTX in LGLL patients could accelerate resolution of B symptoms but not to retain the response or increase ORR. A long-term therapy with steroid may increase the rate of side effects. So, we changed the taper time from 12 months to 3 months | Synopsis, Section 2.6.1, Section 4.1, Section 6.1 |
| Patients with high-risk of Caprini score added to exclusion criteria | It has been proved that thalidomide with steroids could increases the risk of thromboembolism, especially venous thromboembolism. To reduce the risk of occurrence of thromboembolism, patients with high-risk of Caprini score will be exclude form this study. | Synopsis, Section 5.2.2, Appendix 2 |
| Removed TTNT as secondary objective | Patients are withdrawn from the study when their experienced disease progresses, and some patients are lost to follow-up after they withdraw from this study, making it difficult to obtain time to start the next line of treatment. | Synopsis, Section 9.2, Section 9.3, Section 11.1, Section 11.3 |
| Identified that specific indicators of disease progression | The definition of disease progression is vague, and different doctors may judge the disease progression differently. To reduce these biases, we establish the unified criteria | Section 9.2 |

# Table of Contents


List of Abbreviations ........ 7

1. Synopsis ........ 9

2. Background ........ 16

2.1 Large Granular Lymphocytic Leukemia ........ 16

2.2 Current Treatment Landscape ........ 17

2.3 Investigational Product -Thalidomide ........ 19

2.4 Investigational Product - Methotrexate ........ 23

2.5 Rationale for Thalidomide Combined with Methotrexate and Prednisone in the Treatment of LGLL ........ 26

2.6 Risk/Benefit Assessment ........ 27

2.6.1 Investigational Plan ........ 27

2.6.2 Potential Risks ........ 27

2.6.3 Potential Benefits ........ 28

2.6.4 Assessment of Potential Risks and Benefits ........ 28

3. Objectives ........ 29

4. Study Design ........ 30

4.1 Overall Study Design ........ 30

4.2 Description of Overall Study Design and Plan ........ 30

4.3 Duration of Treatment ........ 31

4.4 Follow-up ........ 31

4.5 Sample Size ........ 31

4.6 Steering Committee ........ 31

4.7 Independent Data Monitoring Committee ........ 32

4.8 Study Termination ........ 32

5 Study Population ........ 33

5.1 Eligibility Criteria for Prescreening ........ 33

5.2 Inclusion and Exclusion Criteria ........ 33

5.2.1 Inclusion Criteria ........ 33

5.2.2 Exclusion Criteria ........ 34

5.3 Withdrawal Criteria ........ 34

5.4 Removal of Patients from Therapy or Assessment ........ 35

5.5 Identification Codes, Enrollment, and Screening Log of Screening Failed Patients 35

6 Treatments ....................................................................................37

6.1 Treatment Regimen....................................................................37

6.2 Administration of Study Drugs ..................................................37

6.3 Concomitant Medications and Supportive Care ........................37

6.3.1 Prophylactic Medications.........................................................38

6.3.2 Permitted Medications .............................................................38

6.3.3 Prohibited Medications ............................................................39

6.4 Source and Use of Study Drugs ..................................................40

7 Adverse Reactions and Dose Adjustment .....................................41

7.1 Common Adverse Reactions of Thalidomide.............................41

7.2 Common Adverse Reactions of Methotrexate............................42

7.3 Common Adverse Reactions of Prednisone ...............................43

7.4 Guidelines for Dose Adjustment and Discontinuation ..............44

7.4.1 Dose Adjustment and Discontinuation of Thalidomide.............45

7.4.2 Dose Adjustment and Discontinuation of Methotrexate............45

7.4.3 Dose Adjustment and Discontinuation of Prednisone ..............45

7.5 Other Treatment Considerations................................................45

8. Study Procedures ........................................................................46

8.1 Screening Period .........................................................................46

8.2 Treatment Period .........................................................................47

8.2.1 Disease Progression ..................................................................49

8.3 List of Tests and Key Time Points...............................................49

8.4 Early Withdrawal (Discontinuation of Study Treatment)...........50

8.5 Follow-up.....................................................................................50

8.5.1 Survival Follow-up ....................................................................50

8.5.2 Safety Follow-up........................................................................51

9. Efficacy Evaluation .....................................................................52

9.1 Definition .....................................................................................52

9.1.1 General Definition .....................................................................52

9.2 Efficacy Evaluation......................................................................52

9.3 Study Endpoints Definition and Evaluation ....52
10. Safety Evaluation ....54
10.1 Definition and Classification of Adverse Events ....54
10.2 Safety Evaluation ....56
10.3 Recording of Adverse Events ....57
10.4 Reporting of Adverse Events ....58
10.5 Data Safety ....58
11. Statistical Analysis ....60
11.1 General Consideration ....60
11.2 Safety Analysis ....60
11.3 Efficacy Analysis ....60
11.4 Biomarkers Exploratory Analysis ....61
12. Quality Control and Assurance of Good Clinical Practice ....62
12.1 Monitoring, Audit, and Inspection ....62
12.2 Investigator’s Responsibilities ....62
13. Ethical and Regulatory Considerations ....64
13.1 Review and Approval by Institutional Review Board/Independent Ethics Committee ....64
13.2 Protocol Amendment ....64
13.3 Informed Consent ....64
14. Data Handling and Documentation ....66
14.1 Data/Document ....66
14.2 Data Management ....66
14.3 Record Retention ....66
15. Confidentiality ....67
16. References ....68
17. Appendices ....73

# List of Abbreviations

| Abbreviation | Definition |
|---|---|
| AE | adverse event |
| ALT | alanine aminotransferase |
| AML | acute myeloid leukemia |
| ANC | absolute neutrophil count |
| AST | aspartate aminotransferase |
| CLPD-NK | chronic lymphoproliferative disorders of nature killer cells |
| $C_{max}$ | peak concentration |
| CrCl | creatinine clearance |
| $C_{ssmax}$ | steady-state peak concentration |
| CT | computed tomography |
| CTCAE | Common Terminology Criteria for Adverse Events |
| CTD | cyclophosphamide, thalidomide, and dexamethasone |
| CR | complete remission |
| CRA | clinical research associate |
| CRF | case report form |
| CRR | complete remission rate |
| DoR | duration of response |
| DRO | Data Request Order |
| DSMC | Data Safety Monitoring Committee |
| ECG | electrocardiograph |
| ECOG | Eastern Cooperative Oncology Group |
| EOS | end of study |
| GCP | good clinical practice |
| HBV | hepatitis B virus |
| HCV | hepatitis C virus |
| ICH | International Council for Harmonisation of Technical Requirements for Pharmaceuticals for Human Use |
| IDMC | Independent Data Monitoring Committee |
| ICF | informed consent form |
| IEC | Independent Ethics Committee |
| IL | interleukin |
| IRB | Institutional Review Board |
| LDH | lactate dehydrogenase |
| LGL | large granular lymphocyte |
| LGLL | Large granular lymphocyte leukemia |
| mDoR | median duration of response |
| MDS | myelodysplastic syndrome |
| MM | multiple myeloma |
| MMM | myelofibrosis with myeloid metaplasia |
| NCI | National Cancer Institute |
| NK | nature killer |
| ORR | overall response rate |
| OS | overall survival |
| PD | disease progression |
| PFS | progression-free survival |

| | |
|---|---|
| PR | partial remission |
| QD | once daily |
| QOD | every other day |
| SAE | serious adverse event |
| SD | stable disease |
| STAT3 | signal transducer and activator of transcription 3 |
| TLS | tumor lysis syndrome |
| TNF-α | tumor necrosis factor alpha |
| TPM | thalidomide combined with methotrexate and prednisone |
| T-LGLL | T-cell large granular lymphocytic leukemia |
| ULN | upper limit of normal |
| US | United States |
| WBC | white blood cell |
| WHO | World Health Organization |
| WM | Waldenström macroglobulinemia |

# 1. Synopsis

**Project Version and Date.:** Version 2.0/30 January 2021

**Study Drugs:** Thalidomide, methotrexate, and prednisone

**Study Phase: Phase II**

**Study Title:** Thalidomide for symptomatic large granular lymphocyte leukemia

**Study Population:** Treatment-naive patients, or patients treated with or relapsed from a non-methotrexate /thalidomide-based regimen

**Principal Investigators:** Lugui Qiu and Shuhua Yi

**Primary Objective:**

- To evaluate the complete remission rate (CRR) of symptomatic large granular lymphocyte leukemia (LGLL) patients treated with thalidomide combined with methotrexate and prednisone (TPM) regimen

**Secondary Objectives:**

- To evaluate the safety and tolerance of symptomatic LGLL patients treated with TPM regimen
- To evaluate the overall response rate (ORR) and partial remission (PR) rate of symptomatic LGLL patients treated with TPM regimen
- To evaluate the progression-free survival (PFS) and overall survival (OS) of symptomatic LGLL patients treated with TPM regimen
- To evaluate the duration of response (DoR) in symptomatic LGLL patients treated with TPM regimen

**Exploratory Objective:**

- To explore biomarkers significantly and potentially associated with clinical efficacy

**Study Design:**

This is a prospective multicenter Phase II clinical study employing an oral regimen administered in 3-month cycles. Patients will receive thalidomide of 50 to 100 mg once daily (QD) before bedtime, prednisone of 0.5 to 1.0 mg/kg every other day (QOD), methotrexate of 10 mg/m$^2$/week for up to 4 cycles. Pred was gradually tapered after 3 months. Patients who achieved a CR could discontinue MTX after one cycle consolidation, with a maximum duration of four cycles. For patients who achieved a partial remission (PR) or CR, Thal maintenance therapy was recommended to be continued for a maximum duration of two years, but also depended on patients or investigator's choices.

Efficacy evaluation will be conducted at the end of each cycle starting from Cycle 1. The efficacy evaluations will continue until disease progression (PD), discontinuation

of treatment (for patients who continue treatment after PD), initiation of a new anti-cancer therapy, or withdrawal from the study (eg, death, patient's request, loss of follow-up, etc.), whichever occurs first.

The primary objective of this study is to evaluate the efficacy and safety of TPM regimen and further explore the biomarkers associated with clinical efficacy.

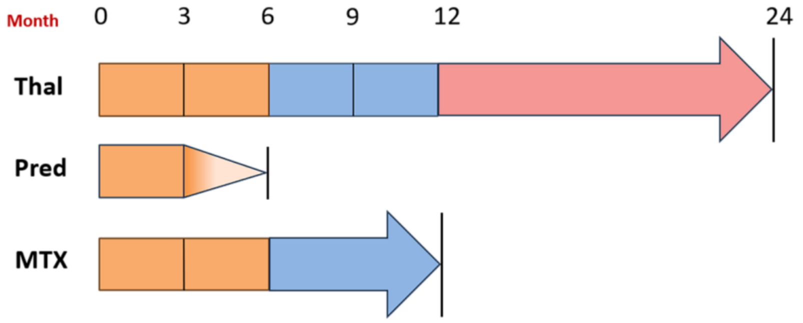


Abbreviations: CR, complete remission; MTX, methotrexate; Pred, Prednisone; Thal, thalidomide.

Notes:

Thal: 50 to 100 mg, oral, before bed, for 2 years

Pred: 0.5to 1 mg/kg, oral, every other day, could be tapered after 3 months

MTX: 10 mg/m$^2$, once weekly, patients who achieve CR can discontinue MTX after 4 months of consolidation therapy, with a maximum duration of 1 year

**Description of Overall Study Design and Plan:**

The study consists of a 28-day screening period, a treatment period (up to half a year for prednisone, up to 1 year for methotrexate, and 2 years for thalidomide), and a follow-up period (safety and efficacy follow-up). Patients who provide the signed informed consent will undergo baseline examinations during the screening period. Prior treatment and efficacy will also be evaluated before enrollment. Patients who met the inclusion/exclusion criteria will enter the treatment period. Relevant examinations specified in the protocol will be completed for all patients during treatment to observe efficacy, safety, and hemogram changes after discontinuation of the study drugs. The follow-up period will begin after the end of the treatment period.

**Duration of Treatment:**

Patients enrolled in this study will be treated with thalidomide combined with methotrexate and prednisone for 3 months and then evaluated for efficacy. Based on the efficacy evaluation, patients will either continue treatment as per the initial regimen or be withdrawn from the study. The Institute of Hematology and Blood Diseases Hospital, Chinese Academy of Medical Sciences, is a specialized hospital for hematology, where treats 30 to 40 LGLL patients every year, of which approximately 5 to 10 are eligible for enrollment. A total of 5 to 10 patients are expected to be enrolled in multiple centers nationwide every year. The enrollment period of this study is

expected to span 2 years. This study is of a limited course of treatment. Patients who obtain sustained clinical benefits from the study treatment without consent withdrawal, PD, and intolerable toxicity may continue the study and receive follow-up. The end of study (EOS; study completion) is defined as the time when the last patient completes the last visit.

**Sample Size:**

A retrospective study of 45 patients is used as the historical control, in which cyclophosphamide monotherapy was applied with an effective rate of 72% and CRR of 47%. From the perspective of clinical expertise, taking the CRR of 70% to 80% as the minimum threshold for clinical significance, the sample size of 35 patients in this group is deemed sufficient to test the overall true CRR that is superior to that of the historical control at a false positive rate $\alpha = 0.05$ (two sides) and false negative rate $\beta = 0.2$ (ie, power = 80%). With a 20% dropout rate, at least 42 patients would be required to enroll in this study.

**Inclusion and Exclusion Criteria:**

**Inclusion Criteria:**

1. Patients who fulfill the diagnostic criteria for T-LGLL or chronic lymphoproliferative disorders of nature killer cells (CLPD-NK) specified in the 2016 World Health Organization (WHO) Classification of Lymphoid Neoplasms [1]
2. Patients are fully informed about the study and willing to participate in the study and provide written informed consent form (ICF)
3. Male or female, aged ≥18 years
4. Treatment-naive patients, or patients treated with or relapsed from a non-methotrexate/ thalidomide-based regimen
5. Patients with at least one of the following indications for LGLL treatment:
    a. absolute neutrophil count (ANC) <0.5 × $10^9$/L, or neutropenia with recurrent infections
    b. Hemoglobin <100 g/L, or requiring infusion of red blood cells
    c. Platelets level <50 × $10^9$/L
    d. Concomitant autoimmune diseases requiring treatment
    e. Symptomatic splenomegaly
    f. Severe B symptoms (fever of unknown cause with body temperature over 38 °C; night sweats; weight loss of ≥10% within half a year)
    g. Pulmonary hypertension
6. Patients with Eastern Cooperative Oncology Group (ECOG) score of 0 to 2 (Appendix 1)
7. Patients with life expectancy ≥6 months.

**Exclusion Criteria:**

1. Patients not diagnosed with T-LGLL or CLPD-NK

2. Patients with no indication for LGLL treatment

3. Patients who are unable to understand or follow study procedures

4. Patients who have been diagnosed or treated for malignancies other than LGLL within the past five years

5. Patients with non-lymphoma-related hepatic and renal impairment: alanine aminotransferase (ALT) > 3 × upper limit of normal (ULN), aspartate aminotransferase (AST) > 3 × ULN, total bilirubin > 2 × ULN, and serum creatinine clearance (CrCl) <30 mL/min

6. Patients with other severe diseases that have an impact on this study (uncontrolled diabetes, stomach ulcers, other severe cardiopulmonary conditions, etc.), at the discretion of investigators.

7. High-risk patients based on the Caprini score for thromboembolism (Appendix 2)

8. Patients with a known history of HIV infection, or with active hepatitis B virus (HBV) infection, or any uncontrolled active systemic infection requiring intravenous antibiotics

Notes: HBV infection is considered active if all following criteria are met: a. HBV DNA quantification ≥2000 IU/mL; b. ALT ≥2 times × ULN; c. LGLL, medications, and other causes-induced hepatitis are excluded. Patients with active HBV infection at the time of initial diagnosis who are converted to inactive HBV infection after adequate anti-HBV treatment could be enrolled in this study.

9. Patients who have undergone major surgery (excluding lymph node biopsy) within the past 14 days or who are expected to undergo major surgery during study treatment

10. Pregnant or lactating women, and women of childbearing potential who have not taken contraceptive measures

11. Patients who are allergic to the agents or the ingredients

**Withdrawal Criteria:**

1. Patients withdraw the ICFs

2. Patients experience PD or death

3. Patients experience intolerable toxicity that cannot be relieved after symptomatic treatment

4. Patients are unwilling to continue treatment

5. There was no clinical benefit for more than half a year

6. Delayed dosing for more than 4 weeks and patients are judged by investigators to be unsuitable for further dosing

7. Pregnancy

8. Lost to follow-up

9. Treatment should be discontinued based on the best interests of the patients at the investigator' discretion

10. Patients does not comply with the protocol, and the investigator deems it necessary to discontinue the treatment

11. The collaborator or regulatory authority notifies to end the clinical study.

**Dosage Form, Dosage, and Route of Administration:**

Treatment will be provided in 3-month cycles. Patients will receive thalidomide of 50 to 100 mg orally QD before bedtime, methotrexate of 10 mg/m$^2$ orally once weekly, and prednisone of 0.5 to 1 mg/kg orally QOD. Prednisone will be tapered after 3 months. Methotrexate will be dosed for up to 1 year. Thalidomide will be dosed for up to 2 years or discontinued based on patients or investigator's choices. Efficacy was evaluated after 3 months of treatment, if PR or above response to treatment, the initial treatment regimen will be continued. If there is no clinical benefit after 6 months of initial treatment, patients will switch to the second-line treatment was and will be withdrawn from the study.

**Study Endpoints:**

**Primary Endpoint:**

- CRR of patients with symptomatic LGLL treated with thalidomide, prednisone, and methotrexate.

**Secondary Endpoints:**

**1. Secondary Safety Endpoint**

- Incidence and severity of hematological and non-hematological adverse events (AEs)

Safety endpoints include serious adverse events (SAEs), treatment-emergent adverse events (TEAEs), physical examination (including ECOG performance status), vital sign measurements, standard clinical laboratory parameters, and electrocardiograph (ECG). TEAEs will be graded according to National Cancer Institute (NCI) Common Terminology Criteria for Adverse Events (CTCAE) Version 5.0.

**2. Secondary Efficacy Endpoints**

- Response rates: ORR and PR rate (time frame: 24 months after the last treatment)

Point estimates of rates will be calculated for each protocol analysis set. An estimate of the 95% CI for the response rate will also be derived. Graphical and descriptive analyses will be used to explore associations between relevant markers and responses.

- PFS and OS (time frame: 24 months after the last treatment)

Time from the first day to PD or death from any reason. The median duration of the overall response will be assessed with a 95% CI.

- DoR (time frame: 24 months after the last treatment)

The time when CR or PR is met until the first date of recurrent or progressive disease. The median duration of the overall response will be assessed with a 95% CI.

- Effect of biomarkers on efficacy and adverse reactions

**Exploratory Endpoint:**

- To explore biomarkers significantly and potentially associated with clinical efficacy

**Statistical Analysis:**

The data cut-off date for the primary analysis will be 1 year after the completion of treatment for the last patient. After initial analysis, the primary part of the study will be closed and data will be tracked until completion.

Descriptive statistics will be provided for selected demographic and safety data by group and time as appropriate. For continuous data, descriptive statistics will be presented as mean, median, standard deviation, and range (including geometric mean and geometric coefficient of variation for peak concentration [$C_{max}$]). Categorical data will be summarized as frequency and percentage. Data can also be presented as a graphical abstract.

**Safety Analysis:**

The safety profile will be analyzed based on AEs, physical examination, vital sign measurements, laboratory measurements, and ECG findings. AEs will be graded as per NCI CTCAE (Version 5.0).

In general, the safety analysis will be descriptive and presented in tabular form with appropriate summary statistics. The number of patients and events of hematological and non-hematological toxicity (based on NCI CTCAE Version 5.0) will be listed, and the incidence will be calculated.

**Efficacy Analysis:**

The efficacy analysis will involve CRR and ORR (sum of CR and PR rates). Efficacy variables will be tabulated and summarized. For the numbers and percentages of CRR and ORR, point estimates and 95% exact binomial CIs will be provided.

Time-to-event variables including DoR, PFS, and OS will be summarized descriptively using the Kaplan-Meier method. The rules for DoR, PFS, and OS, analyses will be specified in the Statistical Analysis Plan (SAP). Growth modulation indices (intra-subject ratios of DOR, PFS, and OS, post-study treatment versus DOR, PFS, and OS post the most recent prior treatment regimen) will be summarized.

Data will be summarized using descriptive statistics. Continuous variables will be presented as number, mean, standard deviation, median, and range. Discrete variables will be summarized as frequency.

**Biomarker Exploratory Analysis:**

The exploratory analysis of biomarkers will be tabulated and summarized using descriptive statistics.

# 2. Background

## 2.1 Large Granular Lymphocytic Leukemia

Large granular lymphocyte leukemia (LGLL) is a clonal group of lymphoproliferative diseases that originate from CD3+ cytotoxic T cells or CD3- nature killer (NK) cells. Clinically, LGLL is characterized by infiltration of large granular lymphocytes (LGLs) in peripheral blood and bone marrow, as well as splenomegaly and hemocytopenia. In 2008, the World Health Organization (WHO) classified the disease into 3 subtypes by the cell origin and clinical characteristics, including T-cell large granular lymphocytic leukemia (T-LGLL), chronic lymphoproliferative disorders of nature killer cells (CLPD-NK), and aggressive nature killer cell leukemia (ANKL).[1] T-LGLL and CLPD-NK are similar in clinical manifestations, biological characteristics, treatment options and prognosis, both of which have an inert course of disease. They are LGLL in the true sense, and they are also the research objects of this clinical trial. ANKL belongs to aggressive lymphoma, and its treatment and prognosis are different from the previous two, which is not covered by this clinical trial.

As a rare hematological malignancy, T-LGLL accounts for 2% to 5% of chronic lymphoproliferative diseases in North America and up to 6% in Asia. The incidence of T-LGLL is estimated to be 1/10 million in the United States (US),[2] while a registered Dutch study reported an incidence of 0.72/1 million person-years.[3] The median age of onset of T-LGLL is 60 years (range: 4 to 88 years) without a gender difference. Only 10% of T-LGLL patients are younger than 40 years old. T-LGLL is also rare in children.[4]

The exact mechanism by which LGLs clones proliferated remains unclear. It is generally believed that LGLL is caused by chronic antigenic stimulation caused by viral infection. Initially, an unknown antigen leads to the expansion of oligoclonal LGL, and the continuous stimulation of the antigen leads to the activation of signal transducer and activator of transcription 3 (STAT3) and the emergence of dominant cloning. Studies on T cell lineages have confirmed the transition process from oligoclonal to dominant cloning.[5] Both activation of survival pathways and evasion of apoptosis are primary contributors to the clonal proliferation of LGLs in leukemia, with a complex survival network driven by both internal and external stimuli. Multiple dysregulated pathways coordinate pro-survival and anti-apoptotic signals, including resistance to Fas/FasL-mediated apoptosis, activation of interleukin (IL)-15 and platelet-derived growth factor (PDGF), activation of JAK-STAT, P13K-AKT, RAS-RAF MAPK and NF-kB signaling pathways, and dysregulation of the sphingolipid rheostat.[6] The most common mutations in LGLL are the site-directed mutations of the STAT3 gene.[7-8] It is reported to occur in 28% to 75% of patients with T-LGLL. STAT3 forms a dimer upon phosphorylation, which enters the nucleus and functions as a transcription factor to enhance anti-apoptotic pathways, resulting in cytokine-independent proliferation and survival of leukemic cells. Patients with STAT3 mutations have a higher incidence of neutropenia, anemia, and autoimmune diseases, which generally require treatment.[9-10]

LGLL is at the "intersection" of a clonal lymphoproliferative disease, autoimmunity, and chronic inflammation.[11] Chronic antigen stimulation is a trigger for the LGL activation and proliferation, while a strong pro-inflammatory environment plays a crucial role in the pathogenesis of LGLL. Compared with healthy people, multiple cytokines and chemokines (eg, IL-2,[12] IL-6,[13] IL-15[14-15], IL-18,[16] RANTES,[16] and PDGF [17]) significantly increased in the serum of LGLL patients, thereby promoting survival and proliferation of LGL. It has been reported that patients with LGLL could present with a series of changes in immunological indicators (eg, rheumatoid factors, anti-nuclear antibodies, anti-neutrophilic antibodies), highlighting the immune background of this disease.[18] LGL is particularly associated with autoimmune diseases, especially rheumatoid arthritis (observed in 11% to 36% of cases[19]), other hematological diseases and bone marrow failure syndromes (eg, myelodysplastic syndrome (MDS), paroxysmal nocturnal hemoglobinuria, aplastic anemia, pure red cell aplasia) often co-occur with LGLL. This also suggests widespread immune system dysfunction with LGLL.[18] Therefore, the immune mechanism of LGLL serves as a valuable starting point for the development of more effective and better-tolerated drug combinations and clinical regimens.

## 2.2 Current Treatment Landscape

Patients with LGLL do not always need treatment at the time of diagnosis, and patients with newly diagnosed asymptomatic LGLL are observed rather than treated immediately. LGLL cannot be cured by current treatments, and most patients present an indolent disease course with median survival >10 years. Western countries have reported that two-thirds of patients eventually require treatment indications, primarily due to recurrent infections resulting from severe neutropenia.[20] Treatment is required when patients have symptomatic or life-threatening peripheral blood cytopenia, mainly including (meeting at least 1 of the following conditions):

- absolute neutrophil count (ANC) $<0.5 \times 10^9$/L, or neutropenia with recurrent infections
- hemoglobin <100 g/L, or requiring infusion of red blood cells
- platelets level $<50 \times 10^9$/L
- Concomitant autoimmune diseases requiring treatment
- Symptomatic splenomegaly
- Severe B symptoms (fever of unknown cause with body temperature over 38 °C; night sweats; weight loss of ≥10% within half a year)
- Pulmonary hypertension

Current treatment options for LGLL are limited, because of the lack of a deep and adequate understanding of its pathogenesis and the absence of effective targeted therapies. At the same time, LGLL is a rare disease with a low incidence and insufficient prospective clinical trials, and there are no standard treatment options for

LGLL. Current treatment options for LGLL include immunosuppressive therapies, purine analogues, chemotherapies with cytotoxic drugs, splenectomy, targeted therapies, and hematopoietic stem cell transplantation.

For T-LGLL and CLPD-NK, immunosuppressants, including methotrexate, cyclophosphamide, and cyclosporin A, are still the most recognized first-line regimen.[21-23] The overall response rate of the 3 immunosuppressants were similar. However, irrespective of the type of immunosuppressant, the low complete response rate indicates that current immunosuppressive treatment has reached the maximum efficacy.

The efficacy of immunosuppressants in LGLL patients is summarized below:

| **Product/Regimen** | **Author (Year)** | **Number of Cases** | **ORR, % (n)** | **CR, % (n)** |
|---|---|---|---|---|
| **Methotrexate** | Sanikommu et al (2018)[23] | 34 | 44% (15) | |
| | Bareau et al (2010)[24] | 36 | 44% (16) | 14% (5) |
| | Loughran et al (1994)♯[24] | 10 | 60% (6) | 50% (5) |
| | Loughran et al (2015)♯[25] | 55 | 38% (21) | 5% (3) |
| **Cyclophosphamide** | Sanikommu et al (2018)[23] | 22 | 47% (10) | |
| | Li JY et al (2016)[26] | 36 | 86% (31) | 22% (8) |
| | Moignet et al (2014)[27] | 45 | 72% (32) | 47% (21) |
| | Poullot et al (2014)[28] | 13 | 69% (9) | 46% (6) |
| | Dhodapkar et al (1994)[29] | 16 | 63% (10) | 38% (6) |
| | Sanikommu et al (2018)[23] | 44 | 45% (20) | |
| | Fengkui et al (2020)[30] | 16 | 56% (9) | 31% (6) |
| | Osuji et al (2006)[31] | 14 | 92% (13) | |
| **Cyclosporin A + Prednisone** | Fengkui et al (2020)[30] | 83 | 48% (40) | 18% (15) |

♯Prospective studies

Based on the pathogenesis of T-LGLL, it is speculated that potentially effective novel drugs may include monoclonal antibodies (eg, anti-CD2, CD22, and CD52 antibodies), small molecule-targeted drugs (eg, RAS inhibitors [tipifarnib]), JAK3-specific inhibitors (tofacitinib), PI3K inhibitors and proteasome inhibitors (eg, bortezomib).[32-33] At present, alemtuzumab and tofacitinib are the main applications and shown to be effective. The efficacy and side effects of novel drugs are not yet known, so a larger patient population needs to be involved in clinical trials for further validation. Meanwhile, most new drugs are expensive and inaccessible, rendering them unsuitable as first-line treatments for LGLL patients. Hematopoietic stem cell transplantation and splenectomy are not routine treatments for LGLL and are only considered if a patient is resistant to most drugs.

To sum up, there is currently no standard first-line treatment regimen for LGLL, and addressing the challenge of providing patients with more profound and durable

remission remains a pressing clinical issue. The TPM regimen (thalidomide combined with methotrexate and prednisone) has been employed in our site for the treatment of LGLL since 2013. Preliminary results showed that a total of 20 patients were enrolled, of whom 20 (90.0%) achieved hematological remission and 16 (80.0%) achieved complete remission (CR), demonstrating favorable preliminary efficacy. Grade ≥3 adverse events (AEs) were rare, demonstrating favorable safety. Therefore, we designed this study to observe the efficacy and safety of TPM regimen in patients with symptomatic LGLL.

## 2.3 Investigational Product -Thalidomide

Thalidomide, also known as 2-phthalimidoglutarimide, has been widely used in early pregnancy due to its sedative and antiemetic effects, and later due to its severe teratogenicity, it caused the thalidomide tragedy and was banned from use in many countries. But the study of thalidomide did not stop there. With the continuous exploration of the pharmacological mechanism of thalidomide, it has been found that thalidomide has the effect of anti-angiogenesis and inhibition of tumor necrosis factor alpha (TNF-α), so that is widely used in immune diseases and various malignant tumors.[34-36] At present, thalidomide has been approved for multiple myeloma (MM), erythema nodosum leprosum, graft versus host disease, recurrent aphthous ulcer in HIV infection/Behcet's syndrome, paraneoplastic sweating, paraneoplastic and uremic pruritus, cachexia in HIV and cancer, intractable gastrointestinal hemorrhage, refractory irinotecan-induced diarrhea, discoid lupus erythematosus, rheumatoid arthritis, etc.

**Pharmacokinetics of Thalidomide**

Thalidomide is interconvertible between (R)- and (S)- enantiomers in plasma, with protein binding rates of 55% and 65%, respectively. More than 90% of the absorbed drugs are excreted through urine and feces within 48 hours. Thalidomide is little metabolized in the liver, but it spontaneously hydrolyzes into a variety of renal excreted metabolites.

After a single oral dose of thalidomide of 200 mg (the United States approved capsule form) in healthy volunteers, thalidomide was slowly absorbed and reached a peak concentration ($C_{max}$) of 1 to 2 mg/L 3 to 4 hours after administration, the absorption lag time was 30 minutes, the total exposure (AUCβ) was 18 mg ± h/L, the elimination half-life was 6 hours, and the total clearance rate was 10 L/h. Due to the low solubility of thalidomide in the gastrointestinal tract, the absorption rate of thalidomide was limited, and its elimination rate was faster than the absorption rate.

Multiple doses of thalidomide 200 mg/day for 21 days will not change pharmacokinetics, with a steady-state peak concentration ($C_{ssmax}$) of 1.2 mg/L. Simulated multiple doses of 400 mg/day and 800 mg/day also showed no accumulation, with $C_{ssmax}$ of 3.5 and 6.0 mg/L, respectively. Multiple dose studies in cancer patients showed that pharmacokinetic profile was similar to that in healthy population at similar

doses. At dose range of 50 to 400 mg, thalidomide showed a proportional increase in area under the concentration-time curve with increasing doses. Due to the low solubility of thalidomide, $C_{max}$ is not proportional to the dose, while the time to reach the maximum plasma concentration is prolonged with increasing doses. Age, sex, and smoking have no effect on the pharmacokinetics of thalidomide, and food has little effect. Thalidomide does not change the pharmacokinetics of oral contraceptives and is unlikely to interact with warfarin and grapefruit juice. Since thalidomide is primarily excreted by hydrolysis and passive excretion, pharmacokinetics is unlikely to be changed in patients with impaired liver or kidney function.

**Anti-tumor Mechanisms of Thalidomide**

The exact mechanism of thalidomide as an antineoplastic agent is not fully understood, but some studies have suggested that it may have anti-tumor effects on tumor cells through a variety of pathways. The following are some possible anti-tumor mechanisms: [34, 36]

- Antiangiogenic effect: Thalidomide may reduce the tumor blood supply by inhibiting angiogenesis. It is thought to affect some growth factors associated with angiogenesis, such as vascular endothelial growth factor (VEGF), basic fibroblast growth factor (bFGF), and TNF-α, thereby inhibiting the angiogenesis of tumor cells.
- Immunomodulatory effects: Thalidomide may have an effect on the immune system, enhancing the body's immune response to tumor cells. It may promote the activity of NK cells, improve the immune response of T cells, and reduce the utilization of immune escape mechanisms by tumor cells.
- Anti-inflammatory effect: Thalidomide has anti-inflammatory effects and may affect tumor growth by reducing inflammation in the tumor microenvironment. It can reduce the activity of inflammatory cells and reduce the production of inflammation-related factors.
- Immunoregulatory apoptosis: Thalidomide is thought to induce apoptosis of tumor cells, thereby reducing tumor load. It may achieve this effect by altering cytokine production and cell signaling pathways.

**Thalidomide in the treatment of Hematologic Tumors**

The first in human Phase I clinical study of thalidomide was conducted in the early 1950s. The study was initially conducted on the drug as a sedative and hypnagogue. However, it was later discovered that thalidomide could cause severe congenital malformations, and its use was discontinued. Thalidomide has not been reported in the treatment of LGLL, but it has been used in hematologic tumors, including MM, Waldenström macroglobulinemia (WM), and MDS.

**Study on Thalidomide in the Treatment of Multiple Myeloma**

MM is a disease that cannot be cured despite active treatment such as high-dose chemotherapy and stem cell transplantation. The theoretical basis for treatment with thalidomide is primarily based on the observation of enhanced neoangiogenesis in bone marrow of patients with progressive disease, and the potential anti-angiogenic effects through the TNF-α, basic fibroblast growth factor (bFGF), and vascular endothelial growth factor (VEGF) in preclinical studies. A study team of the University of Arkansas reported for the first time the efficacy and safety of thalidomide monotherapy in refractory MM.[37] Of the 84 evaluable patients who received 200 mg of thalidomide (gradually increased to 800 mg), serum M-protein concentration was reduced by more than half in 25% of the patients and by more than 90% in 8 patients. Two CRs were observed, and these remissions were durable because the median time to progression had not been reached in the 14-month follow-up. Although more than 3 quarters of patients experienced a response consistent with reduced plasma cell infiltration in the bone marrow, the therapy had no effect microvascular density. Further follow-up of 169 patients showed that 2-year event-free survival and overall survival (OS) were 20% and 48%, respectively. In addition, high-dose thalidomide was associated with improved survival in high-risk patients, supporting the opinion of the dose-related effect. The response to thalidomide in patients with MM usually occurs after 1 to 2 months of treatment of 200 to 400 mg daily, a dose of 50 mg daily may be sufficient as a maintenance therapeutic dose for some patients if considering the clinical response.

The main toxicity of thalidomide observed in these patients included neurological events (somnolence, dizziness, delirium, tremor, uncoordinated movement, stinging and numbness), gastrointestinal disorders (constipation, nausea, vomiting and stomatitis) and systemic symptoms (weakness, weight loss, and fever). It is concluded that thalidomide had significant anti-tumor activity in advanced high-risk MM, the clinical data on early single agent were more strongly supported, and based on early favorable results for refractory myeloma, thalidomide was granted as an orphan drug, providing it with a 7-year protective study and development period. CC-5013, an immunomodulatory derivative of thalidomide, has good anti-tumor effects and shows promising results in relapsed and refractory myeloma, with a 25% reduction in M protein concentration in 71% of 24 treated patients.[38] Thalidomide is currently used for the treatment of patients with advanced refractory or newly diagnosed MM, and is also of important value in maintenance therapy.

**Study on Thalidomide in the treatment of Waldenström Macroglobulinemia**

WM is a malignant lymphoplasmacytic lymphoma that may cause symptoms and complications due to infiltration of bone marrow, spleen, or lymph nodes. A Phase II study of thalidomide in WM enrolled 20 patients with a median age of 74 years (range: 48 to 85).[39] Ten of the patients were previously untreated, while the other 10 were previously treated, including 4 who did not respond to any previous course (initially refractory), 5 who relapsed despite chemotherapy (refractory relapse), and 1 who relapsed without treatment. Approximately one-third of patients had at least 1 of severe anemia, splenomegaly, or elevated serum β2-microglobulin. The initial dose of thalidomide was 200 mg orally per day, taken before bedtime. The dose was gradually

increased by 200 mg every 14 days up to a maximum dose of 600 mg depending on patient tolerance. Five of the 20 patients (25%) achieved partial remission (PR), defined as a reduction of >50% in tumor infiltration at all involved sites. Five patients were rated as stable disease (SD), while 10 showed early disease progression (PD) with shorter remission duration, ranging from 0.8 to 2.8 months. PR was observed in 3 of 10 previously untreated patients and 2 of 10 previously treated patients. Of the 5 patients treated during refractory relapse and 7 patients treated for more than 24 months, none responded to thalidomide. For all patients, the median time to progression was 5 months and the median duration of response (mDoR) was 11 months.

Thalidomide has a variety of side effects, such as peripheral neuropathy, constipation, and nausea. These side effects are more commonly reported in patients over 70 years of age. Therefore, the dose of thalidomide was gradually increased to the target dose of 600 mg in only 5 patients. Four patients only received a maximum dose of 200 mg, and 11 patients received a maximum dose of 400 mg. Ten patients were treated with thalidomide for 2 months, 3 of whom discontinued thalidomide within 30 days of the first administration. The main cause for lowering the maximum dose of thalidomide or early discontinuation of treatment was intolerance.

**Study on Thalidomide in the Treatment of Light Chain Amyloidosis**

Thalidomide has also been used in some patients with AL amyloidosis. In one study, a significant reduction in urine M-protein was observed in 25% of patients. Grade 3 or 4 toxicity occurred in 50% of patients, and thalidomide was discontinued due to side effects in 25% of patients. Fatigue and other central nervous system toxicities were major dose-limiting toxicities.[40]

In another study, dexamethasone was used in combination with thalidomide at a starting dose of 100 mg daily in increments of 100 mg every 2 weeks up to 400 mg. Decreased M-protein and more than 50% reduction in proteinuria were observed in 59% of all affected patients. Serious side effects included bradycardia, which led to treatment interruption in 65% of patients. Six patients receiving a dose of 400 mg responded to treatment, while none of the 4 patients receiving 100 mg daily responded to treatment.[41]

**Study on Thalidomide in the Treatment of Myelofibrosis**

Myelofibrosis with myeloid metaplasia (MMM) is a proliferative disorder accompanied by myelofibrosis, ectopic hematopoiesis, and increased microvessel density. Several Phase II studies have evaluated the efficacy and tolerability of thalidomide in MMM.[42-45] Improvements in thrombocytopenia and anemia appeared to be the main benefits from the treatment. Significant spleen shrinkage was observed in less than 20% of patients. Adverse hematological effects, such as marked leukocytosis and thrombosis, were observed in some patients. In addition, up to 50% of patients discontinued thalidomide due to side effects. Therefore, multiple studies have confirmed that thalidomide was associated with clinical benefit in patients with MMM, but its tolerability was a major concern in this disease. A Phase II trial conducted by Mesa et

al investigated the effect and tolerability of low-dose thalidomide and prednisone in patients with MMM.[46] Twenty-one patients received 50 mg of thalidomide daily along with oral prednisone for 3 months. Thalidomide and prednisone were well tolerated, with a treatment response in 13 patients (62%). Of the 10 patients requiring blood transfusion, 7 improved and 4 no longer required blood transfusion. Six of the 8 patients with thrombocytopenia had platelet counts increased by more than 50%; and of the 21 patients, 4 had spleen shrinkage by more than 50%. Low doses of thalidomide and prednisone appeared to be more tolerated, meanwhile improving therapeutic effects. Thalidomide and prednisone need to be further evaluated in the early stages of MMM. This combination is a reasonable treatment option for patients with MMM who mainly present with cytopenia. Two long-term outcome analysis trials conducted by the Mayo Clinic, which included 36 patients, showed that thalidomide monotherapy or in combination with prednisone achieved an overall response in 19 patients (53%). In addition, 19% of patients remained in remission without additional medication for a median of 17 months after discontinuation of thalidomide.[47]

**Study on Thalidomide in the Treatment of Myelodysplastic Syndrome**

The immunomodulatory and antiangiogenic effects of thalidomide have prompted the evaluation of its use in the treatment of MDS. In a large-scale study, Raza et al treated 83 MDS patients with thalidomide at a starting dose of 100 mg/day, which was gradually increased to a maximum of 400 mg/day, if tolerated. Fifty-one patients completed 3 months of treatment with an overall response rate (ORR) of 19%; hemoglobin rebounded in 15 patients, 10 no longer required blood transfusion, and 1 patient had increased platelets. The mDOR was 306 days. Patients who were more likely to benefit were those with lower primitive naïve cells and higher baseline platelet counts.[48] Thalidomide was administered to 30 patients with MDS in a study conducted by Zorat et al. Hemoglobin rebounded in 10 patients, and 6 no longer required blood transfusion. Clinical responses were more common in patients with higher baseline platelet counts and lower percentage of outbreaks.[49] Stupp et al treated 34 patients and observed hematological improvements in 56% of the patients. The quality of life was improved in responders.[50]

**Study on Thalidomide in the Treatment of Acute Myeloid Leukemia**

Angiogenesis is involved in the pathophysiology of acute myeloid leukemia (AML). Twenty AML patients with poor prognosis received 200 to 400 mg of thalidomide daily. Four of these patients achieved a PR, defined as decreased bone marrow infiltration in leukemia with improvement in red blood cell and platelet counts.[51] A thalidomide trial of 16 patients with refractory or relapsed AML showed that 1 patient achieved CR for 36 months and two patients had transient reductions in bone marrow outbreaks. There was no correlation between the reduced levels of angiogenesis and response.[52] Therefore, thalidomide is not recommended in patients with AML outside of clinical trials.

## 2.4 Investigational Product - Methotrexate

Methotrexate, commonly referred to as MTX for short, is widely used agent in the medical field with a variety of uses, including anti-cancer therapy, treatment of autoimmune diseases, and the control of inflammatory diseases. Details of methotrexate are provided as follows:

**Pharmacokinetics of Methotrexate**

In the treatment of inflammatory autoimmune diseases, methotrexate is usually taken orally once a week. In clinical practice, treatment typically starts at a dose of 10 mg per week and is increased by 5 mg every 2 to 4 weeks, up to a maximum dose of 20 to 30 mg per week, which will vary depending on patient's clinical response and tolerance. In recent years, there is increasing interest in the injectable use of methotrexate, especially in the subcutaneous form, which has greater benefits than oral administration. Compared with the oral form, subcutaneous injection of methotrexate showed better clinical efficacy and tolerability. Subcutaneous injection of methotrexate is currently recommended in cases of where oral methotrexate is ineffective or poorly tolerated. In juvenile idiopathic arthritis, methotrexate has shown to be effective at doses of 10 to 20 $mg/m^2$ body surface area.

After oral administration of methotrexate, its absorption occurs in the proximal jejunum, mainly through the proton-coupled folate transporter (PCFT/SLC46A1), which transports reduced folic acid and methotrexate. In addition, part of methotrexate may be metabolized to 4-amino-4-deoxy-N10-methylpara aminobenzoic acid with the involvement of intestinal bacteria. The bioavailability of methotrexate is relatively high, generally in the range of 64% to 90%, but varies widely between patients and decreases with increasing doses, with stable bioavailability at doses greater than 15 mg per week, indicating intestinal transporter saturation. Methotrexate, when taken orally, has a $C_{max}$ of between 0.3 to 1.6 µmol/L, generally occurring 0.75 to 2 hours after administration. Multiple studies have demonstrated that subcutaneous injection of methotrexate has higher bioavailability compared with oral methotrexate. Subcutaneous injection of methotrexate resulted in linear, dose-proportional increases in blood drug concentrations, with no dose saturation effect. Approximately 50% of circulating methotrexate binds to plasma protein. Methotrexate can be distributed into synovial fluid at concentrations comparable to those in plasma. Methotrexate undergoes the first-pass effect metabolism in the liver and is converted to 7-hydroxymethotrexate (7-OH-MTX), a major metabolite of methotrexate. Renal excretion is the main route of excretion for methotrexate. Methotrexate is filtered through the glomeruli, as well as active tubular secretion and reabsorption. A small portion of methotrexate is excreted through bile and a portion enters hepatic-enteric circulation. The plasma half-life of low dose of methotrexate is between 4.5 to 10 hours. The excretion of methotrexate is reduced in patients with impaired renal function, ascites, or pleural effusion, who need to be particularly careful to monitor toxic effects and may need to reduce the dose or in some cases discontinue methotrexate therapy.

Cellular uptake of methotrexate is mediated by reduced folate carrier 1 (RFC1/SLC19A1), with limited contributions from α and β folate receptors. The

outflow of intracellular methotrexate is regulated by ATP-binding cassette transporters (ABCC), which transport many drugs and chemotherapeutic agents. Intracellularly, part of intracellular methotrexate is converted to methotrexate polyglutamate under the catalysis of folate polyglutamate synthase, which adds up to 7 glutamic acid residues to methotrexate. Methotrexate derivatives with more than 3 glutamic acid residues are not substrates of ABCC and therefore have greater retention ability in cells. Polyglutamylation can be reversed by a deglutamylation process catalyzed by gamma-glutamyl hydrolase, resulting in steady-state intracellular methotrexate levels. After oral administration, methotrexate polyglutamate is found in red blood cells, neutrophils, monocytes, hepatocytes, and synovial cells. The accumulation of intracellular methotrexate polyglutamate leads to sustained efficacy and allows weekly administration of methotrexate, despite its relatively short plasma clearance half-life.

**Clinical Experience with Methotrexate**

Methotrexate is widely used in the treatment of patients with rheumatoid arthritis (RA), and in a subset of RA patients, it is closely related to LGLL. LGLL exhibits activation of multiple survival signaling pathways, among which the JAK/STAT pathway has shown to be involved with LGL transformation.[53] Low-dose methotrexate has anti-inflammatory and immunosuppressive mechanisms that can effectively inhibit the JAK/STAT pathway.[54] As a standard first-line treatment, methotrexate has good efficacy in patients with neutropenia and autoimmune diseases. At the same time, methotrexate has been successful in treating patients with neutropenia caused by Felaty syndrome, while patients with LGLL often present with neutropenia. Therefore, in 1994, Loughran et al reported for the first time the results of prospective clinical trials on methotrexate of T-LGLL.[55] Ten patients were treated with methotrexate at 10 mg/m$^2$ per week in this study, some of whom also received prednisone. Five of these patients achieved a complete hematologic remission, 3 of them achieved a molecular response with the disappearance of T-cell clones. The mDOR was 3.8 years.

In a large series of studies in 2010 which involved 62 patients, the ORR was 55% in 36 patients treated with methotrexate, and a large percentage of patients relapsed after treatment. Twelve of the 18 patients (67%) relapsed after more than one year follow-up. The onset time was 2 to 12 weeks.[56] In general, methotrexate could be continued indefinitely due to its good tolerability. Neutropenia gradually reappeared even in patients who achieved a complete molecular response.

In 2015, Loughran et al reported the results of the second T-LGLL prospective Phase II clinical study.[25] The study enrolled 55 patients who received oral methotrexate at 10 mg/m$^2$ per week as initial treatment, and those who failed the methotrexate treatment were eligible for cyclophosphamide with 100 mg orally daily. The ORR of methotrexate was 38%, and the complete remission rate (CRR) was 5%. For patients with neutropenia, the ORR was 42%. The ORR in patients with anemia was 34%. Serum biomarker studies confirmed the inflammatory environment of LGL, but LGL is not a predictor of response. Also, response-related gene expression characteristics were confirmed, and it was speculated that gene expression was driven by STAT3

mutations, while methotrexate as an immunosuppressant was a suitable and effective treatment option.

## 2.5 Rationale for Thalidomide Combined with Methotrexate and Prednisone in the Treatment of LGLL

Methotrexate is one of the main therapeutic agents for LGLL at present. The ORR of single drug treatment for LGLL is 40% to 60%, but the CRR is low which is usually less than 10%. As an indolent lymphoma, disease recurrence is inevitable. How to improve the CRR, progression-free survival (PFS), OS, and improve the quality of life are still difficult issues at present.

Although there is currently no direct evidence of thalidomide combined with methotrexate and dexamethasone, a retrospective study in 2007 reported oral regimens using cyclophosphamide, thalidomide, and dexamethasone (CTD) or attenuated CTD (CTDa) in patients with AL amyloidosis.[57] A total of 75 advanced patients (including 44 patients with clonal relapse after prior treatment) were enrolled, of whom 51 (68%) received CTD and 24 (32%) received CTDa. Of the 65 evaluable patients, 48 (74%) had a hematologic response, of whom 14 (21%) had a CR and 34 (53%) had a PR. The median estimated OS from the start of the treatment was 41 months, and the OS from diagnosis was not reached with a median follow-up time of 22 months. The 3-year estimated OS for complete and partial hematological responders was 100% and 82%, respectively. The incidence of toxicity requiring treatment to be discontinued was 8%, and 52% of patients had ≥Grade 2 events. The TRM was 4%. The clonal response rate to CTD reported was higher than that previously reported for AL amyloidosis non-transplant regimen, and it allows the use of CTD in patients at low risks. Prospective randomized study of CTD is needed.

A Phase II clinical study showed that thalidomide combined with cyclophosphamide and dexamethasone had good efficacy and safety in patients with idiopathic Castleman's disease.[58] A total of 25 patients were enrolled, and patients received thalidomide with 100 mg before bed for 2 years. Cyclophosphamide was taken orally at 300 mg/m$^2$ on Days 1, 8, 15, and 22, 4 weeks as a cycle for a total of 1 year. Prednisone was given at 1 mg/kg on Days 1 and 2, 8 and 9, 15 and 16, 22 and 23, 4 months as a cycle for a total of 1 year. This regimen lasted for 2 years or until treatment failure. One patient in this study died of pulmonary infection and 1 developed a Grade 3 rash. There were no other AEs with Grade ≥ 3. Grade 1 or 2 constipation (40%), pruritus (20%), rash (16%), peripheral sensory neuropathy (16%), and nausea (16%) were the most common AEs. Other AEs included irregular menstruation (12.5% in female patients), glucose intolerance (12%), alanine aminotransferase increased (12%), pneumonia (12%), sexual hypoactivity (12%), leukopenia (8%), peripheral neuropathy (motor) (8%), erectile dysfunction (5.8% in male patients), neutropenia (4%), and hepatitis B virus reactivation (4%). No thrombotic events were observed and no hemorrhagic events were reported during follow-up. The results suggested that

thalidomide combined with immunosuppressants and glucocorticoids was a safe combination.

Since 2013, the Institute of Hematology and Blood Diseases Hospital, Chinese Academy of Medical Sciences has been using TPM regimen to treat LGLL, ie, thalidomide of 50 to 100 mg/night, prednisone of 0.5 to 1.0 mg/kg every other day (QOD), and methotrexate of 10 mg/m$^2$/week. The regimen will continue for up to 12 months until PD or intolerance. Preliminary results showed that a total of 20 patients were enrolled, of which 20 (90.0%) achieved hematological remission, 16 (80.0%) achieved CR, demonstrating favorable preliminary efficacy. Grade ≥3 AEs were rare, and the regimen had good safety profile. The preliminary data showed that TPM had a high effective rate and good safety and tolerability in the treatment of LGLL.

Thalidomide combined with immunosuppressants and hormones showed combined efficacy in other hematological tumors, and adverse reactions did not increase significantly demonstrating good safety profile. The preliminary data of this regimen in LGLL also proved its efficacy and safety, thus, this study further explores the TPM combination therapy for LGLL.

## 2.6 Risk/Benefit Assessment

### 2.6.1 Investigational Plan

This is a prospective multicenter Phase II clinical study employing an oral regimen administered in 3-month cycles. Patients will receive thalidomide of 50 to 100 mg once daily (QD) before bedtime, prednisone of 0.5 to 1.0 mg/kg QOD, methotrexate of 10 mg/m$^2$/week for up to 4 cycles. Prednisone will be tapered after 3 months of dosing and dosed for up to 6 months. Patients who achieve CR can discontinue methotrexate after 4 months of consolidation therapy, with a maximum duration of 1 year. For patients who achieve PR or CR, thalidomide will be dosed for up to 2 years or discontinued at the investigator's discretion based on clinical symptoms.

Efficacy evaluation will be conducted at the end of each cycle starting from Cycle 1. The efficacy evaluations will continue until PD, discontinuation of treatment (for patients who continue treatment after PD), initiation of a new anti-cancer therapy, or withdrawal from the study (eg, death, patient's request, loss of follow-up, etc.), whichever occurs first.

The primary objective of this study is to evaluate the efficacy and safety of TPM regimen and further explore the biomarkers associated with clinical efficacy.

### 2.6.2 Potential Risks

According to the preliminary summarized clinical safety data, the adverse reactions of thalidomide combined with immunosuppressants and hormones regimen were mainly gastrointestinal reactions and peripheral neuropathy, manifested as numbness of hands and feet, loss of appetite, constipation, dizziness, etc. However, the adverse reactions were mainly Grade 1 or 2 and could be managed. The most severe toxicities of thalidomide were embryo-fetal toxicity and venous thromboembolism. Due to the risk

of embryo-fetal toxicity and venous thrombosis. Pregnant and lactating women will be excluded from the study and those who become pregnant during the study will be promptly withdrawn from the study. Due to the risk of venous thromboembolism, high-risk patients based on the Caprini score for venous thromboembolism will be excluded from this study. Patients will take aspirin concurrently with oral thalidomide to prevent venous thromboembolism and coagulation function will be monitored at the end of each cycle. The preliminary data of this study showed that except for 1 Grade 3 nausea, no other Grade ≥3 adverse reactions occurred and all adverse reactions were clinically manageable. The safety of thalidomide combined with methotrexate and prednisone will be monitored in this study.

### 2.6.3 Potential Benefits

Both prospective and retrospective data indicated that the current immunosuppressive treatment had a low CRR in patients with LGLL, and the current immunotherapy has reached the "ceiling". Addressing the challenge of providing patients with more profound and durable remission remains a pressing clinical issue to be solved. LGLL is an immune disorder, immunosuppression alone is not the best choice. As an immune modulator, thalidomide combined with immunosuppressants can enhance the body's immune response to tumor cells, promote the activity of NK cells, improve the immune response of T cells, reduce the utilization of immune escape mechanisms by tumor cells, and be more targeted for the treatment of LGLL. At the same time, previous data showed that the TPM regimen had a high effective rate and CRR, far exceeding the current immunosuppressants. In addition, thalidomide combined with immunosuppressants and hormones showed good safety in hematologic tumors. Therefore, it is predicted that the TPM regimen in the treatment of LGLL can further improve the effective rate and deep response rate in patients with LGLL while ensuring the safety of patients.

### 2.6.4 Assessment of Potential Risks and Benefits

Thalidomide combined with immunosuppressants and hormones has been used in a variety of hematologic treatments. The adverse reactions are mainly gastrointestinal reactions and peripheral neuropathy, with sufficient safety information and manageable risks. Previous data showed that TPM regimen had a good effective rate and CRR in patients with LGLL. In this study, the adverse reactions of patients during treatment will be closely monitored and treated in a timely manner until they return to normal or reach clinical stability.

Therefore, the risk of this study is manageable, and the expected benefits outweigh the risks.

# 3. Objectives

**Primary Objective:**

- To evaluate the CRR of symptomatic LGLL patients treated with TPM regimen

**Secondary Objectives:**

- To evaluate the safety and tolerance of symptomatic LGLL patients treated with TPM regimen
- To evaluate the ORR and PR rate of symptomatic LGLL patients treated with TPM regimen
- To evaluate the PFS and OS of symptomatic LGLL patients treated with TPM regimen
- To evaluate the duration of response (DoR) in symptomatic LGLL patients treated with TPM regimen

**Exploratory Objective:**

- To explore biomarkers significantly and potentially associated with clinical efficacy

# 4. Study Design

## 4.1 Overall Study Design

This is a prospective multicenter Phase II clinical study employing an oral regimen administered in 3-month cycles. Patients will receive thalidomide of 50 to 100 mg QD before bedtime, prednisone of 0.5 to 1.0 mg/kg QOD, methotrexate of 10 mg/m$^2$/week for up to 4 cycles. Prednisone will be tapered after 3 months. Patients who achieve CR can discontinue methotrexate after 4 months of consolidation therapy, with a maximum duration of 1 year. For patients who achieved a partial remission (PR) or CR, Thal maintenance therapy was recommended to be continued for a maximum duration of two years, but also depended on patients or investigator's choices.

Efficacy evaluation will be conducted at the end of each cycle starting from Cycle 1. The efficacy evaluations will continue until PD, discontinuation of treatment (for patients who continue treatment after PD), initiation of a new anti-cancer therapy, or withdrawal from the study (eg, death, patient's request, loss of follow-up, etc.), whichever occurs first.

The primary objective of this study is to evaluate the efficacy and safety of TPM regimen and further explore the biomarkers associated with clinical efficacy.

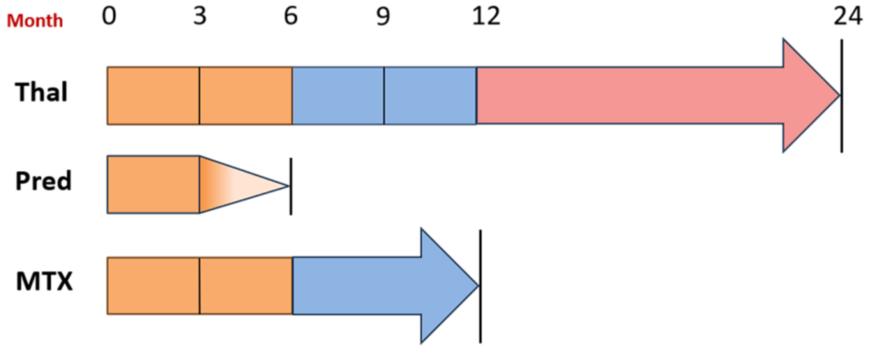


Abbreviations: CR, complete remission; MTX, methotrexate; Pred, Prednisone; Thal, thalidomide.

Notes:

Thal: 50 to 100 mg, oral, before bed, for 2 years;

Pred: 0.5-1 mg/kg, oral, every other day, could be tapered after 3 months of dosing and dosed for up to 6 months

MTX: 10 mg/m$^2$, once weekly, patients who achieve CR can discontinue MTX after 4 months of consolidation therapy, with a maximum duration of 1 year.

## 4.2 Description of Overall Study Design and Plan

The study consists of a 28-day screening period, a treatment period (up to half a year for prednisone, up to 1 year for methotrexate, and 2 years for thalidomide), and a follow-up period (safety and efficacy follow-up). Patients who provide the signed

informed consent will undergo baseline examinations during the screening period. Prior treatment and efficacy will also be evaluated before enrollment. Patients who met the inclusion/exclusion criteria will enter the treatment period. Relevant examinations specified in the protocol will be completed for all patients during treatment to observe efficacy, safety, and hemogram changes after discontinuation of the study drugs. The follow-up period will begin after the end of the treatment period.

## 4.3 Duration of Treatment

Patients enrolled in this study will be treated with thalidomide combined with methotrexate and prednisone for 3 months and then evaluated for efficacy. Based on the efficacy evaluation, patients will either continue treatment as per the initial regimen or be withdrawn from the study. The Institute of Hematology and Blood Diseases Hospital, Chinese Academy of Medical Sciences, is a specialized hospital for hematology, where treats 30 to 40 LGLL patients every year, of which approximately 5 to 10 are eligible for enrollment. A total of 5 to 10 patients are expected to be enrolled in multiple centers nationwide every year. The enrollment period of this study is expected to span 2 years. This study is of a limited course of treatment. Patients who obtain sustained clinical benefits from the study treatment without consent withdrawal, PD, and intolerable toxicity may continue the study and receive follow-up. The end of study (EOS; study completion) is defined as the time when the last patient completes the last visit.

## 4.4 Follow-up

Patients who withdraw from the study due to intolerable AEs will be followed up in this study until the events resolve to Grade 1 or lower, or the AE is stable. Patients will be followed up for up to 24 months after completion of the treatment, or until study termination, PD, initiation of new anti-tumor therapy, or death.

## 4.5 Sample Size

A retrospective study of 45 patients is used as the historical control, in which cyclophosphamide monotherapy was applied with an effective rate of 72% and CRR of 47%. From the perspective of clinical expertise, taking the CRR of 70% as the minimum threshold for clinical significance, the sample size of 35 patients in this group is deemed sufficient to test the overall true CRR that is superior to that of the historical control at a false positive rate $\alpha = 0.05$ (two sides) and false negative rate $\beta = 0.2$ (ie, power = 80%). With a 20% dropout rate, at least 42 patients would be required to enroll in this study.

## 4.6 Steering Committee

A Steering Committee will be established for this study. The Steering Committee will consist of investigator participating in the study, an additional study physician, and a

statistician. Study design and inclusion/exclusion/withdrawal criteria will be determined by the Steering Committee.

Members of the Steering Committee will periodically review the status of enrolled patients and adjudicate on situations not covered by existing inclusion, exclusion, or withdrawal criteria.

## 4.7 Independent Data Monitoring Committee

The Independent Data Monitoring Committee (IDMC) will review the trial data to monitor the quality of trial operations, including the protocol compliance, recruitment status, dropout rate, and data integrity. The IDMC will monitor the accumulated patient safety data every 1 month upon request. The IDMC may propose recommendations on adjustment of the ongoing trial design on the premise of ensuring the integrity of the trial.

## 4.8 Study Termination

EOS is defined as the last patient last visit at the end of the follow-up period, which will be the last data collection point and the last patient may be an outpatient or laboratory sample. EOS is expected to occur approximately 2 years after enrollment of the last patient. A patient will be considered to have completed the study if he/she dies or meets any of the withdrawal criteria.

# 5 Study Population

Eligible patients will be screened within 28 days prior to the administration of the study drugs. Refer to Section 5.5, Screen Failures, for conditions that allow any screening procedure to be repeated.

The inclusion and exclusion criteria for patients enrolled in this study are summarized below. If there is any doubt about these criteria, the investigator must consult with the Sponsor representative and resolve all issues prior to study enrollment. No exemptions are allowed.

## 5.1 Eligibility Criteria for Prescreening

1. Sign pre-screening informed consent form (ICF).
2. Willing to provide a tumor tissue sample (archived or recently collected) for tumor genetic analysis.

## 5.2 Inclusion and Exclusion Criteria

### 5.2.1 Inclusion Criteria

1. Patients who fulfill the diagnostic criteria for T-LGLL or CLPD-NK specified in the 2016 WHO Classification of Lymphoid Neoplasms [1]
2. Patients are fully informed about the study and willing to participate in the study and provide written ICF
3. Male or female, aged ≥18 years
4. Treatment-naive patients, or patients treated with or relapsed from a non-methotrexate/ thalidomide-based regimen
5. Patients with at least one of the following indications for LGLL treatment:
    a. ANC <0.5 × $10^9$/L, or neutropenia with recurrent infections
    b. Hemoglobin <100 g/L, or requiring infusion of red blood cells
    c. Platelets level <50 × $10^9$/L
    d. Concomitant autoimmune diseases requiring treatment
    e. Symptomatic splenomegaly
    f. Severe B symptoms (fever of unknown cause with body temperature over 38 °C; night sweats; weight loss of ≥10% within half a year)
    g. Pulmonary hypertension
6. Patients with Eastern Cooperative Oncology Group (ECOG) score of 0 to 2 (Appendix 1)
7. Patients with life expectancy ≥6 months.

### 5.2.2 Exclusion Criteria

1. Patients not diagnosed with T-LGLL or CLPD-NK
2. Patients with no indication for LGLL treatment
3. Patients who are unable to understand or follow study procedures
4. Patients who have been diagnosed or treated for malignancies other than LGLL within the past five years
5. Patients with non-lymphoma-related hepatic and renal impairment: alanine aminotransferase (ALT) > 3 × upper limit of normal (ULN), aspartate aminotransferase (AST) >3 × ULN, total bilirubin > 2 × ULN, and serum creatinine clearance (CrCl) <30 mL/min
6. Patients with other severe diseases that have an impact on this study (uncontrolled diabetes, stomach ulcers, other severe cardiopulmonary conditions, etc.), at the discretion of investigators.
7. High-risk patients based on the Caprini score for thromboembolism (Appendix 2)
8. Patients with a known history of HIV infection, or with active hepatitis B virus (HBV) infection, or any uncontrolled active systemic infection requiring intravenous antibiotics

Notes: HBV infection is considered active if all following criteria are met: a. HBV DNA quantification ≥2000 IU/mL; b. ALT ≥2 times × ULN; c. LGLL, medications, and other causes-induced hepatitis are excluded. Patients with active HBV infection at the time of initial diagnosis who are converted to inactive HBV infection after adequate anti-HBV treatment could be enrolled in this study.

9. Patients who have undergone major surgery (excluding lymph node biopsy) within the past 14 days or who are expected to undergo major surgery during study treatment
10. Pregnant or lactating women, and women of childbearing potential who have not taken contraceptive measures
11. Patients who are allergic to the agents or the ingredients

## 5.3 Withdrawal Criteria

1. Patients withdraw the ICFs
2. Patients experience PD or death
3. Patients experience intolerable toxicity that cannot be relieved after symptomatic treatment
4. Patients are unwilling to continue treatment
5. There was no clinical benefit for more than half a year
6. Delayed dosing for more than 4 weeks and patients are judged by investigators to be unsuitable for further dosing

7. Pregnancy

8. Lost to follow-up

9. Treatment should be discontinued based on the best interests of the patients at the investigator' discretion

10. Patients does not comply with the protocol, and the investigator deems it necessary to discontinue the treatment

11. The collaborator or regulatory authority notifies to end the clinical study.

If treatment discontinuation is due to any of the above reasons, the investigator must notify the Principal Investigator and complete the case summary page in the case report form (CRF), along with the date and reason for the treatment discontinuation. If treatment discontinuation is due to an AE, the AE will be followed up until it is resolved and stable.

## 5.4 Removal of Patients from Therapy or Assessment

Efforts must be made to complete the efficacy and safety tests at the time of treatment discontinuation as specified in the protocol, and to complete safety follow-up, with complete documentation of AEs and outcomes. The investigator may recommend or provide new or alternative treatments to the patient according to the actual conditions. Patients with non-PD should continue to be followed up for efficacy evaluation until they start a new antineoplastic therapy or have PD.

If a patient refuses to visit the study site for further visits, he/she should continue to be followed up for the collection of information about the study unless he/she withdraws consent to disclose further information or be contacted. In this case, no further evaluation should be conducted and no further information should be collected.

## 5.5 Identification Codes, Enrollment, and Screening Log of Screening Failed Patients

Patients who meet the screening failure criteria may be rescreened. For abnormal screening values leading to exclusion during the screening period, it is allowed to retest (to reassess eligibility), and discuss with the Sponsor as appropriate. The last test result obtained prior to the first dose of the study drugs will be used to determine if a patient is eligible for the study. Measurements taken closest to the first dose of the study drugs (prior to the first dose) will be used as baseline values for safety assessments and treatment decisions.

The investigator will fill out the patient identification code and enrollment log to facilitate easy identification of each patient during and after completion of the study. The completeness of this document will be reviewed by the Sponsor's site contact. Patient identification codes and enrollment logs are confidential documents and will be kept by the investigator in the study documentation. To ensure the confidentiality of

patient's information, this document should not be copied. For all study-related reports and correspondences, patients will be identified by patient identification code and age on the initial ICF. If a patient is not enrolled in the study, the date when the patient is met and the age on the initial ICF will be used.

# 6 Treatments

## 6.1 Treatment Regimen

Treatment will be provided in 3-month cycles. Patients will receive thalidomide of 50 to 100 mg orally QD before bedtime, methotrexate of 10 mg/m$^2$ orally once weekly, and prednisone of 0.5 to 1 mg/kg orally QOD. Prednisone will be tapered after 3 months of dosing and dosed for up to 6 months. Methotrexate will be dosed for up to 1 year. Thalidomide will be dosed for up to 2 years or discontinued at the investigator's discretion based on clinical symptoms. Efficacy was evaluated after 3 months of treatment, if PR or above response to treatment, the initial treatment regimen will be continued. If there is no clinical benefit after 6 months of initial treatment, patients will switch to the second-line treatment was and will be withdrawn from the study.

Dosage and administration for study drugs are shown in the following table:

| TPM Regimen | Dosage and Administration |
|---|---|
| Methotrexate | 10 mg/m$^2$ orally, once weekly |
| Prednisone | 0.5–1 mg/kg orally, every other day |
| Thalidomide | 50–100 mg orally, once daily before bedtime |

## 6.2 Administration of Study Drugs

**Thalidomide:**

Thalidomide administration will be started at 50 mg QD to observe whether there are adverse reactions such as rash and numbness of extremities, and then increased to 100 mg QD, if tolerated, for 1 week. Attention will be paid to observe adverse reactions such as peripheral neuritis. Thalidomide administration will be maintained for a total of 2 years and whether to discontinue will be determined based on the clinical symptoms. If the patient has a study drug-related adverse reaction, the dosage will be adjusted according to the dose adjustment guidelines in Section 7.4.1.

**Methotrexate:**

Methotrexate will be administered orally at 10 mg/m$^2$ once weekly on a fixed day in the morning. Patients who achieve CR can discontinue methotrexate after 4 months of consolidation therapy, with a maximum duration of 1 year.

**Prednisone:**

Prednisone will be administered orally at 0.5 to 1 mg/kg QOD for up to 6 months. It is recommended to take prednisone before 9:00 AM, with food or milk, to reduce stomach irritation. Take antacid between meals to help prevent peptic ulcer. The dose of oral prednisone will be tapered by 5 mg every 2 weeks after Months 3, and 5 mg every week after Month 4 until discontinuation.

## 6.3 Concomitant Medications and Supportive Care

### 6.3.1 Prophylactic Medications

Patients will be instructed not to take any additional medication (including over-the-counter drugs) during the study without consultation with the investigator. At each visit, the investigator will ask the patient about any new medication he/she is taking or has taken since the start of the study drug.

When combined with thalidomide, aspirin will be taken orally at 100 mg QD for antiplatelet therapy to prevent thrombosis.

For all patients for long-term use of low doses of methotrexate, folate supplementation (1 mg/day) is recommended to use to reduce the risk of some common methotrexate toxicities and the possibility of treatment discontinuation. The dosage of folate can be increased as needed to a maximum of 5 mg/day, depending on the reported residual symptoms. Folinate can be used to replace folate. Daily supplementation with folic acid or weekly supplementation with folinate can also prevent increases in plasma homocysteine concentration, which will be induced by methotrexate treatment without supplementation.

When combined with prednisone, calcium carbonate will be administered orally at 1 g QD and calcitriol will be administered orally at 0.25 μg twice daily to prevent osteoporosis; at the same time, proton pump inhibitor will be administered orally to inhibit gastric acid secretion and prevent gastrointestinal reactions.

Patients with positive hepatitis B surface antigen should receive routine examination of the HBV DNA copy number and be administered with anti-hepatitis B therapy (lamivudine or entecavir). Treatment in the active phase of the hepatitis B virus should be avoided. During treatment and within 6 months after treatment, lamivudine or entecavir should be administrated for prophylaxis, and hepatitis B DNA copy number quantification should be monitored during treatment (once every 1 to 2 months).

### 6.3.2 Permitted Medications

The investigator may prescribe any concomitant medication or therapy deemed necessary to provide adequate supportive treatment throughout the study, including those listed below:

Standard supportive treatment (antiemetics, antidiarrheals, anticholinergics, spasmodic drugs, antipyretics, antihistamines, analgesics, and other medications designed to treat the symptoms or signs) will be administered as clinically indicated according to local standard of care and when deemed necessary by the investigator.

- Growth factor support, erythropoietin, and blood product transfusion are allowed for the treatment of symptoms or signs of neutropenia, anemia, or thrombocytopenia according to local standard of care.
- Infectious complications with documented evidence should be treated with oral or intravenous antibiotics, or other anti-infective drugs that the investigator considers appropriate for the infectious condition according to local standards

of care. If clinically indicated, the investigator may administer prophylactic anti-infective therapy to patients according to local standards of care.

- Prevention of tumor lysis syndrome (TLS): For patients with elevated risk of TLS such as high tumor load and renal impairment, adequate hydration and alkalinization should be performed during treatment according to clinical practice, and blood chemistry should be closely monitored.
- Prevention and treatment of HBV activation.
- Other medications for concomitant diseases: Treatment for hypertension, diabetes, etc.
- If a patient experiences an AE during study treatment, the investigator should provide appropriate treatment according to clinical practice, including but not limited to antiemetics, hepatoprotective drugs, drugs for increasing leukocyte, transfusion/blood products, anti-infection drugs, and drugs causing QTc prolongation.

### 6.3.3 Prohibited Medications

The following medications are prohibited during the study. The clinical research associate (CRA) must be notified in advance (or as soon as possible thereafter) of any circumstances requiring prohibited or restricted therapy. In case of any queries or concerns about the use of any of the following prohibited or restricted medications, the investigator must consult the CRA for guidance.

- Any anti-cancer chemotherapy, immunotherapy, targeted therapy, experimental therapy, or hormone therapy (except for therapy with luteinizing hormone-releasing hormone). However, supportive therapeutic drugs, including bisphosphonates and hormone replacement drugs, are allowed.
- Hormonal contraceptives: Hormonal contraceptives can enhance the thrombotic effect of thalidomide.
- Azelastine (nasal): Thalidomide tablets can enhance azelastine's depressant effect on the central nervous system.
- Ethanol: Concomitant administration with methotrexate tablets will increase the risk of drug reactions and liver toxicity, and long-term use may cause liver damage.
- Kanamycin: Concomitant administration with methotrexate tablets will affect its therapeutic effect and may increase the risk of other reactions, such as nausea, vomiting, diarrhea, and other gastrointestinal symptoms.
- Triamterene: Concomitant administration with methotrexate tablets will lead to increased serum concentration, increase toxicity and side effects, and also cause renal excretion disorders, resulting in kidney injury. Methotrexate tablets need to be administered under the guidance of the investigator.

- Carbonic anhydrase inhibitor: Long-term use of carbonic anhydrase inhibitor in combination with prednisone may lead to hypocalcemia, osteoporosis, etc. which should be avoided.
- COVID-19 vaccines (adenovirus vector, messenger RNA), vaccines (inactivated): The immune efficacy of these vaccines may be reduced.

## 6.4 Source and Use of Study Drugs

The study drugs in this study include thalidomide, methotrexate, and prednisone.

**Thalidomide:**

Commercially available thalidomide tablets will be used in this study. Each site will use commercially available thalidomide tablets for treatment according to clinical needs. Refer to local prescribing information for dosage form, packaging, and storage of thalidomide.

**Methotrexate:**

Commercially available methotrexate tablets will be used in this study. Each site will use commercially available methotrexate tablets according to clinical needs. Refer to local prescribing information for dosage form, packaging, and storage of methotrexate.

**Prednisone:**

Commercially available prednisone tablets will be used in this study. Each site will use commercially available prednisone according to clinical needs. Refer to local prescribing information for dosage form, packaging, and storage of prednisone.

# 7 Adverse Reactions and Dose Adjustment

## 7.1 Common Adverse Reactions of Thalidomide

**Teratogenic Effects:**

Thalidomide should not be used in pregnant women. When administered to pregnant women, thalidomide may cause fetal harm. Thalidomide is a highly potent human teratogen that induces severe and life-threatening birth defects at high frequencies, even with single dose. Approximately 40% of infants whose mothers have received this drug are reported to have died at birth or shortly after birth. Pregnant patients will not be enrolled in the study.

**Constipation:**

Administration of thalidomide may irritate the gastrointestinal tract, thus resulting in constipation.

**Nausea:**

Nausea ranks as the second most common adverse reaction in the digestive system, most nausea is mild to moderate in severity.

**Venous and Arterial Thromboembolism:**

Thalidomide may increase the risk of venous thromboembolism (eg, deep vein thrombosis and pulmonary embolism), which may be significantly increased when thalidomide is used in combination with standard chemotherapeutic agents including dexamethasone.

**Peripheral Neuropathy:**

Thalidomide may cause permanent nerve damage. Peripheral neuropathy is common (≥10%) and potentially irreversible with the serious adverse reaction to the treatment with thalidomide. Peripheral neuropathy usually develops after prolonged use over several months. However, peripheral neuropathy after relatively short-term use has also been reported. The correlation with cumulative dose is unknown. Symptoms may appear for some time after the discontinuation of treatment with thalidomide, and these symptoms may disappear slowly or completely.

**Neutropenia:**

White blood cell (WBC) count decreased with the clinical use of thalidomide, including neutropenia, have been reported.

**Thrombocytopenia:**

Grade 3 or 4 thrombocytopenia with the clinical use of thalidomide has been reported.

**Severe Skin Reactions**

Severe skin reactions have been reported for the treatment with thalidomide, including Stevens-Johnson syndrome (SJS), toxic epidermal necrolysis (TEN), and drug reaction

with eosinophilia and systemic symptoms (DRESS). DRESS may result in skin reactions (eg, rash or exfoliative dermatitis), eosinophilia, fever, and/or lymphadenopathy with systemic complications, such as hepatitis, nephritis, pneumonia, myocarditis, and/or pericarditis.

**Allergy**

Hypersensitivity reactions, including angioedema and allergic reactions to thalidomide, have been reported. Signs and symptoms include macular rash, which may be associated with fever, tachycardia, and hypotension.

**Seizure**

Although no premarketing controlled clinical trials have been reported, seizures, including severe convulsions, have been reported during the approval of thalidomide in clinical practice.

**Tumor Lysis Syndrome**

Monitor patients at risk for TLS (eg, patients with high tumor load before treatment)

**HIV Viral Load Increased**

In a randomized, placebo-controlled trial of thalidomide in HIV seropositive patients, increased plasma HIV RNA levels were observed with the median change from placebo of 0.42 log10 copies of HIV RNA/mL ($p = 0.04$). A similar trend was observed in another unpublished study of HIV seropositive patients. The clinical significance of this increase is unknown. Both studies were conducted before the implementation of highly active antiretroviral therapy.

**Bradycardia**

Bradycardia has been reported to be associated with the use of thalidomide. There are reported cases of bradycardia, some requiring medical intervention. The clinical significance and underlying etiology of bradycardia found in some patients treated with thalidomide are unknown.

**Somnolence**

Thalidomide often causes somnolence. Patients should be advised to avoid somnolence and not to take other medications that may cause somnolence without medical advice.

## 7.2 Common Adverse Reactions of Methotrexate

Methotrexate, an organic compound, is mainly used as an antifolate antineoplastic drug, which can inhibit dihydrofolate reductase, thereby impeding synthesis in tumor cells and inhibiting the growth and proliferation. Refer to the locally approved labeling (eg, refer to local prescribing information) for details of methotrexate. In this study, low-dose methotrexate is administered orally at 10 mg/m$^2$, once a week. The most common adverse reactions of low-dose methotrexate are summarized below.

**Elevated Transaminase**

Elevated transaminase is the most common adverse reaction of methotrexate and also the most common non-hematological AE, which is usually mild in severity. Liver function tests should be performed regularly after administration. Although these mild AEs will not lead to the discontinuation of methotrexate, this drug should be used with caution.

**Cytopenia**

In patients receiving low-dose methotrexate, cytopenia (neutropenia, thrombocytopenia, and anemia) is generally reported as Grade 1 or 2, but treatment-emergent Grade 3 or 4 cytopenia (neutropenia, thrombocytopenia, and anemia) is also reported. Anemia is the most common hematological adverse reaction, followed by neutropenia and thrombocytopenia.

**Nausea and vomiting**

Nausea and vomiting are another common non-hematological AEs. Other frequently reported gastrointestinal events included diarrhea, abdominal pain, cholecystitis, and gallstones. These events are rarely serious. If symptoms are severe or persist for a long time, follow the guidelines for dose adjustment in the protocol.

**Stomatitis**

Stomatitis has an incidence of < 10% and is generally of Grade 1 or 2. Symptoms may improve after symptomatic treatment.

**Rash**

Rash can be manifested as erythematous rash, alopecia, pruritus, redness and swelling, with an incidence of <3%. There are rare case reports of severe skin reactions, including toxic epidermal necrolysis, Stevens-Johnson syndrome, exfoliative dermatitis, cutaneous necrosis, and erythema multiforme.

## 7.3 Common Adverse Reactions of Prednisone

Prednisone is a glucocorticoid with anti-inflammatory, anti-allergic, antirheumatic and immunosuppressive effects. It is a commonly used medication for hematologic disorders. Due to its frequent clinical use, details will not be provided in this protocol.

**Water-electrolyte Disturbance**

Water-electrolyte disturbance includes sodium retention, fluid retention, congestive heart failure in susceptible patients, potassium loss, hypokalemic alkalosis, and hypertension.

**Musculoskeletal System**

Musculoskeletal system events include myasthenia, steroid myopathy, muscle tissue loss, osteoporosis, tendon rupture (especially Achilles's tendon rupture), vertebral compression fracture, aseptic necrosis of femoral and humeral heads, and pathological fracture of diseased long bone.

**Digestive System**

Digestive system events include ulcer (possible perforation and bleeding), pancreatitis, abdominal distension, and ulcerative esophagitis.

**Skin/Subcutaneous Tissue**

Difficult-to-heal wounds, thin fragile skin, petechiae and ecchymosis, facial erythema, and increased sweating may suppress reactions to skin tests.

**Metabolic System**

Metabolic system event includes negative nitrogen balance caused by protein catabolism.

**Nervous System**

Elevated intracranial pressure, vertigo and headache with papilledema (pseudoencephaloma) usually occur after treatment.

**Endocrine System**

Endocrine system events include irregular menstruation, development of Cushingoid syndrome, secondary adrenocortical and pituitary unresponsiveness (especially in stressful states such as trauma, surgery or disease), inhibition of growth and development in children, reduced carbohydrate tolerance, manifestations of latent diabetes, increased demand for insulin or oral hypoglycemic agents in diabetic patients.

**Eyes**

Eyes events include posterior subcapsular cataract, elevated intraocular pressure, glaucoma, and proptosis (exophthalmos).

**Anaphylaxis**

Anaphylaxis events include urticaria and other allergy, anaphylaxis or hypersensitivity.

## 7.4 Guidelines for Dose Adjustment and Discontinuation

In the event of toxicity, the investigator should accurately assess the severity of AEs and serious adverse events (SAEs) based on the patient's condition and the National Cancer Institute (NCI) Common Terminology Criteria for Adverse Events (CTCAE, Version 5.0). Appropriate medication (including infusions, cytokine growth factors, antiemetics, antidiarrheals, etc.) should be administered to treatment AEs. All AEs will be assessed from the time of the first dose of the study treatment until the final study visit. Patients who have experienced AEs may be followed up after the EOS visit for additional assessments until the AE resolves or is deemed stable.

For patients who are unable to tolerate the protocol-specified administration regimen, treatment discontinuation or dose adjustments are allowed. Before treatment discontinuation and dose adjustment, the attribution of AEs to the 3 drugs (thalidomide, methotrexate, and prednisone) should be determined. For non-hematological toxicities attributable to methotrexate and prednisone (not thalidomide), administration of thalidomide may be continued.

### 7.4.1 Dose Adjustment and Discontinuation of Thalidomide

If thalidomide-related peripheral neuropathy occurs, neuroprotective drugs can be used as appropriate; if there are ≥ Grade 2 or Grade 1 side effects with pain, reduce the dose by 1 dose level and discontinue in those without improvement. See Appendix 4 for details of thalidomide neurotoxicity grading and dose adjustment.

### 7.4.2 Dose Adjustment and Discontinuation of Methotrexate

Methotrexate is contraindicated in LGLL patients with an estimated glomerular filtration rate (eGFR) <30 mL/min. For patients with an eGFR of 30 to 59 mL/min, a lower initial dosage is required; and a more gradual dose increase, close monitoring, and a lower maximum weekly dose should be considered, depending on the overall clinical conditions. Although low doses of methotrexate are not nephrotoxic, methotrexate is almost completely excreted through the kidney. Therefore, kidney function should be monitored. If renal insufficiency occurs, the methotrexate dose should be reduced or discontinued.

Guidelines from Cancer Care Ontario in Canada recommend a 25% reduction of dose at CrCl of 80 mL/min, 40% reduction at CrCl of 60 mL/min, 50% reduction at CrCl of 50 mL/min, and discontinuation at CrCl <50 mL/min. The guidelines also state that less conservative dose adjustments may be considered when using a low-dose regimen (<50 $mg/m^2$). High-dose methotrexate could only be administered only when CrCl is >60 mL/min.

See Appendix 2 for the calculation method of CrCl.

### 7.4.3 Dose Adjustment and Discontinuation of Prednisone

The initial dose of prednisone is 0.5 mg/kg QOD, which should be adjusted according to the hematologic index. Actively monitor blood pressure and blood glucose, and observe adverse reactions of glucocorticoids. If there are adverse reactions of Grade 1 or 2, the dose can be reduced. If adverse reactions with ≥ Grade 3 occur, dose can be gradually reduced and eventually discontinued. If steroid-induced acute psychotic symptoms occur and antipsychotics are ineffective; or if corneal ulcers caused by the herpes virus occur, which can quickly lead to corneal perforation and may result in permanent blindness, discontinue the use of prednisone immediately or rapidly reduce the dosage significantly, instead of reducing it gradually.

## 7.5 Other Treatment Considerations

During the patient's treatment, if unrelated illnesses or accidents occur, and there is a need to temporarily stop the treatment, it should be decided in consultation with the investigator.

# 8. Study Procedures

Only untreated or standard-of-care naive patients, patients with poor responses (failure to achieve PR) or relapse after receiving a non-methotrexate/thalidomide-based regimen, and patients who did not achieve PR within 4 weeks of methotrexate monotherapy can be enrolled in this study. Therefore, prior treatment and efficacy will also be evaluated before enrollment. The study consists of a screening period (Day −28 to Day 0), a treatment period, end of treatment, and a follow-up period.

## 8.1 Screening Period

All patients will undergo study eligibility screening, which will be completed within 4 weeks before the start of the study, including:

- Review of inclusion/exclusion criteria
- Physical examinations
- Vital signs measurements (body temperature, pulse, systolic and diastolic blood pressure, and respiratory rate)
- Height, weight, and body surface area
- ECOG performance status
- Disease-related signs and symptoms
- Assessment and recording of serious pre-treatment events
- Complete medical history (previous treatment and efficacy, enlargement of liver, spleen and lymph nodes; presence of other comorbidities)
- Hematology: Hemoglobin, ANC, WBC count, platelets
- Blood chemistry: Serum glucose, AST, ALT, total bilirubin, creatinine, Na, K, uric acid, total protein, and albumin
- Serum lactate dehydrogenase (LDH)
- Serum β2 microglobulin
- Serum immunoglobulin quantification
- Serological tests for HIV, hepatitis C virus (HCV), and HBV (including hepatitis B surface antigen [HBsAg], anti- hepatitis B surface antibody [HBsAb], and anti-hepatitis B core antibody [HBcAb])
- HBV-DNA and HCV RNA: HBV-DNA and HCV RNA will be used in patients with serologically positive HBV or HCV, respectively
- Coagulation function (8 items)
- Detection of peripheral blood lymphocyte subsets (B/T/NK; Treg; T-cell immune function; TH1/TH2)

- Peripheral blood flow cytometry
- Pregnancy test (if applicable)
- Urinalysis and stool
- Bone marrow aspiration and biopsy
- Bone marrow smear, immunohistochemistry, and flow immunophenotyping
- Flow cytometry detection of LGLs in patient's bone marrow fluid or peripheral blood; flow cytometry detection of killer immunoglobulin-like receptor; TCR rearrangement; TCRvβ flow cytometry, karyotyping; next generation sequencing
- Electrocardiograph (ECG)
- Computed tomography (CT) for assessment of measurable lesions (including neck, chest, abdomen, and pelvis)
- Assessment of cardiac function and ejection fraction by two-dimensional echocardiography; abdominal color Doppler ultrasound, etc.

Other test options:

Infection-related markers; fluorescence in situ immunohybridization (17p13); complete test of viruses, hemolysis-related tests (if relevant clinical manifestations are present): Coombs test and cold agglutinin test; rheumatoid factor, complement, ENA antibody spectrum, antinuclear antibody, antiplatelet antibody (when immune thrombocytopenia is suspected), antiphospholipid antibody (when combined with thrombus), serum ferritin plus serum iron test.

## 8.2 Treatment Period

Collect, record and report patient information and data in a timely manner to ensure that the data and information are timely, consistent, complete, reliable, and accurate. AEs, especially SAEs, should be treated in a timely and proper manner, timely tracked, followed up, recorded, and reported. Original records should be kept.

**Months 1 to 24 of TPM Treatment**

- Physical examination and vital sign measurements
- ECOG performance status
- Assessment and recording of AEs to further assess whether the treatment can be continued
- Detection of LGLs in peripheral blood
- Hematology: Hemoglobin, ANC, WBC count, platelets.
- Blood chemistry: Serum glucose, AST, ALT, total bilirubin, creatinine, uric acid, etc.

**At the End of Cycle 1 (Every 3 months as 1 cycle)**

- Physical examination and basic vital sign measurements
- ECOG performance status
- Assessment and recording of AEs to further assess whether the treatment can be continued
- Hematology: Hemoglobin, ANC, WBC count, platelets
- Blood chemistry: Serum glucose, AST, ALT, total bilirubin, creatinine, uric acid, LDH, etc.
- Detection of LGLs in peripheral blood
- Detection of peripheral blood lymphocyte subsets (B/T/NK; Treg; T-cell immune function; TH1/TH2)
- Peripheral blood flow cytometry
- Peripheral blood TCRvβ flow cytometry
- Bone marrow smear, bone marrow biopsy, and immunohistochemistry
- Chromosome karyotype examination in bone marrow or peripheral blood
- ECG
- Evaluation of measurable lesions (including neck, chest, abdomen, and pelvis) by CT in patients with lymph node enlargement before admission
- Assessment of cardiac function and ejection fraction by two-dimensional echocardiography; abdominal color Doppler ultrasound, etc.
- Coagulation function

**At the End of Cycles 2 to 8 (Every 3 months as 1 cycle)**

- Physical examination and vital sign measurements
- ECOG performance status
- Assessment and recording of AEs to further assess whether the treatment can be continued
- Hematology: Hemoglobin, ANC, WBC count, platelets
- Blood chemistry: Serum glucose, AST, ALT, total bilirubin, creatinine, uric acid, LDH, etc.
- Detection of LGLs in peripheral blood
- Detection of peripheral blood lymphocyte subsets (B/T/NK; Treg; T-cell immune function; TH1/TH2)
- Peripheral blood flow cytometry
- Peripheral blood TCRvβ flow cytometry

- ECG
- Evaluation of measurable lesions (including neck, chest, abdomen, and pelvis) by CT in patients with lymph node enlargement before admission
- Assessment of cardiac function and ejection fraction by two-dimensional echocardiography; abdominal color Doppler ultrasound, etc.
- Coagulation function

### 8.2.1 Disease Progression

The specific tests are the same as those in the screening period.

## 8.3 List of Tests and Key Time Points

| Test | Screening/ Recurrence Progression | Post-Cycle 1 | Every 3 Months Thereafter |
|---|---|---|---|
| Hematology | √ | √ | √ |
| Blood type | √ | | |
| HIV/syphilis/hepatitis | √ | | |
| Complete test of viruses | √ | | |
| Lymphocyte subsets (at Blood Research Institute) | √ | √ | √ |
| All items of cytokines (at Blood Research Institute is available) | √ | √ | √ |
| Detection of peripheral blood large granular lymphocytes | √ | √ | √ |
| TCRvβ flow cytometry | √ | √ | √ |
| Immunoglobulin quantification | √ | | |
| Hepatorenal and cardiac function, blood glucose | √ | √ | √ |
| Electrolytes | √ | √ | √ |
| Lactate dehydrogenase | √ | | |
| Blood β2-MG | √ | | |
| Coagulation (8 items) | √ | √ | √ |
| Classification of bone marrow smears | √ | When necessary | When necessary |
| Bone marrow biopsy (paraffin-embedded) | √ | When necessary | When necessary |
| Flow immunophenotyping | √ | When necessary | When necessary |
| Karyotype | √ | When necessary | When necessary |
| TCR β、TCRγ、TCRδ | √ | When necessary | When necessary |
| FISH | When necessary | When necessary | When necessary |
| Second-generation genetic testing | √ | | |
| Electrocardiograph | √ | √ | √ |
| Cardiac color Doppler ultrasound (cardiac function) | √ | √ | √ |
| B-scan ultrasonography (superficial lymph nodes) | √ | √ | √ |
| Cervical/chest/abdominal computed tomography | √ | When necessary | When necessary |
| Test of infection foci | When necessary | When necessary | When necessary |

## 8.4 Early Withdrawal (Discontinuation of Study Treatment)

**Three months after the Last Treatment:**

- Physical examinations
- Vital signs measurements (body temperature, pulse, systolic and diastolic blood pressure, and respiratory rate)
- ECOG performance status
- Disease-related signs and symptoms
- Assessment and recording of AEs
- Hematology: Hemoglobin, ANC, WBC count, platelets
- Blood chemistry: Serum glucose, AST, ALT, total bilirubin, creatinine, Na, K, uric acid, total protein, and albumin
- Serum LDH
- Serum immunoglobulin quantification
- Urinalysis and stool
- Detection of LGLs in peripheral blood
- Detection of peripheral blood lymphocyte subsets (B/T/NK; Treg; T-cell immune function; TH1/TH2)
- Peripheral blood flow cytometry
- Peripheral blood TCRvβ flow cytometry
- Bone marrow smear, bone marrow biopsy, and immunohistochemistry
- Chromosome karyotype examination in bone marrow or peripheral blood
- ECG
- Evaluation of measurable lesions (including neck, chest, abdomen, and pelvis) by CT in patients with lymph node enlargement before admission
- Assessment of cardiac function and ejection fraction by two-dimensional echocardiography; abdominal color Doppler ultrasound, etc.
- Coagulation function

Following this assessment, patients will be followed up twice a year until the EOS, including survival, disease status, and long-term toxicity.

## 8.5 Follow-up

### 8.5.1 Survival Follow-up

Survival follow-up will be performed after the end-of-treatment visit (including patients who discontinue treatment early due to intolerance). Patients will be re-examined every

6 months and followed up for 2 years from the end of treatment.

### 8.5.2 Safety Follow-up

Safety follow-up will be performed for all patients who complete or discontinue treatment until 1 year after the last dose of the study drug, or until initiation of a new antineoplastic therapy (whichever occurs first).

Patients will be contacted by phone 1 year after the last dose, or asked to the study site for a safety visit to collect the ongoing AEs or changes in laboratory abnormalities, and to assess new AEs, SAEs, new concomitant medications, or changes in current medications since the last assessment. Any new conditions that occur during the safety follow-up need to be documented in the CRF as appropriate. After the safety follow-up (ie, 28 days after the last dose), there is no need to actively collect AEs. However, if an SAE occurs and the investigator assesses a reasonable causality to the study drug, the SAE should be reported.

AEs (regardless of causality) that do not completely resolve at the end of safety follow-up must be followed up until recovery (chronic, baseline value, or completely recovery), or clinical stability is achieved.

# 9. Efficacy Evaluation

## 9.1 Definition

### 9.1.1 General Definition

Evaluable toxicity: All patients who have received at least 1 month of TPM treatment are evaluable for toxicity evaluation from the first treatment.

Evaluable objective response: Only patients who have a measurable disease at baseline, have received at least 1 treatment cycle, and have been re-evaluated for the disease will be considered as evaluable responses. These patients will be categorized for their responses according to the following definitions (Note: Patients who demonstrate objective PD or death by the end of Cycle 1 will also be considered evaluable).

## 9.2 Efficacy Evaluation

The evaluation will be started from Month 3. Hematologic ORR is defined as the sum of CR and PR.

Hematologic CR: Clinical symptoms and signs turn normal (splenomegaly disappears), and blood cell count completed turn normal, ie, hemoglobin >110 g/L, platelets >100 × $10^9$/L, ANC >1.5 × $10^9$/L, lymphocyte count <4 × $10^9$/L, and LGLs in peripheral blood are within the normal range (<0.5 × $10^9$/L).

Complete molecular response: No LGLs can be detected when reaching CR (negative TCR rearrangement by flow cytometry or polymerase chain reaction [PCR]).

Hematologic PR: Hematologic PR is defined as blood cell count that do not meet criteria for CR but improvement is observed, such as neutrophil counts >0.5 × $10^9$/L without repeated infection, an increase of hemoglobin level from baseline by more than 20 g/L without blood transfusion, platelets >50 × $10^9$/L.

SD: CR/PR criteria are not met after standardized treatment for 4 months, but no significant progression is observed.

PD: Cytopenia (hemoglobin decreased by 2 g/dL, <10 g/dL; ANC decreased by 0.5 × $10^9$/L, <1.0 × $10^9$/L; platelets decreased by 20 × $10^9$/L, <100 × $10^9$/L, requiring blood transfusion) or progressive worsening of organomegaly.

PFS: PFS is defined as the time from the first day of study to PD or death, including PD and death from any cause (eg, PD, toxicity, unrecorded progression in a new treatment, or death).

DoR: DoR is defined as the time when CR or PR is met until the first date of recurrent or progressive disease.

## 9.3 Study Endpoints Definition and Evaluation

**Primary Endpoint:**

- CRR of patients with symptomatic LGLL treated with thalidomide, prednisone, and methotrexate.

**Secondary Endpoints**

**1. Secondary Safety Endpoint**

- Incidence and severity of hematological and non-hematological AEs

Safety endpoints include SAEs, treatment-emergent adverse events (TEAEs), physical examination (including ECOG performance status), vital sign measurements, standard clinical laboratory parameters, and ECG. TEAEs will be graded according to NCI CTCAE Version 5.0.

**2. Secondary Efficacy Endpoints**

- Response rates: ORR and PR rate (time frame: 24 months after the last treatment)

Point estimates of rates will be calculated for each protocol analysis set. An estimate of the 95% CI for the response rate will also be derived. Graphical and descriptive analyses will be used to explore associations between relevant markers and responses.

- PFS and OS (time frame: 24 months after the last treatment)

Time from the first day to PD or death from any reason. The median duration of the overall response will be assessed with a 95% CI.

- DoR (time frame: 24 months after the last treatment)

The time when CR or PR is met until the first date of recurrent or progressive disease. The median duration of the overall response will be assessed with a 95% CI.

- Effect of biomarkers on efficacy and adverse reactions

**Exploratory Endpoint:**

- To explore biomarkers significantly and potentially associated with clinical efficacy

# 10. Safety Evaluation

Timely, accurate, and complete reporting and analysis of safety information from clinical studies are essential to protect patients and investigators and are mandatory by regulatory authorities worldwide. Each AE verbatim term will be coded to a system organ class and preferred term using the Medical Dictionary for Regulatory Activities (MedDRA).

## 10.1 Definition and Classification of Adverse Events

**Adverse Events:**

An AE is any untoward medical occurrence in a patient to whom a medicinal product is administered, and which does not necessarily have a causal relationship with this treatment. An AE can therefore be any unfavorable and unintended sign (eg, an abnormal laboratory finding), symptom, or disease temporally associated with the use of a medicinal product, whether considered related to this medicinal product. (As defined by the International Council for Harmonisation of Technical Requirements for Pharmaceuticals for Human Use [ICH]).

It includes any new event, any event worsened from baseline in severity or frequency, or abnormal results of diagnostic procedures, including laboratory test abnormalities.

**Serious Adverse Events:**

An SAE, as defined by ICH, as an AE that fulfils one or more of the following criteria at any dose:

- Results in death
- Is life-threatening ((life-threatening refers to a reaction in which the patient was at risk of death at the time of the reaction; it does not refer to a reaction that hypothetically might have caused death if more severe).)
- Requires hospitalization or prolongation of existing hospitalization
- Results in persistent disability/incapacity (The term disability means a substantial disruption of a person’s ability to conduct normal life functions.)
- Is a congenital anomaly/birth defect
- Is an important medical event

Note: Medical or scientific judgment should be exercised in deciding whether SAE reporting is appropriate in other situations such as important medical events that may not be immediately life-threatening or result in death or hospitalization but may jeopardize the patient or may require medical or surgical intervention to prevent one of the other outcomes listed in the above definition. These events should usually be considered serious.

Events that are not considered SAEs include planned hospitalization before enrollment

into the clinical study; a disease for elective treatment and unrelated to the indication under study or its treatment; events occurring in an emergency outpatient department and not leading to hospitalization (unless other criteria above are met); part of normal treatment or monitoring of the study indication and unrelated to condition worsening.

If an AE is considered serious, the AE page in the CRF and the SAE Report Form must be completed. The investigator will provide information on severity, start and end dates, relationship to the study drug, actions taken with the study drug, and outcomes.

**Suspected Unexpected Serious Adverse Reaction:**

For unexpected AEs, their nature or severity is inconsistent with the that of the study drug. For the study drug, the expectedness of an AE will depend on whether it is listed in the Investigator's Brochure of the study drug. For a drug with marketing authorization, the expectedness of an AE will depend on whether it is listed in the summary of product characteristics (SmPC).

**Severity Assessment:**

For AEs and SAEs, the investigator must assess the severity of the events. According to the NCI CTCAE Version 4.03, the severity of AEs will be graded from 1 to 5. A copy of the NCI CTCAE Version 5.0 can be downloaded from the homepage of the Cancer Therapy Evaluation Program (CTEP) (https://ctep.cancer.gov/protocolDevelopment/electronic_applications/docs/CTCAE_v5_Quick_Reference_5x7.pdf). In those cases where NCI CTCAE is not applicable, the severity should be defined according to the following criteria:

Grade Definition:

Grade 1 (mild AE): asymptomatic or mild symptoms; usually transient, not requiring treatment, and generally not interfering with daily activities

Grade 2 (moderate AE): Moderate discomfort, interfering with daily activities; usually relieved by basic treatment

Grade 3 (severe AE): Severe incapacitation, inability to perform daily activities, or significantly affecting clinical status, requiring intervention. Hospitalization may or may not be required.

Grade 4 (life-threatening or disabling AE): Life-threatening consequences, life-threatening; requiring hospitalization and clinical intervention.

Grade 5 (AE-related death): Death related to AE

**Causality Assessment:**

The investigator must determine that the relationship between the administration of the study drug and AEs/SAEs as defined below:

- Unrelated. AEs are not related to the use of the study drug.

- Unlikely/suspicious. AEs that are more likely to have alternative explanations, such as concomitant medications, concomitant diseases, or temporal relationships suggest little causality.
- Possibly. Treatment with the study drug caused or contributed to the AE. Alternative explanations, such as concomitant medications and diseases, are uncertain. The event follows a reasonable temporal sequence from the time of drug administration; therefore, causality cannot be ruled out.
- Probably. AEs are probably caused by the use of the study drug. A reasonable temporal sequence of the event with drug administration exists Alternative explanations don’t make sense, such as concomitant medications and concomitant diseases.
- Definitely. AEs that are listed as possible AE reactions and cannot be reasonably explained by alternative explanations, such as concomitant medications and concomitant diseases. The relationship in time is very suggestive (eg, it is challenged and re-challenged).
- Non-evaluable: The evidence for making a clinical judgment of causality is insufficient or incomplete.

**Treatment-related Mortality:**

If an AE related to the study drug is considered to result in the death, that event will be listed as “treatment-related mortality”.

## 10.2 Safety Evaluation

If a patient completes at least 1 month of effective treatment, the patient will be analyzed for safety. The tolerability of treatment will be assessed by evaluating laboratory parameters and AEs. The incidence of all AEs during the treatment and up to 30 days after the last drug administration will be recorded.

AEs will be classified by severity, duration, and frequency. Details of AEs will be listed by patients, including time of onset, duration, toxicity grade, corrective treatment, outcome, and relationship to the investigational product. Safety parameters will be assessed by 2 independent investigators who will assess severity and treatment each time.

Study drug should be discontinued for any uncontrolled non-hematological toxicity that may be associated with the study drug and of Grade 3 or higher, and for any hematological toxicity that meets the criteria. For patients requiring full-dose anticoagulation (eg, heparin), the study drug should be interrupted until the anticoagulation is stable. For patients requiring an invasive procedure or surgery, the study drug must be interrupted. Any other clinically important events, in which the investigator may deem appropriate dose delays, must be discussed with the CRA. Details of drug adjustment and discontinuation criteria, refer to Section 7.4.

## 10.3 Recording of Adverse Events

### Diagnosis/Pathogenic Events and Signs and Symptoms

All AEs and SAEs should be reported. If known, the diagnosis should be recorded on the CRF rather than individual sign and symptom. However, if a series of signs and/or symptoms cannot be medically characterized as a single syndrome at the time of reporting, individual sign and/or symptom should be recorded on the CRF as AEs or SAEs. If a serious of signs and/or symptoms can be medically characterized as a single syndrome (eg, nausea and vomiting), the most medically significant sign and/or symptom should be reported as an AE, and other signs and/or symptoms should be recorded in the Additional Case Details of the CRF. If a diagnosis is subsequently confirmed, the event term should be updated to reflect the medical diagnosis.

In general, AEs secondary to other events (eg, cascade events or clinical sequelae) should be identified by the primary cause (pathogenic event) and additional sequelae should be recorded in the Additional Case Details of the CRF.

However, AEs that are temporally separate or secondary to an initial event of medical significance should be recorded on the CRF as independent events.

### Persistent or Recurrent Adverse Events

A persistent AE is an ongoing extension with no resolution between assessment time points. Such events should be recorded only once on the CRF unless the severity increases. If persistent AEs become more severe, they should be recorded again on the AE page of the CRF.

Recurrent AEs are those that occur and resolve between assessment time points and subsequently recur. All recurrent AEs should be recorded on the AE page of the CRF.

### Laboratory Abnormalities

Only clinically significant laboratory abnormalities requiring active management are recorded as AE or SAE on the CRF. The criteria for clinical significance are as follows:

- Laboratory abnormalities with clinical symptoms
- Laboratory abnormalities requiring dose adjustment, interruption, or termination of the study drug
- Laboratory abnormalities requiring more frequent follow-up assessments and further diagnostic investigations, etc.
- Laboratory abnormalities requiring changes in concomitant medication, therapy, or treatment

If a clinically significant laboratory abnormality is a symptom of disease or syndrome (eg, alkaline phosphatase and bilirubin are 5 × ULN associated with cholecystitis), only the diagnosis (eg, cholecystitis) needs to be recorded on the AE page of the CRF.

If a clinically significant laboratory abnormality is not a symptom of disease or syndrome, the abnormality itself should be recorded on the CRF as an AE or SAE. Observations of the same clinically significant laboratory abnormalities at each visit should not be repeatedly recorded as AEs or SAEs on the CRF unless the severity or etiology changes.

**Number of Deaths**

Deaths that occur during the protocol-specified AE reporting period and are attributed solely to progression by the investigator will be recorded only on the CRF. Other deaths, regardless of attribution, will be recorded on the CRF and promptly reported to the investigator.

## 10.4 Reporting of Adverse Events

All AEs and SAEs that occur between the first study-related procedures and within 30 days after the last dose of the study drug will be reported. All AEs, regardless of severity or presumed relationship to the study drug, must be recorded on the CRF using medical terms. Refer to (https://ctep.cancer.gov/protocolDevelopment/electronic_applications/docs/CTCAE_v5_Quick_Reference_5x7.pdf) for details. The investigators also need to record their opinions on the relationship between AEs and the study drug on the CRF. All actions required for AE management must be recorded in the source documents. All SAE reports must be submitted within 24 hours. For initial SAEs, all case details should be collected within 24 hours. The initial report must be as complete as possible, including details of the current diseases and SAEs, as well as an assessment of the causality between the event and the study drug. Information that is not available at the time of initial reporting (eg, end date or laboratory values of AEs) must be recorded on subsequent SAE forms when available and/or immediately upon request. The investigator must keep a copy of all SAE information on file. All SAEs that are unresolved at the time a patient discontinues study must be followed until the event is fully resolved, stabilized/sequelae resolved, or baseline (if any) is returned.

## 10.5 Data Safety

Each site shall complete the CRF and adverse reaction form or provide pictures according to the requirements of the CRF, and submit to the leading site (Institute of Hematology and Blood Diseases Hospital, Chinese Academy of Medical Sciences) for data entry and management after being reviewed by investigators. All process should be recorded.

Two independent site personnel should be responsible for data entry and management at each site. To ensure the accuracy of data, two statisticians independently enter them in duplicate and check. For queries about the CRF, the data statistician will fill in the Data Request Order (DRO) and send it to the investigator, who should promptly respond and return the DRO. Based on the investigator’s response, the data statistician

will make necessary data modifications, confirmations, and entries, and send a DRO again if necessary.

A Data Safety Monitoring Committee (DSMC) consisting of at least 4 independent members (2 clinicians and 2 independent statisticians) will be established for this study. The DSMC will meet regularly to review safety and efficacy data from trials prepared by independent statisticians, and the clinician will perform interim analyses. All data presented at the meeting will be kept confidential. After each meeting, the DSMC will prepare a report and may recommend changes to the protocol.

# 11. Statistical Analysis

## 11.1 General Consideration

The statistical analyses of this study will be conducted using the statistical analysis software SAS 9.4.

Quantitative data will be statistically described using mean, standard deviation, median, maximum, and minimum. Enumeration data or grade data are expressed in terms of the number and percentage of patients.

1. Describe the completion of the trial, and statistically describe the excluded and dropout patients one by one.
2. Describe demographics and other baseline parameters.

Efficacy analysis: Summarize the data with descriptive statistics, and the main indicator is the response rate of patients, including ORR, CRR, and PR rate. The $\chi^2$ test is used for the overall comparison, while the $\chi^2$ partitioning method is used to correct α for pairwise inter-group comparisons. Continuous variables will be presented as number, mean, standard deviation, median, and range. Discrete variables will be summarized as frequency. For time-to-event variables such as DoR to treatment, the Kaplan-Meier method is used to estimate the event occurrence rate and plot the curve. The comparison of the curves is carried out using the Log-rank method. The Cox regression model is used to analyze the influencing factors of event occurrence.

Comparisons between the 2 treatment groups are as follows: for changes of continuous variables from baseline to a specific post-baseline time point, an analysis of variance (ANOVA) is used. For discrete variables, the Cochran-Mantel-Haenszel chi-square test is used. For variables of time to onset, a stratified log-rank test is used. Except for the analysis of primary indicators, the two-sided α level in other tests is 0.05. If pairwise comparisons between groups are involved.

## 11.2 Safety Analysis

The safety profile will be analyzed based on AEs, physical examination, vital sign measurements, laboratory measurements, and ECG findings. AEs will be graded as per NCI CTCAE (Version 5.0).

In general, the safety analysis will be descriptive and presented in tabular form with appropriate summary statistics. The number of patients and events of hematological and non-hematological toxicity (based on NCI CTCAE Version 5.0) will be listed, and the incidence will be calculated.

## 11.3 Efficacy Analysis

The efficacy analysis will involve CRR and ORR (sum of CR and PR rates). Efficacy variables will be tabulated and summarized. For the numbers and percentages of CRR

and ORR, point estimates and 95% exact binomial CIs will be provided.

Time-to-event variables including DoR, PFS, and OS will be summarized descriptively using the Kaplan-Meier method. The rules for DoR, PFS, and OS analyses will be specified in the Statistical Analysis Plan (SAP). Growth modulation indices (intra-subject ratios of DOR, PFS, and OS, post-study treatment versus DOR, PFS, and OS post the most recent prior treatment regimen) will be summarized.

## 11.4 Biomarkers Exploratory Analysis

The exploratory analysis of biomarkers will be tabulated and summarized using descriptive statistics.

# 12. Quality Control and Assurance of Good Clinical Practice

## 12.1 Monitoring, Audit, and Inspection

Monitoring will be conducted mainly by email and phone during the study. The on-site CRA will visit the site when required, mainly in cases of data inconsistencies, to check the completeness of patient records, the accuracy of CRF entries, adherence to the protocol and good clinical practice (GCP) guidelines, and the status of enrollment. Key investigators must assist the on-site CRA during these visits.

The investigator must retain source documents for each patient in the study, including medical records and visit records (hospital or clinic medical records) containing demographic and medical information, laboratory data, ECG, and results of any other tests or assessments. All information on the CRF must be traceable to these source files in the patient file. The investigator must also retain the original signed ICF from patients (providing the patients with a signed copy).

The investigator must grant the CRA access to all relevant source documents to confirm the consistency with CRF entries. The safety monitoring standards require a thorough validation of the existence of informed consent, compliance with inclusion/exclusion criteria, documentation of SAEs, and data records to be used for all primary and safety variables. Additional checks for consistency of source data with the CRF are performed according to a study-specific monitoring plan. Information about the patient’s identity will not be disclosed in source files.

## 12.2 Investigator’s Responsibilities

The responsibilities of investigators are set out in the ICH GCP guidelines. The investigator must grant the CRA access to relevant records to confirm the above.

The investigator is responsible to record for all patients who have signed ICFs and have been screened for participation in the study. For patients who are excluded, reasons must be recorded in the patient’s source documents.

No procedures/assessments/measurements/tests, other than those outlined herein or on the study evaluation schedule, can be performed without prior written approval from the Principal Investigator, or unless deemed necessary for the patient’s medical care by the investigator. The investigator and/or authorized designee must enter study data into the provided CRFs. Data on the CRFs will be recorded anonymously to protect the identity of the patients by using unique identifiers that prevent the disclosure of personal identity information.

The investigator or designee must be available during the monitoring visit to review data and resolve any queries, as well as allow direct access to patient records (eg, medical records, office charts, hospital charts, and study-related charts) for source data validation. The CRFs must be completed as soon as possible after the patient's visit, but no later than the start of each monitoring visit, and provided to the representative for

checking accuracy and completeness.

# 13. Ethical and Regulatory Considerations

## 13.1 Review and Approval by Institutional Review Board/Independent Ethics Committee

This study will be conducted in accordance with the Declaration of Helsinki - Ethical Principles for Medical Research Involving Human Subjects (for more information, see: http://www.wma.net/e/policy/b3.html). The review by the Institutional Review Board/Independent Ethics Committee (IRB/IEC) of this study, as well as the conduct of the study and the methods used to obtain informed consent, must also comply with the principles outlined in the declaration and the ICH guidelines. The protocol, proposed ICF, and other patient information must be reviewed by IRB/IEC before the conduct of this study. A signed and dated statement indicating that the protocol and the ICF have been approved must be provided to the Institution before the start of the study.

The Institute of Hematology and Blood Diseases Hospital, Chinese Academy of Medical Sciences (Institute of Hematology, Chinese Academy of Medical Sciences) is the sponsor of the study. The Principal Investigator and the on-site investigator will prepare study-related documents (if applicable) and to submit to appropriate institutions and obtain written approval for this study. Approval will be obtained before the start of the study.

Participating sites will be provided with copies of the IRB/IEC's approved protocol and ICF. The approval of the protocol and ICF must specify the date of approval, protocol number, and version or revision number.

The participating sites are responsible for notifying the Principal Investigator, Safety Monitoring office, and IRB/IEC of any major deviations from the protocol, or any other condition that might pose additional risks to the patients. Any advertisements used to recruit patients must be reviewed and approved by the Principal Investigator and IRB/IEC before use.

## 13.2 Protocol Amendment

As the study progresses, any revisions to this protocol that appear appropriate will be submitted to the IRB/IEC for written approval before implementation of the revised version. The written approval signed by the IRB/IEC should specifically mention the investigator, protocol number, and title, as well as any applicable revision number. Administrative revisions do not require IRB/IEC approval, but will be submitted to the IRB/IEC for reference.

## 13.3 Informed Consent

In accordance with GCP, investigators must obtain informed consent from patients or their designees before any study-related procedures. The documentation of informed

consent and the informed consent obtaining process should be recorded in the patient's source documents before the patient enters the study. Patients will be informed that their participation is voluntary, and they can withdraw their consent at any time. Patients will be informed that if they don't participate in the study, it will not affect the treatment received, they can use alternative treatments, and such refusal will not affect future treatments. Patients or legally representatives will have sufficient time to read the ICF and will have the opportunity to ask questions. After the interpretation and before the entry to the study, the ICF should be signed by the patients or legally representatives. After consent is obtained, a copy of the ICF must be provided to the patient.

If the patient or a legally representative is unable to read or write, an impartial witness should be present throughout the informed consent process (including reading and interpreting all written information) and sign the ICF in person after the consent is obtained.

The original consent form, signed and dated by the patient and investigator, must be kept in the Investigator's Study File before the patient enters the study, and a copy should be provided to the patient. In addition, if the protocol is amended and affects the content of the ICF, the ICF must be updated. Patients participating in the study must re-consent to the revised ICF when a protocol amendment is implemented. The amended consent form, signed and dated by the patient and the investigator, must be kept in the Investigator's Study File, and a copy should be provided to the patient.

# 14. Data Handling and Documentation

## 14.1 Data/Document

The investigator must ensure that records and documents related to the conduct of the study and dispensing of the study drug, ie, copies of CRFs and source files, source documents, data and records (eg, medical records; clinical and office charts; laboratory notes; memorandum; listing of patient diaries or assessments; dispensing records of pharmacy; recorded data from automated instruments; a certified true copy or transcript; microfilm; photographic negatives, microfilm or magnetic media; X-ray; patient files) and records kept in the pharmacy, laboratory and medical technical departments involved in clinical studies are complete, accurate, and documented.

## 14.2 Data Management

Data will be entered into the clinical database. These data will be validated electronically using online checks during data entry, as well as through programmed editing checks as specified by the study team. If necessary, discrepancies in the data will be brought to the attention of the study team and site personnel in the form of a Data Clarification Form. Solutions to these queries will be reflected in the database. An audit trail within the system will track all changes made to data.

## 14.3 Record Retention

The investigator must maintain records of all study files and supporting information relevant to the conduct of the study. Documents include, but are not limited to, the protocol, CRFs, patient participation reports, AE reports, patient source data, correspondence with health authorities and IRB/IEC, ICFs, investigator curricula vitae, monitoring visit logs, laboratory reference ranges, laboratory accreditation or quality control procedures, and laboratory director curricula vitae. Patient files and other source data must be kept for the maximum period allowed by the hospital, institution, or private clinic. If the investigator wishes to assign study files to others, move them to another location, or is unable to retain them for a specified period, he/she must consult the CRA. The investigator must retain study records in accordance with local laws or requirements, whichever is longer. CRA will notify the investigator of the retention date. All study files should be available upon request. Essential documents should be retained until at least 2 years after the last approval of a marketing application in an ICH region and until there are no pending or contemplated marketing applications in an ICH region or at least 2 years have elapsed since the formal discontinuation of clinical development of the study treatment. These documents should be retained for a longer period. If required by other applicable regulatory requirements, these documents should be retained for a longer period of time.

# 15. Confidentiality

The collection and processing of personal data from patients enrolled in this study will be limited to those data that are necessary to investigate the efficacy, safety, quality, and effectiveness of the investigational product used in this study. Adequate precautions must be taken for the collection and processing of such data to ensure confidentiality and compliance with applicable laws and regulations on data privacy protection.

The Investigator (Sponsor) ensures that personal data will be:

1. processed fairly and legally
2. collected for specific, explicit, and legitimate purposes, and will not be further processed for purposes other than study
3. adequate, relevant and not excessive for the purposes set out above
4. accurate and keep up-to-date as necessary

Before data collection, consent for processing of personal data will be obtained from the patient (or the legally representative). Such consent should also include the transfer of data to other entities or countries. Patients have the right to request access to their personal data through an investigator and to request correction of any incorrect or incomplete data. Applicable steps should be taken to respond to such requests, taking into account the nature of the request, the conditions of the study, and applicable laws and regulations.

Enrolled patients will be registered on website before treatment is initiated. Patient names will not be recorded in the data center. A serial identification number will be automatically assigned to each patient enrolled in the trial. This number will be used for identification and must be included in all CRFs.

# 16. References


1. Swerdlow SH, Campo E, Pileri SA, Harris NL, Stein H, Siebert R, et al. The 2016 revision of the World Health Organization classification of lymphoid neoplasms. **Blood 2016**, 127(20): 2375-2390.
2. Loughran TJ, Kadin ME, Starkebaum G, Abkowitz JL, Clark EA, Disteche C, et al. Leukemia of large granular lymphocytes: association with clonal chromosomal abnormalities and autoimmune neutropenia, thrombocytopenia, and hemolytic anemia. **Ann Intern Med 1985**, 102(2): 169-175.
3. Dinmohamed AG, Brink M, Visser O, Jongen-Lavrencic M. Population-based analyses among 184 patients diagnosed with large granular lymphocyte leukemia in the Netherlands between 2001 and 2013. **Leukemia 2016**, 30(6): 1449-1451.
4. Boeckx N, Uyttebroeck A, Langerak AW, Brusselmans C, Goossens W, Bossuyt X. Clonal proliferation of T-Cell large granular lymphocytes. **Pediatr Blood Cancer 2004**, 42(3): 275-277.
5. Clemente MJ, Wlodarski MW, Makishima H, Viny AD, Bretschneider I, Shaik M, et al. Clonal drift demonstrates unexpected dynamics of the T-cell repertoire in T-large granular lymphocyte leukemia. **Blood 2011**, 118(16): 4384-4393.
6. Lamy T, Moignet A, Loughran TJ. LGL leukemia: from pathogenesis to treatment. **Blood 2017**, 129(9): 1082-1094.
7. Koskela HL, Eldfors S, Ellonen P, van Adrichem AJ, Kuusanmaki H, Andersson EI, et al. Somatic STAT3 mutations in large granular lymphocytic leukemia. **N Engl J Med 2012**, 366(20): 1905-1913.
8. Jerez A, Clemente MJ, Makishima H, Koskela H, Leblanc F, Peng NK, et al. STAT3 mutations unify the pathogenesis of chronic lymphoproliferative disorders of NK cells and T-cell large granular lymphocyte leukemia. **Blood 2012**, 120(15): 3048-3057.
9. Sanikommu SR, Clemente MJ, Chomczynski P, Afable MN, Jerez A, Thota S, et al. Clinical features and treatment outcomes in large granular lymphocytic leukemia (LGLL). **Leuk Lymphoma 2018**, 59(2): 416-422.
10. Barila G, Teramo A, Calabretto G, Vicenzetto C, Gasparini VR, Pavan L, et al. Stat3 mutations impact on overall survival in large granular lymphocyte leukemia: a single-center experience of 205 patients. **Leukemia 2020**, 34(4): 1116-1124.
11. Lamy T, Moignet A, Loughran TJ. LGL leukemia: from pathogenesis to treatment. **Blood 2017**, 129(9): 1082-1094.
12. Yoon HJ, Sugamura K, Loughran TJ. Activation of leukemic large granular lymphocytes by interleukin-2 via the p75 interleukin-2 receptor. **Leukemia 1990**, 4(12): 848-850.
13. Teramo A, Gattazzo C, Passeri F, Lico A, Tasca G, Cabrelle A, et al. Intrinsic and extrinsic mechanisms contribute to maintain the JAK/STAT pathway aberrantly activated in T-type large granular lymphocyte leukemia. **Blood 2013**, 121(19): 3843-3854, S1.
14. Zambello R, Facco M, Trentin L, Sancetta R, Tassinari C, Perin A, et al. Interleukin-15 triggers the proliferation and cytotoxicity of granular lymphocytes

in patients with lymphoproliferative disease of granular lymphocytes. **Blood 1997**, 89(1): 201-211.
15. Mishra A, Liu S, Sams GH, Curphey DP, Santhanam R, Rush LJ, et al. Aberrant overexpression of IL-15 initiates large granular lymphocyte leukemia through chromosomal instability and DNA hypermethylation. **Cancer Cell 2012**, 22(5): 645-655.
16. Kothapalli R, Nyland SB, Kusmartseva I, Bailey RD, Mckeown TM, Loughran TJ. Constitutive production of proinflammatory cytokines RANTES, MIP-1beta and IL-18 characterizes LGL leukemia. **Int J Oncol 2005**, 26(2): 529-535.
17. Yang J, Liu X, Nyland SB, Zhang R, Ryland LK, Broeg K, et al. Platelet-derived growth factor mediates survival of leukemic large granular lymphocytes via an autocrine regulatory pathway. **Blood 2010**, 115(1): 51-60.
18. Sun H, Wei S, Yang L. Dysfunction of immune system in the development of large granular lymphocyte leukemia. **Hematology 2019**, 24(1): 139-147.
19. Moignet A, Lamy T. Latest Advances in the Diagnosis and Treatment of Large Granular Lymphocytic Leukemia. **Am Soc Clin Oncol Educ Book 2018**, 38: 616-625.
20. Dhodapkar MV, Li CY, Lust JA, Tefferi A, Phyliky RL. Clinical spectrum of clonal proliferations of T-large granular lymphocytes: a T-cell clonopathy of undetermined significance? **Blood 1994**, 84(5): 1620-1627.
21. Cheon H, Dziewulska KH, Moosic KB, Olson KC, Gru AA, Feith DJ, et al. Advances in the Diagnosis and Treatment of Large Granular Lymphocytic Leukemia. **Curr Hematol Malig Rep 2020**, 15(2): 103-112.
22. Lamy T, Moignet A, Loughran TJ. LGL leukemia: from pathogenesis to treatment. **Blood 2017**, 129(9): 1082-1094.
23. Sanikommu SR, Clemente MJ, Chomczynski P, Afable MN, Jerez A, Thota S, et al. Clinical features and treatment outcomes in large granular lymphocytic leukemia (LGLL). **Leuk Lymphoma 2018**, 59(2): 416-422.
24. Bareau B, Rey J, Hamidou M, Donadieu J, Morcet J, Reman O, et al. Analysis of a French cohort of patients with large granular lymphocyte leukemia: a report on 229 cases. **Haematologica 2010**, 95(9): 1534-1541.
25. Loughran TJ, Zickl L, Olson TL, Wang V, Zhang D, Rajala HL, et al. Immunosuppressive therapy of LGL leukemia: prospective multicenter phase II study by the Eastern Cooperative Oncology Group (E5998). **Leukemia 2015**, 29(4): 886-894.
26. Qiu ZY, Fan L, Wang R, Gale RP, Liang HJ, Wang M, et al. Methotrexate therapy of T-cell large granular lymphocytic leukemia impact of STAT3 mutation. **Oncotarget 2016**, 7(38): 61419-61425.
27. Moignet A, Hasanali Z, Zambello R, Pavan L, Bareau B, Tournilhac O, et al. Cyclophosphamide as a first-line therapy in LGL leukemia. **Leukemia 2014**, 28(5): 1134-1136.
28. Poullot E, Zambello R, Leblanc F, Bareau B, De March E, Roussel M, et al. Chronic natural killer lymphoproliferative disorders: characteristics of an international cohort of 70 patients. **Ann Oncol 2014**, 25(10): 2030-2035.

29. Dhodapkar MV, Lust JA, Phyliky RL. T-cell large granular lymphocytic leukemia and pure red cell aplasia in a patient with type I autoimmune polyendocrinopathy: response to immunosuppressive therapy. **Mayo Clin Proc 1994**, 69(11): 1085-1088.
30. Zhu Y, Gao Q, Hu J, Liu X, Guan D, Zhang F. Clinical features and treatment outcomes in patients with T-cell large granular lymphocytic leukemia: A single-institution experience. **Leuk Res 2020**, 90: 106299.
31. Osuji N, Matutes E, Tjonnfjord G, Grech H, Del GI, Wotherspoon A, et al. T-cell large granular lymphocyte leukemia: A report on the treatment of 29 patients and a review of the literature. **Cancer-Am Cancer Soc 2006**, 107(3): 570-578.
32. Lamy T, Loughran TJ. How I treat LGL leukemia. **Blood 2011**, 117(10): 2764-2774.
33. Matutes E. Large granular lymphocytic leukemia. Current diagnostic and therapeutic approaches and novel treatment options. **Expert Rev Hematol 2017**, 10(3): 251-258.
34. D'Amato RJ, Loughnan MS, Flynn E, Folkman J. Thalidomide is an inhibitor of angiogenesis. **Proc Natl Acad Sci U S A 1994**, 91(9): 4082-4085.
35. Moreira AL, Sampaio EP, Zmuidzinas A, Frindt P, Smith KA, Kaplan G. Thalidomide exerts its inhibitory action on tumor necrosis factor alpha by enhancing mRNA degradation. **J Exp Med 1993**, 177(6): 1675-1680.
36. Sampaio EP, Sarno EN, Galilly R, Cohn ZA, Kaplan G. Thalidomide selectively inhibits tumor necrosis factor alpha production by stimulated human monocytes. **J Exp Med 1991**, 173(3): 699-703.
37. Singhal S, Mehta J, Desikan R, Ayers D, Roberson P, Eddlemon P, et al. Antitumor activity of thalidomide in refractory multiple myeloma. **N Engl J Med 1999**, 341(21): 1565-1571.
38. Richardson PG, Schlossman RL, Weller E, Hideshima T, Mitsiades C, Davies F, et al. Immunomodulatory drug CC-5013 overcomes drug resistance and is well tolerated in patients with relapsed multiple myeloma. **Blood 2002**, 100(9): 3063-3067.
39. Dimopoulos MA, Zomas A, Viniou NA, Grigoraki V, Galani E, Matsouka C, et al. Treatment of Waldenstrom's macroglobulinemia with thalidomide. **J Clin Oncol 2001**, 19(16): 3596-3601.
40. Seldin DC, Choufani EB, Dember LM, Wiesman JF, Berk JL, Falk RH, et al. Tolerability and efficacy of thalidomide for the treatment of patients with light chain-associated (AL) amyloidosis. **Clin Lymphoma 2003**, 3(4): 241-246.
41. Palladini G, Perfetti V, Perlini S, Obici L, Lavatelli F, Caccialanza R, et al. The combination of thalidomide and intermediate-dose dexamethasone is an effective but toxic treatment for patients with primary amyloidosis (AL). **Blood 2005**, 105(7): 2949-2951.
42. Barosi G, Grossi A, Comotti B, Musto P, Gamba G, Marchetti M. Safety and efficacy of thalidomide in patients with myelofibrosis with myeloid metaplasia. **Br J Haematol 2001**, 114(1): 78-83.
43. Canepa L, Ballerini F, Varaldo R, Quintino S, Reni L, Clavio M, et al. Thalidomide

in agnogenic and secondary myelofibrosis. **Br J Haematol 2001**, 115(2): 313-315.
44. Elliott MA, Mesa RA, Li CY, Hook CC, Ansell SM, Levitt RM, et al. Thalidomide treatment in myelofibrosis with myeloid metaplasia. **Br J Haematol 2002**, 117(2): 288-296.
45. Piccaluga PP, Visani G, Pileri SA, Ascani S, Grafone T, Isidori A, et al. Clinical efficacy and antiangiogenic activity of thalidomide in myelofibrosis with myeloid metaplasia. A pilot study. **Leukemia 2002**, 16(9): 1609-1614.
46. Mesa RA, Steensma DP, Pardanani A, Li CY, Elliott M, Kaufmann SH, et al. A phase 2 trial of combination low-dose thalidomide and prednisone for the treatment of myelofibrosis with myeloid metaplasia. **Blood 2003**, 101(7): 2534-2541.
47. Mesa RA, Elliott MA, Faoro L, Tefferi A. Durable and unmaintained remissions with thalidomide-based drug therapy in myelofibrosis with myeloid metaplasia: Long term outcome analysis of 2 prospective trials.; 2003 2003-01-01; 2003. p. 33A.
48. Raza A, Meyer P, Dutt D, Zorat F, Lisak L, Nascimben F, et al. Thalidomide produces transfusion independence in long-standing refractory anemias of patients with myelodysplastic syndromes. **Blood 2001**, 98(4): 958-965.
49. Zorat F, Shetty V, Dutt D, Lisak L, Nascimben F, Allampallam K, et al. The clinical and biological effects of thalidomide in patients with myelodysplastic syndromes. **Br J Haematol 2001**, 115(4): 881-894.
50. Strupp C, Germing U, Aivado M, Misgeld E, Haas R, Gattermann N. Thalidomide for the treatment of patients with myelodysplastic syndromes. **Leukemia 2002**, 16(1): 1-6.
51. Steins MB, Padro T, Bieker R, Ruiz S, Kropff M, Kienast J, et al. Efficacy and safety of thalidomide in patients with acute myeloid leukemia. **Blood 2002**, 99(3): 834-839.
52. Thomas DA, Estey E, Giles FJ, Faderl S, Cortes J, Keating M, et al. Single agent thalidomide in patients with relapsed or refractory acute myeloid leukaemia. **Br J Haematol 2003**, 123(3): 436-441.
53. Teramo A, Gattazzo C, Passeri F, Lico A, Tasca G, Cabrelle A, et al. Intrinsic and extrinsic mechanisms contribute to maintain the JAK/STAT pathway aberrantly activated in T-type large granular lymphocyte leukemia. **Blood 2013**, 121(19): 3843-3854, S1.
54. Thomas S, Fisher KH, Snowden JA, Danson SJ, Brown S, Zeidler MP. Methotrexate Is a JAK/STAT Pathway Inhibitor. **Plos One 2015**, 10(7): e130078.
55. Loughran TJ, Kidd PG, Starkebaum G. Treatment of large granular lymphocyte leukemia with oral low-dose methotrexate. **Blood 1994**, 84(7): 2164-2170.
56. Bareau B, Rey J, Hamidou M, Donadieu J, Morcet J, Reman O, et al. Analysis of a French cohort of patients with large granular lymphocyte leukemia: a report on 229 cases. **Haematologica 2010**, 95(9): 1534-1541.
57. Wechalekar AD, Goodman HJ, Lachmann HJ, Offer M, Hawkins PN, Gillmore JD. Safety and efficacy of risk-adapted cyclophosphamide, thalidomide, and dexamethasone in systemic AL amyloidosis. **Blood 2007**, 109(2): 457-464.
58. Zhang L, Zhao AL, Duan MH, Li ZY, Cao XX, Feng J, et al. Phase 2 study using

oral thalidomide-cyclophosphamide-prednisone for idiopathic multicentric Castleman disease. **Blood 2019**, 133(16): 1720-1728.

# 17. Appendices

## Appendix 1. ECOG Performance Status

| Grade | ECOG |
|---|---|
| 0 | Fully active, able to carry on all pre-disease performance without restriction |
| 1 | Restricted in physically strenuous activity but ambulatory and able to carry out work of a light or sedentary nature, e.g., light housework, office work |
| 2 | Ambulatory and capable of all self-care but unable to carry out any work activities. Up and about more than 50% of waking hours |
| 3 | Capable of only limited self-care, confined to bed or chair more than 50% of waking hours |
| 4 | Completely disabled. Cannot carry on any selfcare. Totally confined to bed or chair |
| 5 | Dead |

Abbreviation: ECOG, Eastern Cooperative Oncology Group.

# Appendix 2. Thrombosis Risk Assessment Form (Caprini Model)

| A1 1 point per risk factor | B 2 points per risk factor |
|---|---|
| □□ Age 40–59 years old<br>□□ Minor surgery planned<br>□□ History of prior major surgery<br>□□ Obesity (BMI > 30 kg/m$^2$)<br>□□ Medical patient at bed rest<br>□□ History of inflammatory bowel disease<br>□□ Swollen legs<br>□□ Varicose vein<br>□□ Serious lung disease, including pneumonia (<1 month)<br>□□ Abnormal pulmonary function (chronic obstructive pulmonary disease)<br>□□ Acute myocardial infarction (<1 month)<br>□□ Congestive heart failure (<1 month)<br>□□ Sepsis (<1 month)<br>□□ Blood transfusion (<1 month)<br>□□ Plaster casting or fixation of lower limb<br>□□ Central venous cannulation<br>□□ Other risk factors | □□ Age 60–74 years old<br>□□ Major surgery < 60 min<br>□□ Laparoscopic surgery > 60 min<br>□□ Arthroscopic surgery > 60 min<br>□□ Previous malignancy<br>□□ Obesity (BMI > 40 kg/m$^2$)<br>**C 3 points per risk factor**<br>□□ Age > 75 years old<br>□□ Major surgery 2–3 h<br>□□ Obesity (BMI > 50 kg/m$^2$)<br>□□ History of superficial/deep vein thrombosis or pulmonary embolism<br>□□ Family history of thrombosis<br>□□ Current malignancy or chemotherapy<br>□□ Heparin-induced thrombocytopenia<br>□□ Congenital or acquired thrombosis not listed<br>□□ Anticardiolipin antibodies positive<br>□□ Prothrombin 20210A positive<br>□□ Factor Vleiden positive<br>□□ Lupus anticoagulant positive<br>□□ Elevated serum homocysteine |
| A2 For women only (1 point each) | D 5 points per risk factor |
| □□ Oral contraceptives or hormone replacement therapy<br>□□ Pregnancy or postpartum (< 1 month)<br>□□ History of unexplained stillborn infant, recurrent spontaneous abortion ≥3, premature birth with toxemia or growth-restricted infant | □□ Stroke (<1 month)<br>□□ Acute spinal cord injury (<1 month)<br>□□ Elective lower extremity arthroplasty<br>□□ Hip, pelvis or leg fracture<br>□□ Multiple traumas (<1 month)<br>□□ Major surgery > 3 h |
| □□ Total score of risk factors: | |

Notes:① The weight of each risk factor depends on the possibility of causing thrombotic events (for example, if the score of cancer is 3 points and that of bed rest is 1, the former is more likely to cause thrombus than the latter); ② Only one surgical factor can be selected.

## Appendix 3. Calculation of Creatinine Clearance

Cockcroft-Gault formula:

The patient's creatinine clearance (CrCl) is calculated using the following Cockcroft-Gault formula:

CrCl = 1.222 × [(140-age) × body weight (kg)]/serum creatinine (µmol/L)

Multiply by 0.85 in case of a female patient

If serum creatinine is expressed in the International System of Units (SI) (ie, µmol/L), convert SI units to conventional units (mg/dL) using the following formula (Manual of Laboratory Testing and Diagnosis, 2004):

- Serum creatinine (µmol/L) ÷ 88.4 = serum creatinine (mg/dL)

## Appendix 4. Thalidomide Neurotoxicity Grading and Dose Adjustment

| | 0 | 1 | 2 | 3 | 4 |
|---|---|---|---|---|---|
| | Normal | Asymptomatic: Loss of deep tendon reflexes or paresthesia (including tingling), but not interfering with function | Sensory alteration or paresthesia (including tingling) interfering with function but not with activities of daily living | Sensory alteration or paresthesia interfering with activities of daily living | Disabling |
| Not applicable | No action | Thalidomide reduced by one dose level | Thalidomide reduced by two dose levels | Thalidomide withheld | Thalidomide withheld |
| Slight pain but not interfering with function | No action | Thalidomide reduced by two dose levels | Thalidomide discontinued | Thalidomide withheld | Thalidomide withheld |
| Moderate pain (interfering with function but not with activities of daily living) | Thalidomide reduced by one dose level | Thalidomide reduced by two dose levels | Thalidomide withheld | Thalidomide withheld | Thalidomide withheld |
| Severe pain (interfering with activities of daily living) | Thalidomide reduced by one dose level | Thalidomide reduced by two dose levels | Thalidomide withheld | Thalidomide withheld | Thalidomide withheld |
| Disabling | Thalidomide withheld | Thalidomide withheld | Thalidomide withheld | Thalidomide withheld | Thalidomide withheld |

Notes:

Withheld: Thalidomide should be withheld for 2 weeks until the toxicity returns to Grade 1 or better condition.

Reduced by one dose level: Thalidomide reduced from 100 mg/d to 50 mg/d.

Reduced by two dose levels: Thalidomide reduced from 100 mg/d to 50 mg, every other day.
